# AFFF on fire: new insights into the thermal fate of the AFFF compound Capstone B

**DOI:** 10.1007/s00216-026-06657-1

**Published:** 2026-07-11

**Authors:** Melanie Schüßler, Boris Bugsel, Bernat Oró-Nolla, Christian Zwiener

**Affiliations:** 1 Environmental Analytical Chemistry, Department of Geosciences, University of Tübingen, Schnarrenbergstraße 94-96, 72076 Tübingen, Germany; 2 TZW, DVGW-Technologiezentrum Wasser (German Water Centre), Karlsruher Straße 84, 76139 Karlsruhe, Germany

**Keywords:** AFFF, PFAS, Thermal transformation, Pyrolysis, HRMS, Non-target screening, Identification

## Abstract

The widespread use and uncontrolled release of per- and polyfluoroalkyl substances (PFAS) have led to numerous environmental contamination hotspots in the past. Frequently, these contaminations result from fire-fighting measures employing PFAS-containing aqueous film-forming foams (AFFF). Remediation of these sites is often expensive and generates large amounts of waste. Thermal remediation methods have been discussed as an economically viable option for PFAS-contaminated materials and soils. During thermal treatment, depending on the applied temperature and original PFAS, the formation of products of incomplete destruction (PIDs) occurs. These PIDs are problematic as they have not been characterized well in the past for AFFF PFAS and can accumulate during thermal treatment. We studied the thermal fate of AFFF PFAS 6:2 FTSAm-Pr-B, also known as Capstone B, under pyrolytic conditions over a temperature range from 100 to 800 °C. PIDs were characterized by liquid and gas chromatography coupled to (high-resolution) mass spectrometry (LC-(HR)MS and GC-HRMS). The degree of mineralization was determined by fluoride production and ranged between 5.3% at 200 °C and 61% at 600 °C. In total, we identified 55 PIDs over a temperature range from 100 to 400 °C (levels 1–4). At 600 and 800 °C, only fluoride was detected. The majority of the PIDs (47) was detected by nontarget screening (NTS) and PIDs were classified in five substance groups: fluorotelomer sulfonic acids (FTSAs), fluorotelomer sulfonamides (FTSAms), hydroxy-carboxylic acids (OH-CAs), unknown polyfluoro-hydroxy alkyls (PFOHs), and others. A mass balance (MB) was established by molar fluorine equivalents of the (semi)quantified PIDs. The MB showed that transformation processes of 6:2 FTSAm-Pr-B already started at temperatures above 150 °C. Between 200 and 400 °C, PIDs play a significant role and accounted for 8.5% of the fluorine MB at maximum.

**Graphical abstract:**

**Figure:**
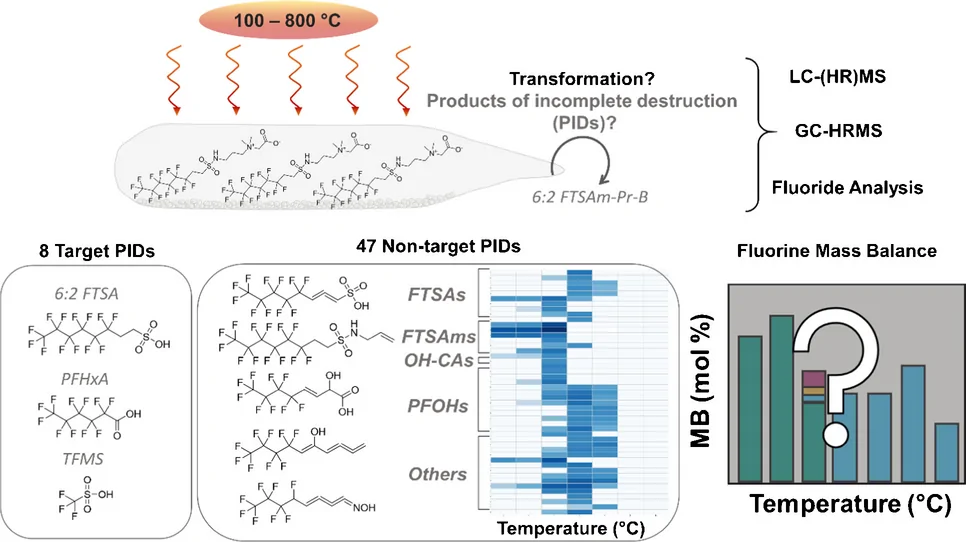

**Supplementary Information:**

The online version contains supplementary material available at https://doi.org/10.1007/s00216-026-06657-1.

## Introduction

Due to their incredibly wide range of applications [1], and their increasing use since the 1940 s, per- and polyfluoroalkyl substances (PFAS) are ubiquitously present at background levels in surface water [2–5], groundwater [6, 7], drinking water [4, 7–9] and soil [10, 11]. Heavily contaminated sites, where PFAS levels can be several magnitudes higher, can be found all over the planet [6, 10, 12]. Possible sources of environmental PFAS contamination can be diverse [12–15] but often originate from proximity to a military site [16–18], airport [19], or civilian fire-fighting measures [20] involving the use of aqueous film-forming foams (AFFF) [21].

These polluted hotspots can pose a significant risk to drinking water resources as PFAS are known to have several adverse health effects on humans [22] and contamination levels can remain stable in environmental compartments for decades due to the high stability of the C-F bond [23–25]. Therefore, active intervention is required to remediate these sites to prevent (further) leaching into groundwater bodies. This is evident from the contamination case in Reilingen (Germany) where, following a major fire event in 2008, large quantities of AFFF were released during fire-fighting operations. Fifteen years later, a nontarget screening (NTS) approach revealed that 124 individual PFAS were still present in the topsoil [20]. Clean-up of contaminated hotspots like the Reilingen site can be achieved by facilitating the immobilization, mobilization, or transformation of PFAS [12]. Immobilization and mobilization techniques in turn generate PFAS-contaminated materials that have to be further treated or deposited. The United States Environmental Protection Agency (US EPA) recently recognized thermal treatment (i.e., incineration) as one of the three economically viable options for PFAS waste management [26]. Incineration is typically carried out ex-situ in waste incineration plants, where PFAS-contaminated material is commonly heated up to > 1000 °C with a residence time of ~ 2 s (s) and the goal of mineralization [27]. PFAS mineralization products include carbon dioxide (CO_2_), hydrofluoric acid (HF), water (H_2_O), and, depending on the presence of further heteroatoms, sulfur, nitrogen, or phosphorus oxides [28]. In contrast, pyrolysis involves thermal treatment of PFAS or PFAS-contaminated materials in the absence of oxygen. This process is often applied to PFAS-containing biosolids at a typical temperature range between 150 and 600 °C, yielding biochar as a product [27, 29]. Thermal desorption (TD) is an approach which applies low to moderate temperatures (100–500 °C) [27] to facilitate physical desorption of particle-bound PFAS and subsequent transfer into the gas phase followed by resorption onto a filter material [12, 30]. However, the thermal processes described may result in the formation of different products of incomplete destruction (PIDs) [28, 31–34] in dependence of initial PFAS characteristics. For example, thermal transformation of perfluoroalkyl acids (PFAAs) can already be initiated between 150 and 400 °C under pyrolytic conditions [35–38], producing perfluorocarbon (PFCs) and hydrofluorocarbon (HFCs) PIDs resulting from initial headgroup loss [39, 40]. These PIDs can pose a kinetic bottleneck in the mineralization process as they require temperatures above 950 °C to be further transformed [29, 41].

In contrast to PFAAs, the structures of AFFF PFAS currently in use are mostly characterized by polyfluorinated and non-fluorinated moieties and only a limited number of studies investigating thermal transformation and PID formation of AFFF PFAS exist. In a study by Xiao et al. (2021), heating of AFFF samples led to a wide range of PIDs emerging between 200 and 400 °C such as fluorotelomer sulfonamides (FTSAms), fluorotelomer sulfonic acids (FTSAs), perfluoroalkyl sulfonamides (PFASAms), or PFAAs [42]. In another study by Yao et al. (2022), pyrolysis of AFFF samples between 200 and 890 °C yielded PIDs like perfluoroalkanes and perfluoroalkenes, perfluoroalkyl aldehydes, fluorotelomer alcohols (FTOHs), and other polyfluorinated compounds [43]. The PIDs of thermal processes must therefore be identified and examined very carefully to ensure that remediation measures at contaminated sites do not result in shifting the problem toward even more unknown contaminants. Therefore, the success of the treatment should not only be assessed on the basis of the removal efficiency (RE) of the initial PFAS, but should also take into account the formation of PIDs, their further transformation, and the formation of mineralization products like fluoride (F^−^). Therefore, fluorine mass balance (MB) approaches are viable tools at laboratory and field scale. For future remediation approaches at hotspots like the Reilingen site, where mixtures of many different PFAS are present, it is imperative to investigate and understand the thermal transformation processes of single AFFF PFAS. In this study, we extensively studied the thermal transformation of 6:2 fluorotelomer sulfonamide propyl betaine (6:2 FTSAm-Pr-B), an AFFF PFAS also known as Capstone B. 6:2 FTSAm-Pr-B was chosen as a model compound because it is one of the most abundant PFAS in contemporary AFFF [44]. The investigation covered a temperature range from 100 to 800 °C under pyrolytic conditions. PIDs were measured by target analysis and nontarget screening (NTS) on two chromatographic platforms (liquid and gas chromatography (LC and GC)). A fluorine MB was accomplished by F^−^ analysis.

## Materials and methods

### Chemicals and reagents

Methanol (MeOH), ammonium acetate (NH_4_Ac), and water were of optimal LC-MS grade and were purchased from Thermo Fisher Scientific. Silica particles (SiO_2_, 0.062–0.2 mm) and Na_2_CO_3_ were acquired from Merck Millipore, and NaHCO_3_ was purchased from Carl Roth. For F^−^ analysis by ion chromatography (IC), an anion multi standard (Supelco) was used. For target analysis, an analytical standard mix containing 43 single PFAS was used (ESM 1.A Table S1). For spiking, the same 6:2 FTSAm-Pr-B standard as listed in Table S1 was used. A standard mix containing 30 isotopically PFAS compounds was used for LC-Orbitrap analysis (ESM 1.A Table S2).

### Thermal transformation experiments

Thermal transformation experiments of 6:2 FTSAm-Pr-B were conducted in in-house custom-produced quartz glass ampoules (dimensions ca: height = 8 cm, diameter = 0.5 cm). SiO_2_ particles (100 mg, prewashed with MeOH and dried at 105 °C for 3 h (h)) were weighed in and spiked with 0.5 mL of a 1 g/L 6:2 FTSAm-Pr-B standard in MeOH (heated samples and non-heated spiked controls). Heated blind controls without 6:2 FTSAm-Pr-B were prepared by adding 0.5 mL of pure MeOH to the SiO_2_ particles. All samples were dried under vacuum in a desiccator (10 mbar) over night to fully evaporate the solvent MeOH. The quartz glass ampoules were sealed by melting the narrow section using a hand torch under vacuum (10^–3^ mbar). To avoid thermal transformation during this process, the ampoules were cooled with liquid nitrogen. Thermal transformation experiments were conducted in a pre-heated muffle furnace at 100, 150, 200, 300, 400, 600, and 800 °C. The applied temperatures cover the temperature range typically employed in thermal desorption (100–500 °C) and pyrolytic (150–600 °C) processes [27]. The closed ampoules were placed in the furnace for 45 min at the respective temperature. For each temperature, four spiked replicates were prepared and placed in the furnace together with two heated blind controls. Also, non-heated spiked controls were prepared to determine the mass balance (MB). Samples were extracted as follows:

Ampoules were opened after a 30-min cooldown, and the contents as well as the broken glass ampoules were transferred to 15 mL polypropylene (PP) centrifuge tubes. For F^–^ analysis by ion chromatography (IC), half of the samples (two of the spiked and heated replicates, one blind control and one non-heated spike control per temperature) were extracted with 10 mL of an aqueous carbonate-bicarbonate buffer solution (Na_2_CO_3_/NaHCO_3_, 3 mM). The remaining samples were extracted with 10 mL of MeOH. After addition of the respective extraction solvent, samples were sonicated for 1 h and centrifuged for 30 min at 7197 relative centrifugal force (rcf). The supernatant was either transferred to another 15 mL PP centrifuge tube (Na_2_CO_3_/NaHCO_3_ extracts) or to 1.5 mL PP LC-MS vials (MeOH extracts). Samples analyzed for F^−^ via IC were filtered through a regenerated cellulose filter (0.45 µm) before analysis.

### Instrumental analysis

#### Fluoride (F^−^) analysis

F^–^ analysis in Na_2_CO_3_/NaHCO_3_ extracts was achieved by using anion-exchange chromatography with a conductivity detector (930 Compact IC Flex, Metrohm). The chromatographic column was a Metrosep A Supp 5–150/4 (Metrohm). The eluent was aqueous Na_2_CO_3_/NaHCO_3_ (3.2 mM/1 mM) at an isocratic flow rate of 0.7 mL/min and a pressure of 9.46 MPa at 20 °C. The suppressor was regenerated using H_2_SO_4_/C_2_H_2_O_4_ (500 mmol/L/20 mmol/L). Injection volume was 20 µL and the calibration range was linear between 0.02 and 2 mg/L.

#### PFAS target analysis

Analysis of target compounds in MeOH extracts was performed using a high-performance liquid chromatography (HPLC, 1290 Infinity II, Agilent Technologies, Waldbronn, Germany) coupled to a 6490 triple quadrupole mass spectrometer (6490 iFunnel QqQ-MS, Agilent Technologies, Santa Clara, USA). To detect trifluoroacetic acid (TFA) and other ultra-short chain acids (USC), a newly, in-house developed method employing an Atlantis BEH C_18_ AX mixed-mode column (100 × 2.1 mm, 1.7 µm) with an integrated guard column was used for analyte separation [45]. The column oven temperature was set to 35 °C and injection volume was 10 µL. A gradient elution with eluents A (H_2_O + 2 mM NH_4_Ac) and B (MeOH + 2 mM NH_4_Ac + 0.02% NH_4_OH) was applied with a total run time of 24 min and a flow rate of 0.3 mL/min (see ESM 1.A Table S3 for gradient program). The QqQ-MS was operated in multiple reaction monitoring mode (MRM) for 22 target analytes. Mass transitions and collision energies (CEs) are given in the ESM (ESM 1.A, Table S4). For quantification, a linear calibration function was prepared from a PFAS mix containing 43 authentic reference standards (ESM 1.A, Table S1). The calibration function was based on eight calibration levels in the range from 0.05 to 10 µg/L and analyzed at the beginning and at the end of each sample sequence (approximately 50 samples). Additionally, for quality control, a solvent blank (50/50 MeOH/H_2_O) and a 0.5 µg/L calibration level were analyzed after every 10 samples.

#### PFAS GC-HRMS analysis

A gas chromatograph (7890B GC-System, Agilent Technologies) equipped with an autosampler (7693 Autosampler, Agilent Technologies) coupled to a quadrupole time-of-flight high-resolution mass spectrometer with an atmospheric pressure chemical ionization source (6550 QTOF and G3212-65100 APCI, Agilent Technologies, GC-APCI-QTOF-HRMS) was used for analysis of volatile compounds in MeOH extracts. For chromatographic separation, a Rtx-200ms GC capillary column (Restek, 30 m, 0.25 mm, 1 µm) was employed. Injection mode was pulsed splitless and injection volume was 1 µL. The temperature gradient started at 40 °C with a hold time of 2 min, following a linear increase at a rate of 10 °C/min to 200 °C, and a subsequent heating rate of 25 °C/min to 240 °C with a hold time of 2 min, resulting in a total runtime of 22 min. The transfer line to the APCI was kept constant at 260 °C. The APCI was operated with a corona current of 3 µA in positive ionization mode and the QTOF-MS was operated in scan mode with an acquisition rate of 1 spectrum/s. In order to obtain MS^2^ information for compounds of interest, the QTOF was operated in targeted MS^2^ mode with an acquisition rate of 1 spectra/s in MS^1^ and 3 spectra/s in MS^2^ mode. Collision energy (CE) was determined according to Eq. 1:

1 $$CE=\frac{3\ast m/z}{100}+15\;eV$$

A calibration curve containing 6:2 and 8:2 FTOH in a concentration range between 1 and 200 µg/L was prepared and quality controls were injected every 10 samples to account for instrument drift during the sample sequence.

#### PFAS LC-HRMS analysis

MeOH extracts were analyzed by HPLC (Vanquish Flex, Thermo Fisher Scientific) coupled to an Orbitrap Exploris 120 with an electrospray ionization source (Thermo Fisher Scientific, HPLC-ESI-Orbitrap-HRMS) in positive and negative ionization mode (ESI +/–). For chromatographic separation, an Agilent C_18_ analytical column (Poroshell 120 EC-C_18_, 2.1 mm × 100 mm, particle size 2.7 µm) was used. Column oven temperature was set to 40 °C and injection volume was 5 µL. A gradient elution (total run time 23 min) with eluent A (95/5 H_2_O/MeOH + 2 mM NH_4_Ac) and eluent B (95/5 MeOH/H_2_O + 2 mM NH_4_Ac) and a flow rate of 0.3 mL/min were applied (see ESM 1.A, Table S5 for gradient program). As 6:2 FTSAm-Pr-B is a zwitterionic substance, PIDs with anionic, cationic, and zwitterionic character were expected. Thus, each sample was injected twice and analyzed once in positive and once in negative ionization mode (ESI +/ESI–). Orbitrap data acquisition was performed in data-dependent MS^2^ mode. Resolution in MS^1^ was 60,000 (FWHM at m/z 200) and in MS^2^ 15,000 (FWHM at m/z 200). The threshold for MS^2^ triggering was 10,000 counts and dynamic exclusion (after 2 triggered MS^2^ events for 2 s), isotope exclusion and apex detection (desired apex window 30%) were applied. For collision induced dissociation (CID), four different CEs were utilized (setpoints at 10, 40, 100, 160 V) and resulting MS^2^ spectra were averaged over the CE range (mean CE = 77.5 V).

Samples were measured in different dilutions depending on the treatment temperature (undiluted, 1:10, 1:100, 1:1000, 1:10,000). In samples with high concentrations of the precursor 6:2 FTSAm-Pr-B (100 °C, 150 °C, 200 °C), the fraction of the retention time range of 6:2 FTSAm-Pr-B was generously directed into the waste (5.5–6.2 min). This might have resulted in some PIDs eluting during this period being overlooked.

To account for instrument drift over the sequence, a standard mix containing 30 isotopically labelled PFAS (1 µL of a 10 µg/L mix) was added to each sample before measurement. Relative standard deviation (RSD) of peak areas was below 30% for all but two compounds (6:2/6:2 diPAP and 8:2/8:2 diPAP) over the sequence (ESM 1.A, Table S2).

For semi-quantification, three calibration points (0.1 µg/L, 5 µg/L, and 10 µg/L) of a native PFAS standard mix containing 43 PFAS were analyzed together with the samples.

Analytes were matched for semi-quantification with a PFAS standard with a matching functional group. Response factors (RF) of standard compounds were determined according to Eq. 2 and averaged over the three calibration points. For more information about semi-quantification, refer to the ESM 1.A (Table S6 and Table S7).

2 $$RF=\frac{Peak\;area}{Concentration}$$

### Screening for products of incomplete destruction (PIDs)

Evaluation of LC- and GC-HRMS data was exclusively done via open-source software. Sample alignment was facilitated by *MSDial* (version 5.5.250404) [46] and included the files of the non-spiked heated controls to account for features emerging solely due to the heating of the SiO_2_ particles. For data visualization, *mzmine 4.7.8* [47] was utilized. PF∆*Screen* [48] was used for feature prioritization and identification. Details on settings and parameters of all three software programs can be found in ESM 1.B, Tables S8–S10. As previously shown, potential PFAS features can be efficiently prioritized by slicing the PF∆*Screen* output table at specific mass-to-carbon ratio (m/C value) and retention time (RT) values [20]. However, to account for possible defluorination during thermal transformation, the values were slightly adjusted (m/C > 21 and RT > 3 min). For PFAS suspect screening with PF∆*Screen*, the suspect list from the National Institute of Standards and Technology (NIST) [49] was employed. Identification of prioritized features and suspect hits included ascertaining in which samples a feature occurred (sample alignment with *MSDial*) and determining the polarity of a feature by checking for its presence in both polarities in *mzmine*. Also, in the *Raw Data Visualization* section of PF∆*Screen*, it was determined, if the feature was part of a homologous series or correlated with other features at the same RT (*R*^2^ > 0.97) and thus might be an in-source fragment or an adduct. MS^2^ spectra from *mzmine* were interpreted with the help of PF∆*Screen* in case diagnostic mass fragment differences or typical PFAS neutral losses were annotated, otherwise manually. When a chemical formula was assigned to a feature either through a suspect hit or through manual MS^2^ spectra interpretation, the isotopic pattern match was calculated as followed [20, 50]:

3 $$Isotopic\;pattern\;match=\left(1-\left|\left(I_{M+1}^{theoritical}\right)-\left(I_{M+1}^{measured}\right)\right|\right)\cdot\left(1-\left|\left(I_{M+2}^{theoritical}\right)-\left(I_{M+2}^{measured}\right)\right|\right)$$

where *I* corresponds to the peak areas of the theoretical and measured *M+1 *and *M+2 *peaks, normalized to the *M* peak.

Identified PIDs were classified into confidence levels modified from Charbonnet et al. [20, 51]. For all identified PIDs, the discrepancy between theoretical and measured m/z and m/C was determined as well as the isotopic pattern matches according to Eq. 3. All identified NTS PIDs can be found in the ESM 1.D and ESM 2. Note that all identified PIDs via nontarget screening (NTS) were identified by LC-Orbitrap-HRMS while for the GC-QTOF data, no NTS PIDs could be identified.

## Results and discussion

### Pyrolytic transformation of 6:2 FTSAm-Pr-B and formation of hydrofluoric acid (HF)

**Fig. 1:**
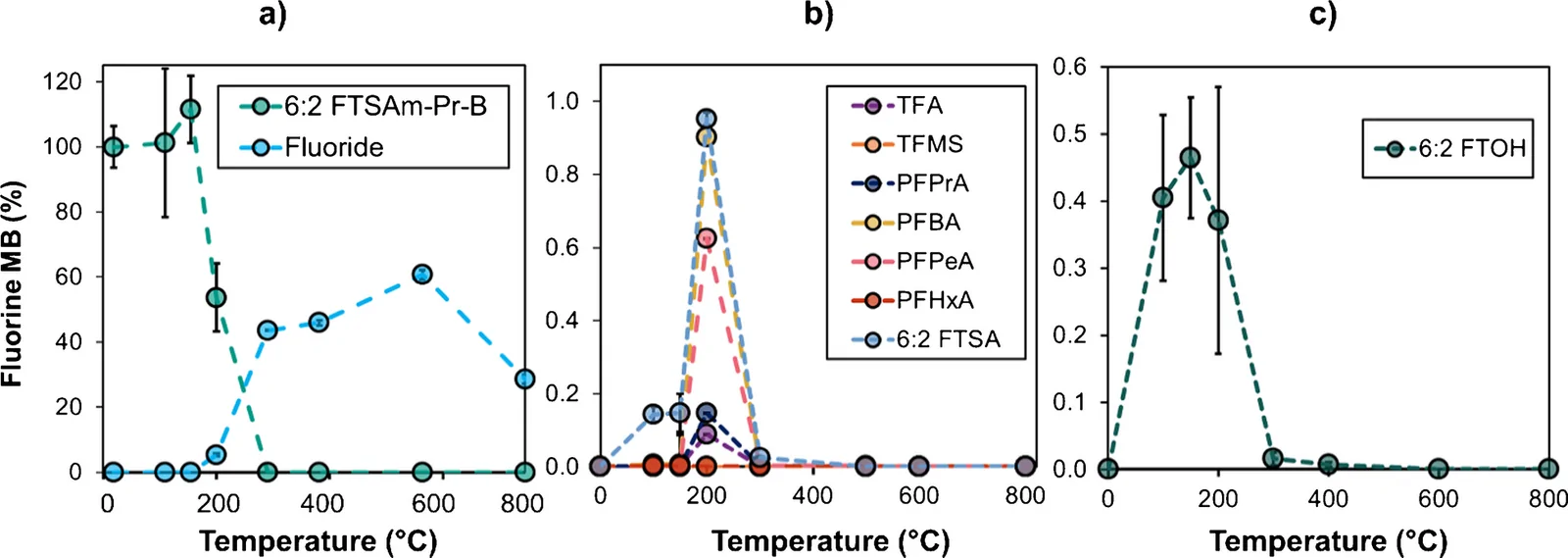
(**a**) Decrease of 6:2 FTSAm-Pr-B and increase of F^−^ over the studied temperature range (100–800 °C). Formation of the identified target PIDs by (**b**) LC-QqQ-MS and (**c**) GC-qToF-MS. Error bars represent the standard deviation (*s* = │*x*_1_−*x*_2_│/√ 2) estimated for duplicates

For a fluorine mass balance (MB), all concentrations were converted into molar fluorine equivalents and normalized to the initial concentration of 6:2 FTSAm-Pr-B. During heating, the concentration of 6:2 FTSAm-Pr-B remained unchanged up to 150 °C. At 200 °C, about 48% of fluorine was still present as 6:2 FTSAm-Pr-B while at 300 °C and above molar F^−^ equivalents of 6:2 FTSAm-Pr-B were well below 0.01% (Fig. 1a). Correspondingly, the formation of HF (quantified as F^−^) started at 200 °C (5%), steeply increased to 44% at 300 °C and reached a maximum of 61% at 600 °C. Interestingly, the share of HF decreased to 29% at 800 °C which suggests a further reaction in which the F^−^ is consumed. The gap in the fluorine MB between 200 and 600 °C indicates the formation of PIDs during the transformation of 6:2 FTSAm-Pr-B (Fig. 1a).

### Identification of products of incomplete destruction (PIDs)

**Fig. 2:**
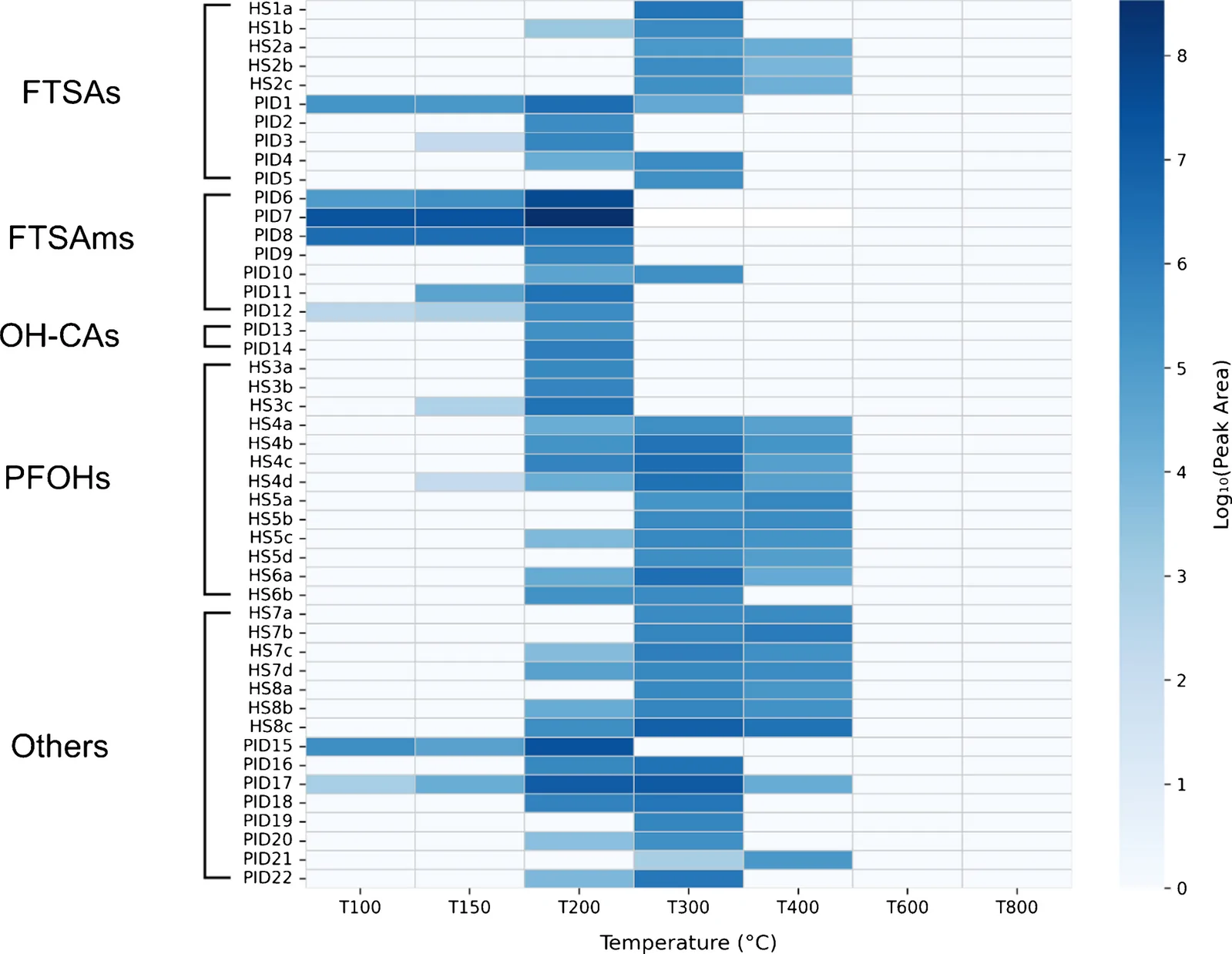
Logarithmic scale heatmap of peak areas for all identified pyrolysis PIDs of 6:2 FTSAm-Pr-B via NTS (NTS PIDs) over the investigated temperature range of 100–800 °C and classification into subclasses according to ionizing group

In total, 55 PIDs of 6:2 FTSAm-Pr-B could be identified. Only eight target PIDs were detected by LC-QqQ (Fig. 1b) and GC-HRMS (Fig. 1c)), while 47 PIDs were identified by LC-HRMS and NTS (Fig. 2, Table 1).

### Target PIDs identified by LC-QqQ and GC-HRMS

Identified target PIDs included short-chain and ultra-short chain PFCAs (perfluorinated chain lengths C_1_–C_5_), trifluoromethane sulfonic acid (TFMS), 6:2 FTSA (all identified by LC-QqQ, Fig. 1b), as well as 6:2 FTOH (identified by GC-QTOF, Fig. 1c). All identified target PIDs appeared between 100 and 300 °C and reached maximum fractions of the fluorine MB at 150 or 200 °C (Fig. 1b and c). Even at 800 °C, small amounts of TFMS (5 × 10^–4^% of F MB) could still be detected. 6:2 FTSA, PFBA, and PFPeA were the three most abundant target PIDs, but together, they accounted for only 2.48% of the fluorine MB at 200 °C (Fig. 1b). TFA, TFMS, PFPrA, and PFHxA further contributed together to less than 0.3% of the MB. The significantly higher abundance of (short chain) PFCAs compared to PFSAs is consistent with previous findings [42, 52] and may arise from multiple formation pathways for PFCAs which can be produced via radical-mediated mechanisms involving random-chain or end-chain scission of the perfluorinated alkyl chain of precursors [42, 53]. Also, hydrolysis of intermediate aldehydes or acyl fluorides to PFCAs occurs upon exposure to moisture or solvents during extraction [40, 41, 52, 54].

It was shown by Xiao et al. (2021) that the formation of PFOS from thermal transformation of PFOSB, a perfluorinated, C_8_-based analogue to 6:2 FTSAm-Pr-B, is possible by side-chain stripping, a step-wise oxidation of the non-fluorinated moiety [42]. As TFMS was the only PFSA detected in this study, it is hypothesized that 6:2 FTSA, rather than PFHxS, is the dominant intermediate in this reaction, due to the telomeric nature of 6:2 FTSAm-Pr-B compared to PFOSB.

Xiao et al. (2021) further demonstrated that PFOS may also be formed from thermal transformation of 8:2 FTSA due to recombination reactions of perfluorinated radicals and the sulfonic acid moiety [42]. However, no PFHxS was detected in our experiments, suggesting that this transformation pathway for 6:2 FTSA likely did not occur. Nevertheless, the formation of perfluorinated radicals cannot be confirmed or ruled out based on the analytical approach used in this study, as their detection was beyond the scope of the applied analytical methods.

6:2 FTOH was tentatively identified by GC-APCI-QTOF-HRMS analysis and confirmed with a respective analytical standard (Fig. 1c). The temperature profile of 6:2 FTOH followed a similar trend than the identified PIDs found by LC-QqQ-MS; however, it was slightly shifted to lower temperatures (0.4% F MB at 100 °C) as it reached its maximum abundance already at 150 °C (0.46% F MB) with a further drop to 0.37% at 200 °C and even 0.02% at 300 °C. In contrast, significant concentrations of PIDs found by LC-QqQ-MS occurred solely at 200 °C with the exception of 6:2 FTSA which was already detected from 100 °C (Fig. 1b). FTOHs were previously detected as PIDs in studies investigating thermal decomposition of AFFF [43, 52, 53]. In addition, Riedel et al. (2021) observed an HF loss from FTOHs upon thermal transformation indicating that HF-losses and the formation of unsaturated FTOHs are possible [53]. No further PIDs could be identified by GC-QTOF-MS, even after applying the NTS workflow.

### PIDs identified by nontarget screening (NTS)

In total, 47 PIDs of 6:2 FTSAm-Pr-B were tentatively identified between 200 and 400 °C by NTS of LC-Orbitrap-MS data (22 single PIDs and 8 homologous series HS with 2 to 4 homologous each, confidence level 1–4, Table 1, ESM 1.D Figs. S2–S48 and ESM 2). The majority (39) of PIDs was ionized in ESI−, while seven PIDs occurred in both, ESI− and ESI+, and only one in ESI+. Eleven PIDs were tentatively identified with a confidence level of 2, 15 at level 3, and 21 PIDs at level 4. The majority of PIDs was most abundant at 200 °C or 300 °C (Fig. 2) while at 600 °C and 800 °C, no PIDs were identified. Regarding the fluorinated carbon chain length, only four PIDs with an intact 6:2 FT chain could be identified via NTS (PIDs 6, 7, 8, and 15). All other PIDs showed fluorinated chain lengths shorter than C_6._

The PIDs could be subdivided into five compound groups according to their functional groups: fluorotelomer sulfonic acids (FTSAs), fluorotelomer sulfonamides (FTSAms), hydroxy-carboxylic acids (OH-CAs), unknown polyfluoro-hydroxyalkyls (PFOHs), and “others.” Chromatographic peaks and MS^2^ spectra of all PIDs can be found in the ESM1.D and ESM 2. In the following paragraphs, the identified NTS PIDs are discussed in detail.

#### FTSA derivatives:

Occurred in all samples from 100 to 400 °C and included two HS and five single PIDs (HS1 and HS2, PIDs 1–5, Fig. 2, Table 1, ESM 1.D Figs. S2–S11). With the exception of 6:2 FTSA which was detected as a target compound (100–300 °C, Fig. 1b), all other identified FTSA derivatives were characterized by a high degree of unsaturation and additional OH-groups. All FTSA PIDs usually showed one or more diagnostic fragments of the SO_3_^−^ group (80.9652: SO_3_H^−^, 79.9574: SO_3_^−^, 63.9624: SO_2_^−^, 94.9808: CH_3_SO_3_^−^). For oxidized FTSA-PIDs with two OH-groups, a neutral loss of H_2_O (∆m/z = 18.0106, HS2, PID2) was observed. Neutral losses of HF (HS1a, PID3, PID5; indicative of proximate C-F and C-H bonds in fluorotelomers) and fragments with the general molecular formula C_x_HF_y_O^−^ (HS1a, PIDs 3–5) and C_x_H_2_F_y_O^−^ (HS1a, PID3, PID5) were also frequently detected within this PID group.

#### FTSAm derivatives:

Encompassed seven single PIDs (PIDs 6–12), including one zwitterionic compound (PID7) and a dimer (PID8) (Fig. 2, Table 1, ESM 1.D Figs. S12–S18). The majority of FTSAm PIDs was generated at temperatures ≤ 200 °C, comprising the three PIDs that retain the full 6:2 FT chain and the sulfonamide moiety (PID6, PID7, PID8), thus displaying the greatest structural similarity to the parent compound 6:2 FTSAm-Pr-B. PIDs 6 and 7 were likely formed by the loss of the alkyl betaine rest of the original 6:2 FTSAm-Pr-B molecule, indicating the proposed side-chain stripping mechanism [42]. MS^2^ spectra of FTSAm PIDs were characterized by several consecutive losses of HF (PIDs 6, 8, 9, 10) and/or characteristic CF_2_ losses (PID9, PID12). The occurrence of the dimer compound (PID8) suggests the recombination of fluorinated radicals into larger polyfluorinated species. Similar dimeric products have been detected in the headspace of heated AFFF samples [52]. Fragments of the sulfonamide group were abundant in negative ionization mode (82.9609: SO_2_F^−^, 64.9703: SO_2_H^−^, 50.0036: C_3_N^−^, 74.0048: C_2_HFNO^−^).

#### Hydroxy-carboxylic acids (OH-CAs):

Include two single PIDs (PID13 and PID14) which both solely appeared at 200 °C (Fig. 2, Table 1, ESM 1.D Figs. S19, S20). Both PIDs included a partly defluorinated, oxidized FT-chain of the precursor 6:2 FTSAm-Pr-B with a terminal carboxylic acid group and an additional hydroxy group (diagnostic fragments at m/z 99.0088: C_4_H_3_O_3_^−^ and 101.0244: C_4_H_5_O_3_^−^). The two PIDs only differ in the presence of two hydrogen atoms (PID13: C_8_H_5_F_9_O_3_, PID14: C_8_H_7_F_9_O_3_) and also showed very similar RT. The possibility of PID13 being an in-source fragment of PID14 was excluded using the PF∆*Screen EIC correlator* and the occurrence of different MS^2^ spectra.

#### Unknown polyfluoro-hydroxyalkyls (PFOHs):

Four homologous series with a polyfluorinated chain and an alkyl-hydroxy rest were detected (HS3, HS4, HS5, HS6, Fig. 2, Table 1, ESM 1.D Figs. S21–S33). Identified PFOH derivatives mainly appeared between 200 and 400 °C with maximum abundances at 300 °C. At first glance, the MS^2^ spectra suggest the occurrence of unsaturated FTOHs and interestingly, the MS^2^ spectra of HS4a, b, c, d and HS5a are very similar to those of hydroxy-carboxylic acids of PIDs 13 and 14. However, it was not possible to ionize and detect a 6:2 FTOH standard with the applied analytical technique (LC-ESI-Orbitrap-HRMS). On the other hand, 6:2 FTOH was detected by GC-APCI-QTOF-MS, but not the four HS. Therefore, we suggest two possible hypotheses for the identity of these compounds: (1) the detected ions may rather be in-source fragments (ISF) of unknown precursors than the original molecular ions which were not detected, or, (2) they may represent products of acyl fluorides, which can be formed during thermal transformation of PFAAs. This would fit to their occurrence at temperatures between 300 and 400 °C (Fig. 2) [33, 54–56]. However, they are thought to be rather short-lived as they are likely to hydrolyze to PFCAs upon contact with moisture [56].

Accordingly, the identity of these PIDs remains unclear and all HS classified within this group were assigned to confidence level 4.

#### Others:

Two HS (HS7 and 8) and eight individual PIDs (PIDs 15–22) were classified within the “others” category (Fig. 2, Table 1, ESM 1.D Figs. S34–S48), which showed the least similarity to the original compound 6:2 FTSAm-Pr-B and cannot be directly derived from it. Six PIDs within this group were zwitterionic and one could be only detected with ESI+ (PID22). The two PIDs 15 and 21 (Fig. 2, Table 1, ESM 1.D Figs. S41, S47) ionized negatively and exhibited a fluorotelomer sulfonyl moiety. PID15 appeared at temperatures < 200 °C with an intact 6:2 FT chain while PID21 appeared at 300 °C and 400 °C and showed stronger divergency from the original 6:2 FTSAm-Pr-B structure with only four fully fluorinated carbons, the presence of several C=C double bonds and a second thio-group incorporated in the molecule. Also, several PIDs within this category include potential aromatic structures (HS3, PID8, ESM 1.D Figs. S40, S48); however, those PIDs were classified as level 4. The formation of cyclic compounds via ring-closure reactions of perfluorinated diradicals was proposed by Altarawneh et al. (2022) for thermal transformation of PFCAs [40] and is thus proposed to be possible for the thermal transformation of 6:2 FTSAm-Pr-B. In addition, most PIDs in the “others” group (all but HS7, PID15, and PID21) exhibited a nitrogen-containing functional group as the ionizing group as indicated by nitrogen-containing fragments (65.9986: C_3_NO^**−**^, 111.0126: C_5_H_6_FNO^−^, 80.0495: C_5_H_6_N^+^) frequently detected in this group.

**Table 1:**
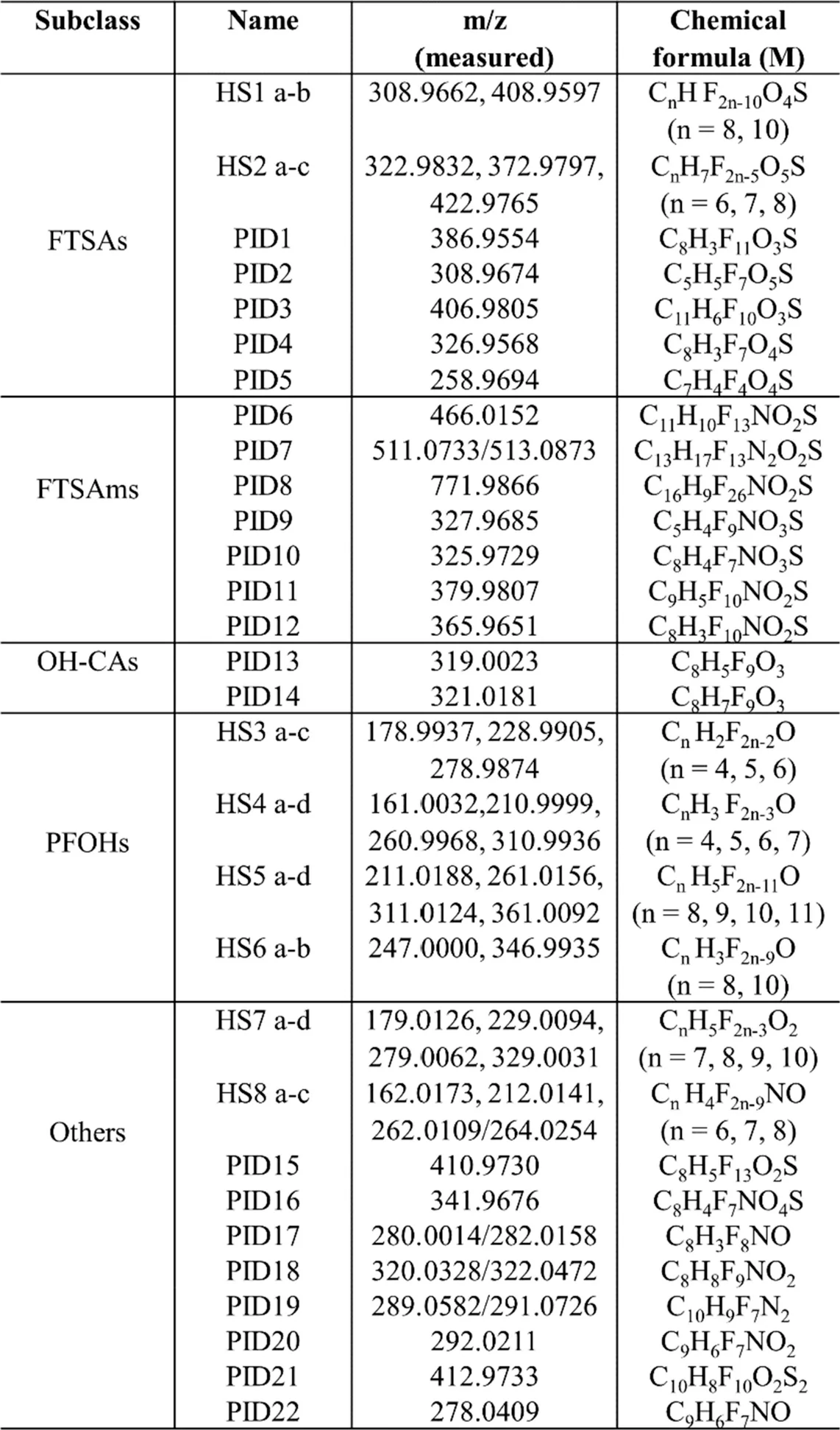
Products of incomplete destruction (PID) tentatively identified by NTS (single PIDs and PIDs in homologous series HS) detected after pyrolysis of 6:2 FTSAm-Pr-B between 200 and 400 °C. Mass to charge ratios (m/z) and tentatively assigned chemical formulas are shown in the table; retention times (RT) and further information can be found in ESM2. Note, that in the case of PFOHs, the displayed chemical formula may not refer to the molecular ion (M) but rather to a detected in-source fragment (ISF)

### Molar fluorine mass balance (MB)

**Fig. 3:**
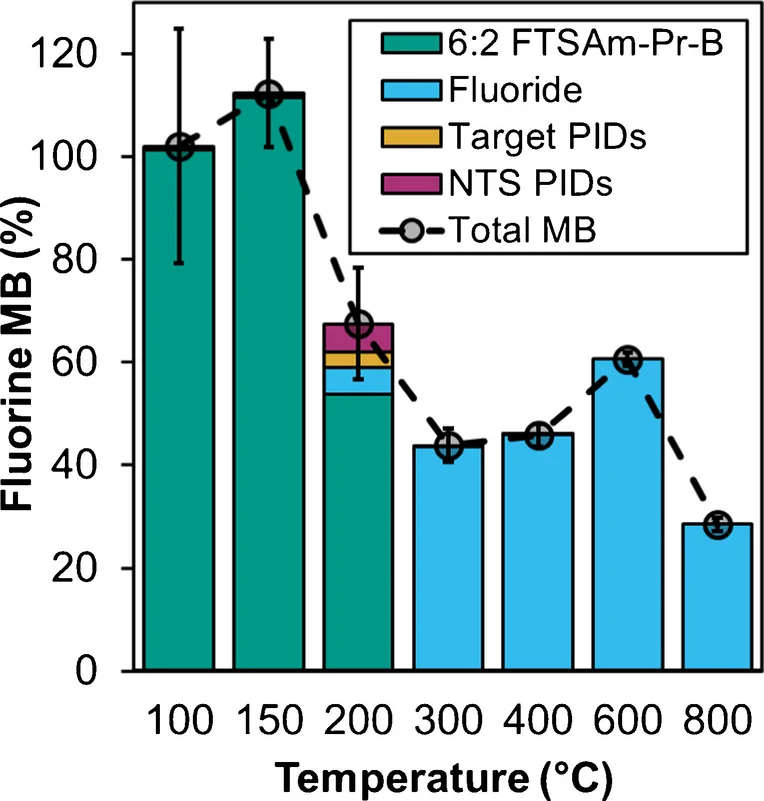
Molar fluorine mass balance (MB) for thermal transformation of 6:2 FTSAm-Pr-B under pyrolytic conditions including hydrofluoric acid (HF) analyzed as F^−^, and the sum of identified target and NTS PIDs. Error bars represent the propagated uncertainty of the duplicates by estimation of the repeatability standard deviation for the duplicates (*s* = │*x*_1_−*x*_2_│/√ 2)

For semi-quantification, only PIDs with a matching standard of the same functional group were considered. This excluded the PFOH derivatives and some compounds in the “others” category. In total, 20 PIDs were semi-quantified (ESM 1.A, Table S7). The fluorine MB was prepared on a molar basis of fluorine equivalents and expressed in percent of the initial molar quantity of 6:2 FTSAm-Pr-B (Fig. 3). The observed temperature profile of the fluorine MB reveals that transformation of 6:2 FTSAm-Pr-B started at about 200 °C and was completely transformed (but not mineralized) at 300 °C. This is consistent with previous studies investigating the thermal transformation of AFFF samples and single AFFF components [36, 42].

At 200 °C, the fluorine MB was still dominated by 6:2 FTSAm-Pr-B (53.7%), but PIDs contributed relevant shares to the MB with 3.1% (target analytes) and 5.4% (nontargets), as well as F^−^ with 5.3% (Fig. 3). Already at 300 °C, the MB was dominated by F^−^ with 43.6% while 6:2 FTSAm-Pr-B was below 0.001% MB and identified PIDs were below 1% (0.05% target PIDs, 0.2% NTS PIDs). It is noteworthy, that the identified NTS PIDs, which were excluded from the semi-quantification, often exhibited their highest abundances between 300 and 400 °C, suggesting an increased, non-quantifiable relevance within this temperature range. The overall fluorine MB remains above 40% over the investigated temperature range due to the application of complementary analytical approaches, with the exception of 800 °C.

Assuming that mineralization efficiency increases with increasing temperature, the decrease of F^−^ between 600 and 800 °C indicates that it must have been consumed by secondary reactions which may involve the SiO_2_ particles. This observation corresponded with the visual changes in the silica particles of the spiked samples after thermal treatment: between 100 and 600 °C, the particles exhibited progressive darkening with increasing temperature, indicative of pyrolytic transformations, whereas at 800 °C they appeared gray and powder-like, suggesting structural disintegration (ESM 1.C, Fig. S1) and thus might have been involved in secondary reactions consuming the F^−^. A similar observation was made by Alinezhad et al. (2022) who studied the thermal transformation of PFOA in the presence of kaolinite and soil and postulated that reactions between the mineral and F radicals were responsible for the decrease in F^−^ yield, compared to thermal treatment with PFOA only, thus forming non-extractable, crystalline phases [36]. Additionally, Krusic et al. (2005) reported that the thermolysis of PFOA can be catalyzed according to the following reaction [57]:

4 $$SiO_2+4\;HF\rightarrow2\;H_2O+SiF_4$$

Consequently, this reaction can be a sink for F^−^, and may increasingly occur at higher temperatures, leading to an (apparent) decrease in detectable F^−^ (from HF formation). Although the HF contribution to the fluorine MB exceeded 40% at temperatures between 300 and 600 °C, complete mineralization was likely not achieved in the investigated temperature range. The remaining gap in the MB conclusively is attributable to (1) incomplete F^−^ recovery, presumably due to the formation of non-solvable SiF_x_ species, in addition to (2) the occurrence of volatile poly- and perfluorinated compounds produced by initial headgroup loss [41] and other volatile intermediates that require temperatures above 950 °C to be completely mineralized [36, 41, 43]. Nevertheless, the substantial F^−^ formation at temperatures > 200 °C, the simultaneous occurrence of multiple homologues of a single HS with varying fluorinated chain lengths at a given temperature, and the detection of several PIDs with a high degree of unsaturation collectively indicate the occurrence of defluorination and chain shortening processes during the pyrolysis of 6:2 FTSAm-Pr-B in a temperature range between 100 and 400 °C.

## Conclusions

This study demonstrated that AFFF component 6:2 FTSAm-Pr-B (“Capstone B”) is rapidly transformed above 200 °C under pyrolytic conditions and produces a variety of products of incomplete destruction (PIDs) between 100 and 400 °C. In total, 55 PIDs were (tentatively) identified by LC-(HR)MS, the majority by an NTS approach. The nontarget PIDs were subdivided into five different subclasses according to the ionizing functional group (FTSAs, FTSAms, carboxylic acids, PFOHs, and others). A fluorine MB could be established based on (semi)-quantification of PIDs, F^−^, and residual 6:2 FTSAm-Pr-B. Already at 150 °C, the occurrence of the two PIDs 6:2 FTSA and 6:2 FTOH indicates decomposition of 6:2 FTSAm-Pr-B, albeit to a minor extent. At 200 °C, 46.3% of 6:2 FTSAm-Pr-B had already transformed, whereas only 5.3% of F^−^ was detected. Target and nontarget PIDs reached their highest contribution to the MB at this temperature, with 3.1% and 5.4%, respectively. At 300 °C, 6:2 FTSAm-Pr-B was completely decomposed and 43.6% could be recovered as F^−^ which further increased to 60.7% at 600 °C.

In conclusion, the results indicate that remediation of AFFF contamination by thermal treatment is generally possible, since polyfluorinated AFFF compounds like 6:2 FTSAm-Pr-B decompose at temperatures above 200 °C, but may also produce a multitude of PIDs. For practical applications, the distribution of formed PIDs may be influenced by the soil matrix properties and the presence of oxygen. Nevertheless, our results demonstrate that well-known PFAS such as 6:2 FTSA and short-chain PFAAs can be formed as PIDs during the thermal transformation of 6:2 FTSAm-Pr-B, which is consistent with previously proposed transformation pathways in the literature. Complete decomposition of the precursor compound at high mineralization levels and minimal proportions of PIDs requires rather high temperatures above 400 °C, which somewhat limits in-situ field applications, especially when soil moisture is taken into account. Furthermore, the gap in the fluorine mass balance suggests the formation of other, potentially volatile PIDs not identified in this work.

## Supplementary Information

Below is the link to the electronic supplementary material.

Supplementary file1 (PDF 8.92 MB)

Supplementary file2 (XLSX 161 KB)

## Data Availability

Data will be made available upon request.
