# Supplementary material for: AFFF on fire: new insights into the thermal fate of the AFFF compound Capstone B
Source: Anal Bioanal Chem. 2026 Jul 11;418(19):6335–46. doi: 10.1007/s00216-026-06657-1 (PMC13585775; doi:10.1007/s00216-026-06657-1)
Supplement: Supplementary file 1 [file 216_2026_6657_MOESM1_ESM.pdf]

Analytical and Bioanalytical Chemistry

Electronic Supplementary Material 1

AFFF on Fire: New insights into the thermal fate of the AFFF compound Capstone B

Melanie Schüßler[1], Boris Bugsel[2], Bernat Oró-Nolla[1], Christian Zwiener[1*]

[1]Environmental Analytical Chemistry, Department of Geosciences, University of Tübingen, Schnarrenbergstraße 94-96, 72076 Tübingen, Germany

[2]TZW, DVGW-Technologiezentrum Wasser (German Water Centre), Karlsruher Straße 84, 76139 Karlsruhe, Germany

[*]*Corresponding author*

Table of Contents

ESM1.A Instrumental Analysis 3

ESM1.B Screening for Transformation products 9

ESM1.C Fluorine Mass Balance 11

ESM 1.D Chromatograms and MS2 spectra of NTS PIDs 12

- **FTSA derivative PIDs** 12
- **FTSAm derivative PIDs** 22
- **Hydroxy carboxylic acid derivative PIDs** 29
- **Unknown polyfluoro-hydroxyalkyl derivative PIDs** 31
- **Other PIDs** 44

References 59

ESM1.A Instrumental Analysis

Table S1: Overview about all PFAS reference standards that were included in the PFAS standard mixture and from where they were purchased.

Substance class	Substance	Origin
PFCAs	TFA	Thermo Fisher Scientific
	PFPrA	Sigma-Aldrich
	PFBA	Wellington Laboratories
	PFPeA	Wellington Laboratories
	PFHxA	Wellington Laboratories
	PFHpA	Wellington Laboratories
	PFOA	Wellington Laboratories
	PFNA	Wellington Laboratories
	PFDA	Wellington Laboratories
	PFUnDA	Wellington Laboratories
	PFDoDA	Wellington Laboratories
	PFTriDA	Wellington Laboratories
	PFTeDA	Wellington Laboratories
	PFHxDA	Wellington Laboratories
	PFODA	Wellington Laboratories
PFSAs	TFMS	Sigma-Aldrich
	PFETs	LGC Standards
	PFPrS	LGC Standards
	PFBS	Wellington Laboratories
	PFPeS	Wellington Laboratories
	PFHxS	Wellington Laboratories
	PFHpS	Wellington Laboratories
	PFOS	Wellington Laboratories
	PFNS	Wellington Laboratories
	PFDS	Wellington Laboratories
	PFDoDs	Wellington Laboratories
PASF-based PFAS	PFHxSAm	Dr. Ehrenstorfer
	PFOSAm	Wellington Laboratories
	N-EtFOSAA	Wellington Laboratories
FTCAs & FTUCAs	6:2 FTCA	Wellington Laboratories
	8:2 FTCA	Wellington Laboratories
	5:3 FTCA	Wellington Laboratories
	7:3 FTCA	Wellington Laboratories
	6:2 FTUCA	Wellington Laboratories
	8:2 FTUCA	Wellington Laboratories
FTSAs	6:2 FTSA	Wellington Laboratories
	8:2 FTSA	Wellington Laboratories
Bis-FASIs	NTf2	Sigma Aldrich
AFFF PFAS	6:2 FTSAm-Pr-DiMeNO	Dr. Ehrenstorfer
	6:2 FTSAm-Pr-B	Dr. Ehrenstorfer
	PFHxSAm-Pr-DiMeAm	Dr. Ehrenstorfer
	5:3 FTB	Wellington Laboratories
	5:1:2 FTB	Wellington Laboratories

Table S2: Overview about all isotopically labelled PFAS reference standards that were included in the internal standard (IS) mixture as well as relative standard deviation (RSD in percent) of the peak area for each internal standard consistent peak areas < 2000 counts.

Substance class	Native compound	Sum formula of labelled compound	RSD (%)
PFCAs	TFA	[13]C2HF3O2	28
	PFPrA	[13]C3HF5O2	15.6
	PFBA	[13]C4HF7O2	17.9
	PFPeA	[13]C5HF9O2	21.8
	PFHxA	[13]C2C4HF11O2	10.3
	PFHpA	[13]C4C3HF13O2	11.8
	PFOA	[13]C4C4HF15O2	11.3
	PFNA	[13]C5C4HF17O2	23.2
	PFDA	[13]C2C8HF19O2	28.8
	PFUnDA	[13]C2C9HF21O2	29.5
	PFDoDA	[13]C2C10HF23O2	23.6
	PFTeDA	[13]C2C12HF27O2	20.5
PFSAs	PFBS	[13]C3CHF9O3S	3.1
	PFHxS	[18]O2C6HF13OS	2.6
	PFOS	[13]C4C4HF17O3S	19.5
FTCAs & FTUCAs	6:2 FTCA	[13]C2C6H3F13O2	20.7
	10:2 FTCA	[13]C2C10H3F21O2	23.5
	10:2 FTUCA	[13]C2C10H2F20O2	22.0
FTSAs	4:2 FTSA	[13]C2C4H5F9O3S	7.1
	6:2 FTSA	[13]C2C6H5F13O3S	20.7
	8:2 FTSA	[13]C2C8H5F17O3S	26.2
	10:2 FTSA	[13]C2C10D4HF21O3S	28.5
PASF-based PFAS	PFOSAm	[13]C8H2F17NO2S	13.8
	N-MeFOSA	C9D3HF17NO2S	19.0
	EtFOSA	C10D5HF17NO2S	19.7
	MeFOSAA	C11D3H3F17NO4S	17.3
	N-EtFOSAA	C12D5H3F17NO4S	18.3
diPAPs	6:2/6:2 diPAP	[13]C4C12H9F26O4P	36.9
	8:2/8:2 diPAP	[13]C4C16H9F34O4P	41.9
Ether PFAS	GenX	[13]C3C3HF11O3	n.d.

Table S3: Gradient program used in the PFAS target analysis via HPLC-QqQ MS employing an Atlantis mixed mode column [1].

Time (min)	A (%)	B (%)
0	99	1
2	99	1
3	75	25
8	50	50
13	15	85
14	1	99
16	1	99
16.1	99	1
24	99	1

Table S4: Analytes included in the target method with their respective mass transitions (qualifier and quantifier) and used collision energies. Note: All analytes but 6:2 FTSAm-Pr-B were analyzed in negative ionization mode. Funnel Voltage was 380 V for all analytes.

Substance class	Analyte	Precursor ion	Product ion	Collision Energy (V)
PFCAs	TFA	113	113	2
		113	69	10
	PFPrA	163	163	2
		163	118.9	10
	PFBA	212.9	212.9	2
		212.9	168.8	5
	PFPeA	262.9	219	5
		262.9	68.8	45
	PFHxA	312.9	268.9	5
		312.9	119	25
	PFHpA	362.8	318.8	5
		362.8	168.7	15
PFSAs	TFMS	149	149	2
		149	79.9	20
	PFEtS	199	98.9	30
		199	79.9	30
	PFPrS	249	98.9	40
		249	80	40
	PFBS	298.9	98.9	30
		298.9	79.8	40
	PFPeS	349	99	30
		349	80	25
	PFHxS	398.8	98.9	45
		398.8	79.9	45
	PFHpS	449	99	45
		449	80	50
	PFOS	499	99	50
		499	79.7	55
FTCAs	5:3 FTCA	341	236.9	10
		341	216.8	25
	6:2 FTCA	377	292.7	20
		377	63	5
FTUCAs	6:2 FTUCA	357	293	10
		357	243	40
FTSAs	6:2 FTSA	427	406.7	20
		427	81.2	55
Bis-FASIs	NTf2	279.9	146.9	25
		279.9	77.9	45
PASF-based	PFHxSAm	397.9	77.9	40
		397.9	47.9	55
AFFF PFAS	6:2 FTSAm-Pr-DiMeNO	527	506.4	5
		527	180.8	10
	6:2 FTSAm-Pr-B	571.1	439.7	30
		571.1	57.6	45

Table S5: Gradient program used in the PFAS non-target analysis via HPLC-Orbitrap MS.

Time (min)	A (%)	B (%)
0	85	15
2	30	70
5	10	90
10	0	100
15	0	100
15.1	85	15
22	85	15

Table S6: Response factors (RF) for standards used for semiquantification and absolute accuracy quotient (AAQ) [2] for the respective standards. Note: for 5:1:2 FTB and 5:3 FTB, 5:2 FTCA and 6:2 FTCA, as well as 6:2 FTSAm-Pr-B and 6:2 FTSAm-Pr-DiMeNO the mean of the response factors respectively was used for semiquantification. In these cases, AAQs were calculated with the mean RFs.

Standards	RF (L/µg)	Polarity	AAQ
5:3 FTB	1.66×10^5	Positive	1.41
5:1:2 FTB	1.2×10^5	Positive	1.88
5:3 FTB & 5:1:2 FTB	**1.43×10^5**	Positive	-
5:3 FTCA	1.94×10^4	Negative	2.42
6:2 FTCA	6.2×10^3	Negative	2.97
5:3 FTCA & 6:2 FTCA	**1.28×10^4**	Negative	-
6:2 FTSA	5.5×10^4	Negative	1.04
PFHxSAm	3.9×10^5	Negative	1.05
PFHxSAm-Pr-DiMeAm	9.17×10^4	Negative	1.12
PFHxSAm-Pr-DiMeAm	2.70×10^5	Positive	1.2
6:2 FTSAm-Pr-B	3.65×10^4	Positive	2.46
6:2 FTSAm-Pr-DiMeNO	1.43×10^5	Positive	4.55
6:2 FTSAm-Pr-B & 6:2 FTSAm-Pr-DiMeNO	**8.99×10^4**	Positive	-

Table S7: Matched analytical reference standards to PIDs for semiquantification.

Subclass	PID	Matched standard	Polarity
FTSA	HS1a-b	6:2 FTSA	Negative
FTSA	HS2a-c	6:2 FTSA	Negative
FTSA	PID1	6:2 FTSA	Negative
FTSA	PID2	6:2 FTSA	Negative
FTSA	PID3	6:2 FTSA	Negative
FTSA	PID4	6:2 FTSA	Negative
FTSA	PID5	6:2 FTSA	Negative
FTSAm	PID6	PFHxSAm-Pr-DiMeAm	Negative
FTSAm	PID7	PFHxSAm-Pr-DiMeAm	Positive
FTSAm	PID8	PFHxSAm	Negative
FTSAm	PID9	PFHxSAm	Negative
FTSAm	PID10	PFHxSAm	Negative
FTSAm	PID11	PFHxSAm	Negative
FTSAm	PID12	PFHxSAm	Negative
Carboxylic acid	PID13	5:3 FTCA & 6:2 FTCA	Negative
Carboxylic acid	PID14	5:3 FTCA & 6:2 FTCA	Negative
Other	PID15	6:2 FTSA	Negative
Other	PID18	5:1:2 FTB & 5:3 FTB	Positive
Other	PID19	6:2 FTSAm-Pr-B & 6:2 FTSAm-Pr-DiMeNO	Positive
Other	PID21	6:2 FTSA	Negative

ESM1.B Screening for Transformation products

Table S8: Overview about all parameters and settings used for data analysis in PFΔ*Screen*

**Parameter**	**Value**
Peak finding	
Mass error (ppm)	10
Intensity threshold	1000
MS/MS alignment	
Mass tolerance (Da)	0.005
RT tolerance (s)	12
Blank correction	
Mass tolerance (Da)	0.002
RT tolerance (s)	6
Fold change	5
MD/C – m/C & MD filtering	
m/C range	≥21
MD/C range	-0.5 – 0.5
MD range	-0.5 -0.5
Kendrick mass defect	
KMD difference	CF_2
KMD mass tolerance	0.002
Number of homologues	3
MS differences and DFs	
Mass differences	CF_2, C_2F_4, HF
Number of fragments	1
Mass tolerance fragments (Da)	0.002
Intensity threshold MS/MS	1000

Table S9: Overview about all parameters and settings used for data alignment in *MSDial.*

**Parameter**	**Value**
Data collection	
MS1 tolerance (Da)	0.005
MS2 tolerance (Da)	0.02
Peak detection	
Minimum peak height	500
Mass slice width (Da)	0.1
Alignment parameters	
Retention time tolerance (min)	0.1
MS1 tolerance (Da)	0.015

Table S10: Overview about all parameters and settings used for the *MZmine* project.

**Parameter**	**Value**
HPLC	
Crop retention time (min)	0.005
Max peaks in chrmoatogram	0.02
Minimum consecutive scans	4
Approximate feature FWHM (min)	0.1
RT-tolerance (intra-sample, min)	0.08
RT tolerance (sample-to-sample, min)	0.4
Orbitrap	
Noise threshold MS1	5
Noise threshold MS2	2.5
Minimum feature height	50000
m/z tolerance (scan-to-scan, Da/ppm)	0.0020/10
m/z tolerance (intra-sample, Da/ppm)	0.0015/3
m/z tolerance (sample-to-sample, Da/ppm)	0.0015/5

ESM1.C Fluorine Mass Balance

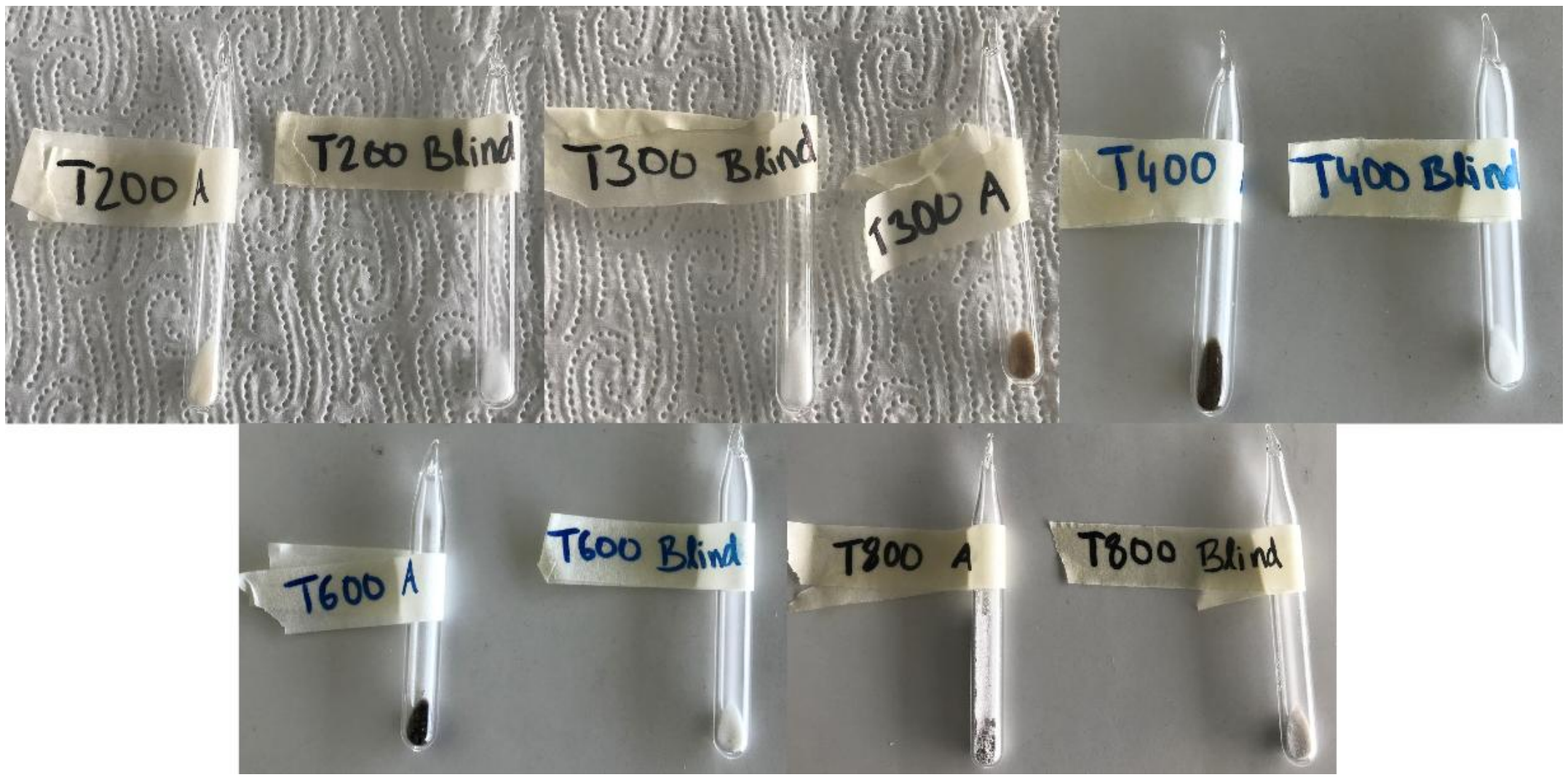

Figure S1: Picture of the ampoules after heating for each temperature. The left ampoule shows one of the spiked heated duplicates and the right ampoule on of the non-spiked heated duplicate for temperatures from 200-800°C, respectively. The picture shows the progressive darkening of the spiked particles until 600 °C and the grey, powder like structure of the spiked, heated particles for the experiment at 800 °C.

ESM 1.D Chromatograms and MS2 spectra of NTS PIDs

FTSA derivative PIDs

HS1a

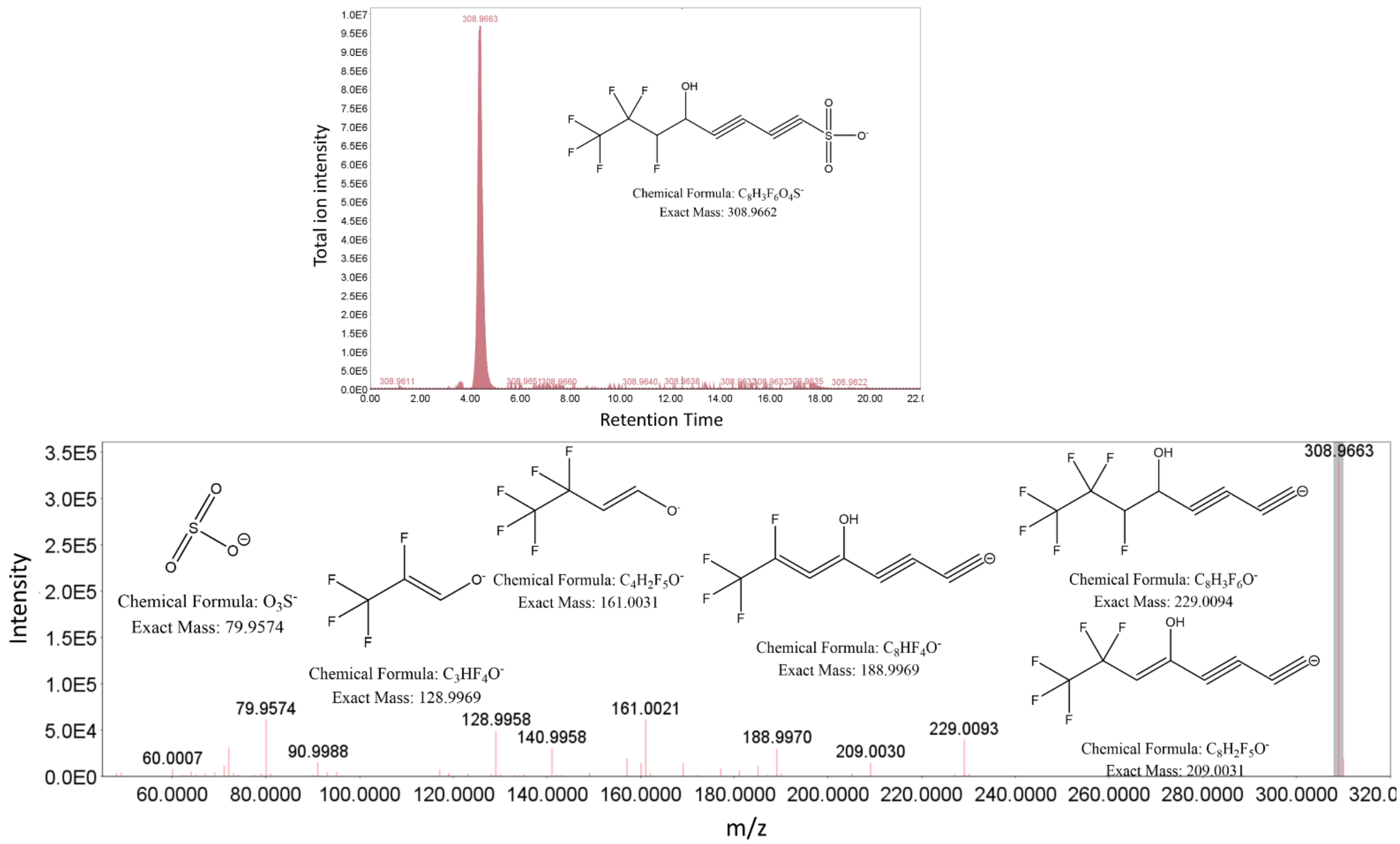

Figure S2: Chromatogram with tentative identified structure and annotated MS^2 spectrum (ESI-, mean collision energy: 77.5 V) of HS1a (m/z = 308.9662, RT = 4.4 min, Confidence Level 3) in MeOH extract of T300 B.

HS1b

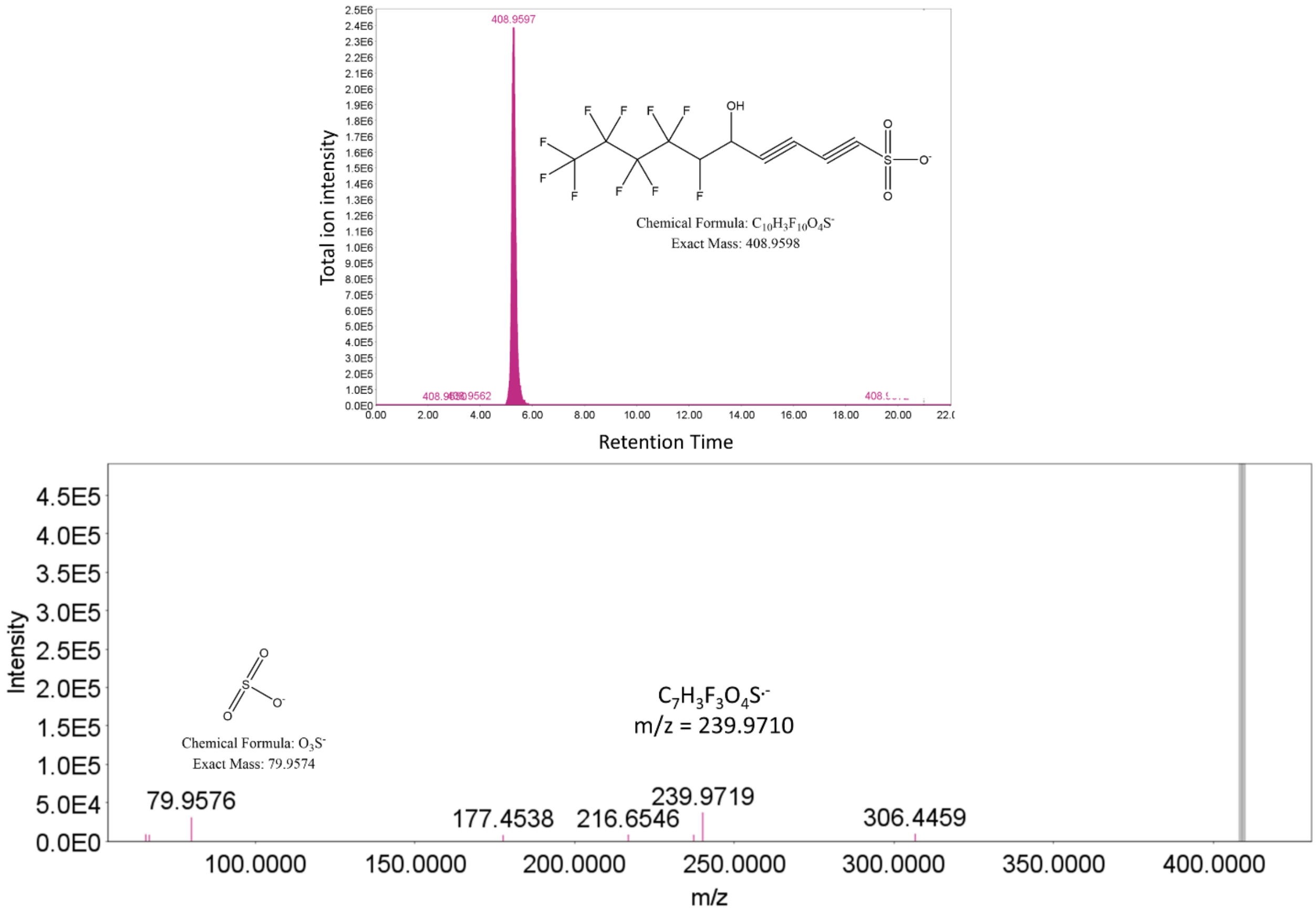

Figure S3: Chromatogram with tentative identified structure and annotated MS^2 spectrum (ESI-, mean collision energy: 77.5 V) of HS1b (m/z = 408.9597, RT = 5.27 min, Confidence Level 3) in MeOH extract of T300 B.

HS2a

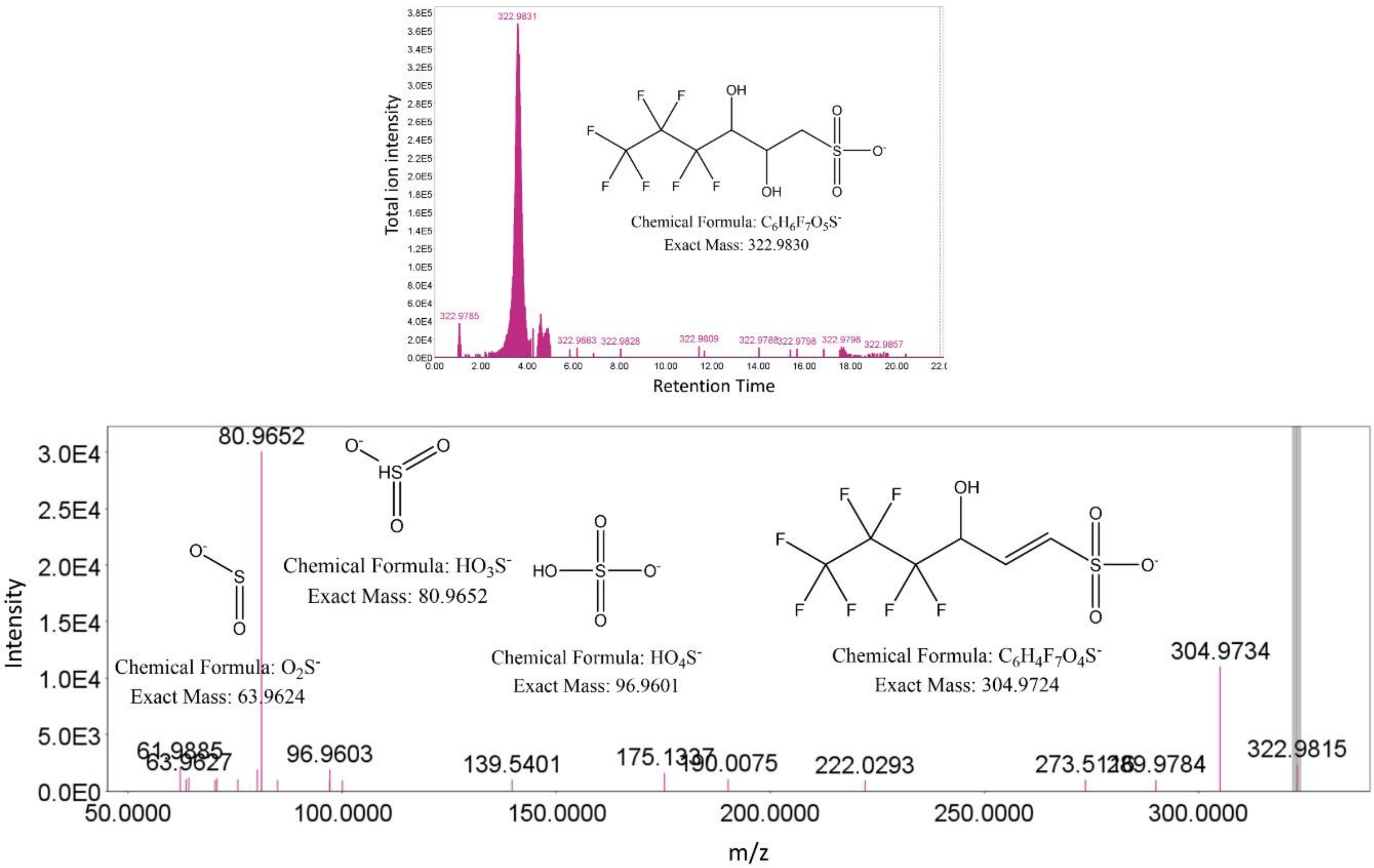

Figure S4: Chromatogram with tentative identified structure and annotated MS^2 spectrum (ESI-, mean collision energy: 77.5 V) of HS2a (m/z = 322.9832, RT = 3.6 min, Confidence Level 3) in MeOH extract of T300 B.

HS2b

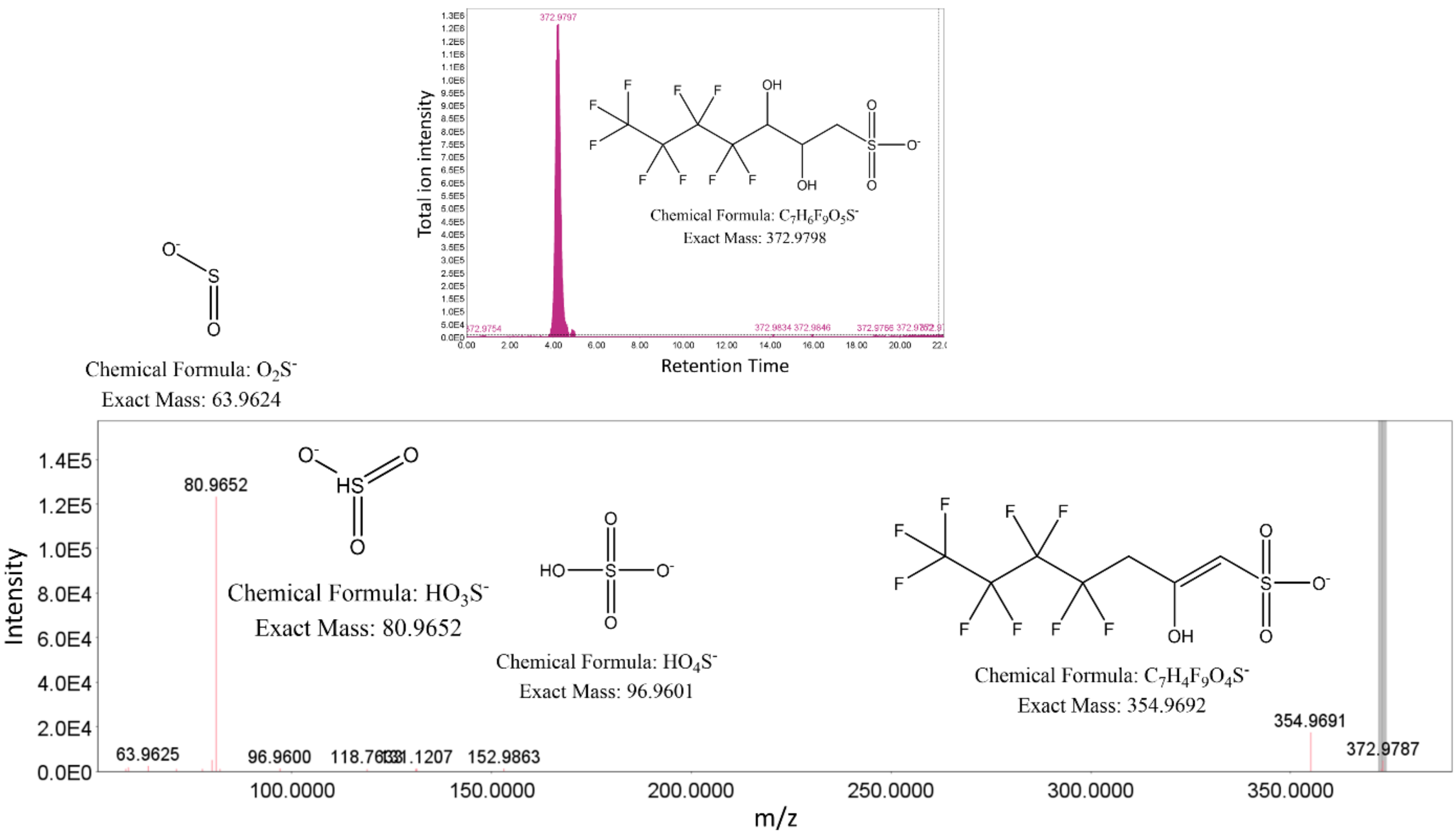

Figure S5: Chromatogram with tentative identified structure and annotated MS^2 spectrum (ESI-, mean collision energy: 77.5 V) of HS2b (m/z = 372.9797, RT = 4.2 min, Confidence Level 3) in MeOH extract of T300 B.

HS2c

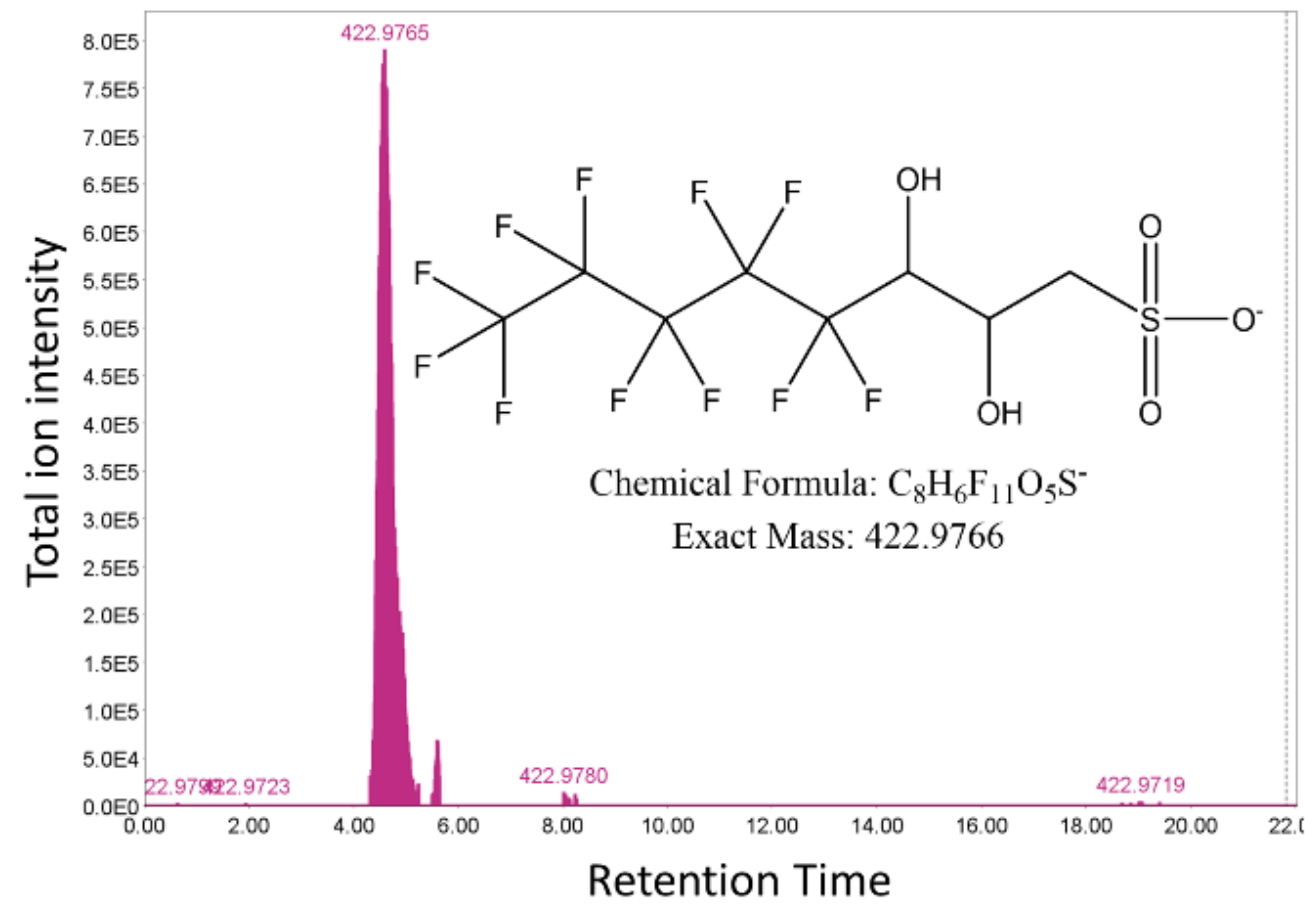

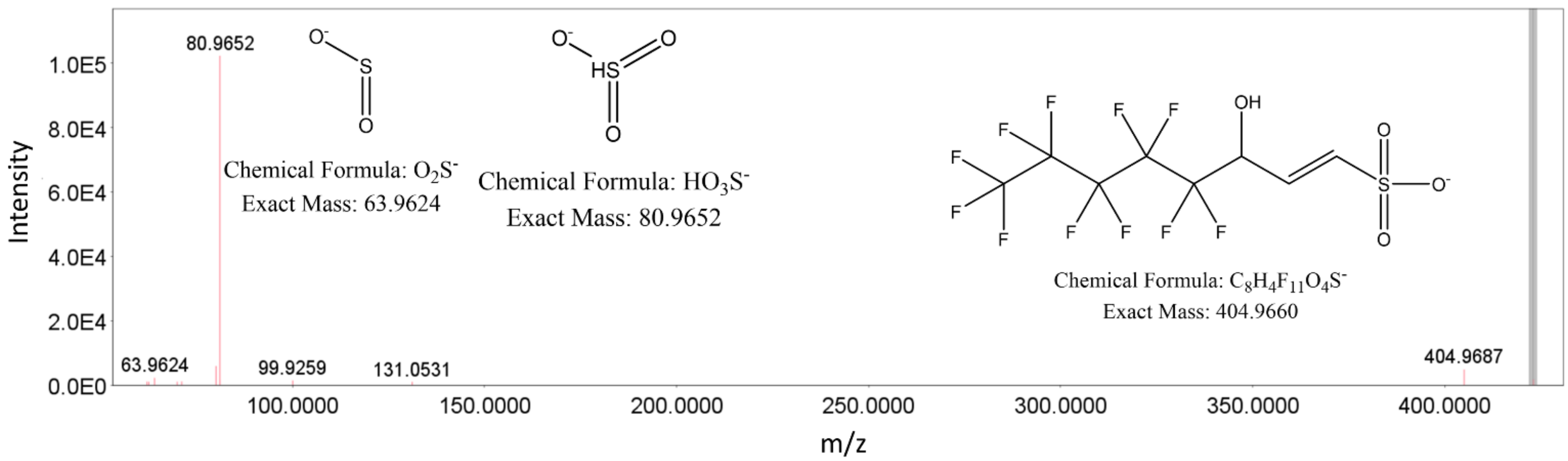

Figure S6: Chromatogram with tentative identified structure (T300 B) and annotated MS^2 spectrum (ESI-, mean collision energy: 77.5 V) of HS2c (m/z = 422.9765, RT = 4.6 min, Confidence Level 3) in MeOH extract of T300 A.

PID1

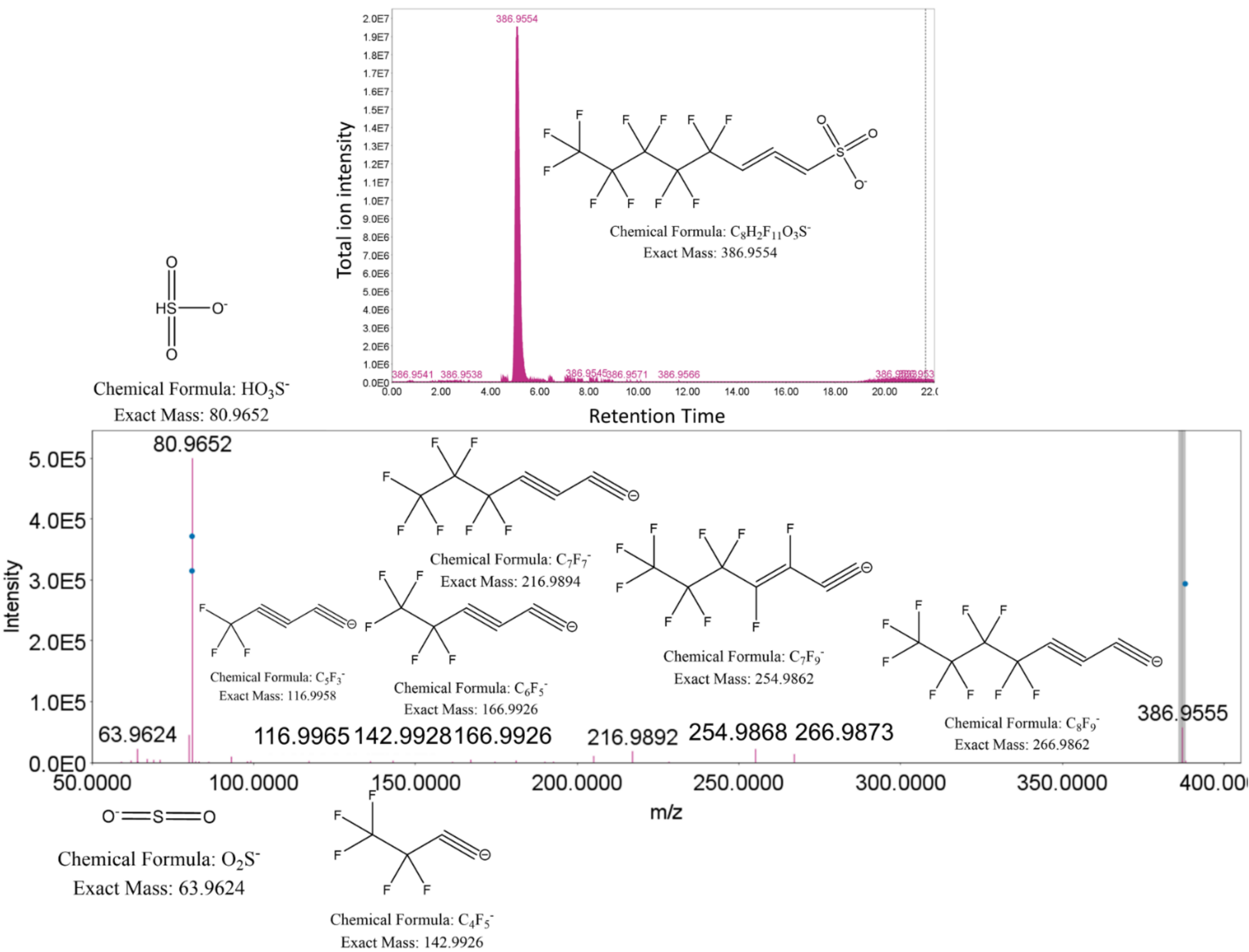

Figure S7: Chromatogram with tentative identified structure and annotated MS^2 spectrum (ESI-, mean collision energy: 77.5 V) of PID1 (m/z = 386.9554, RT = 5.1 min, Confidence level 2) in MeOH extract of T200 B.

PID2

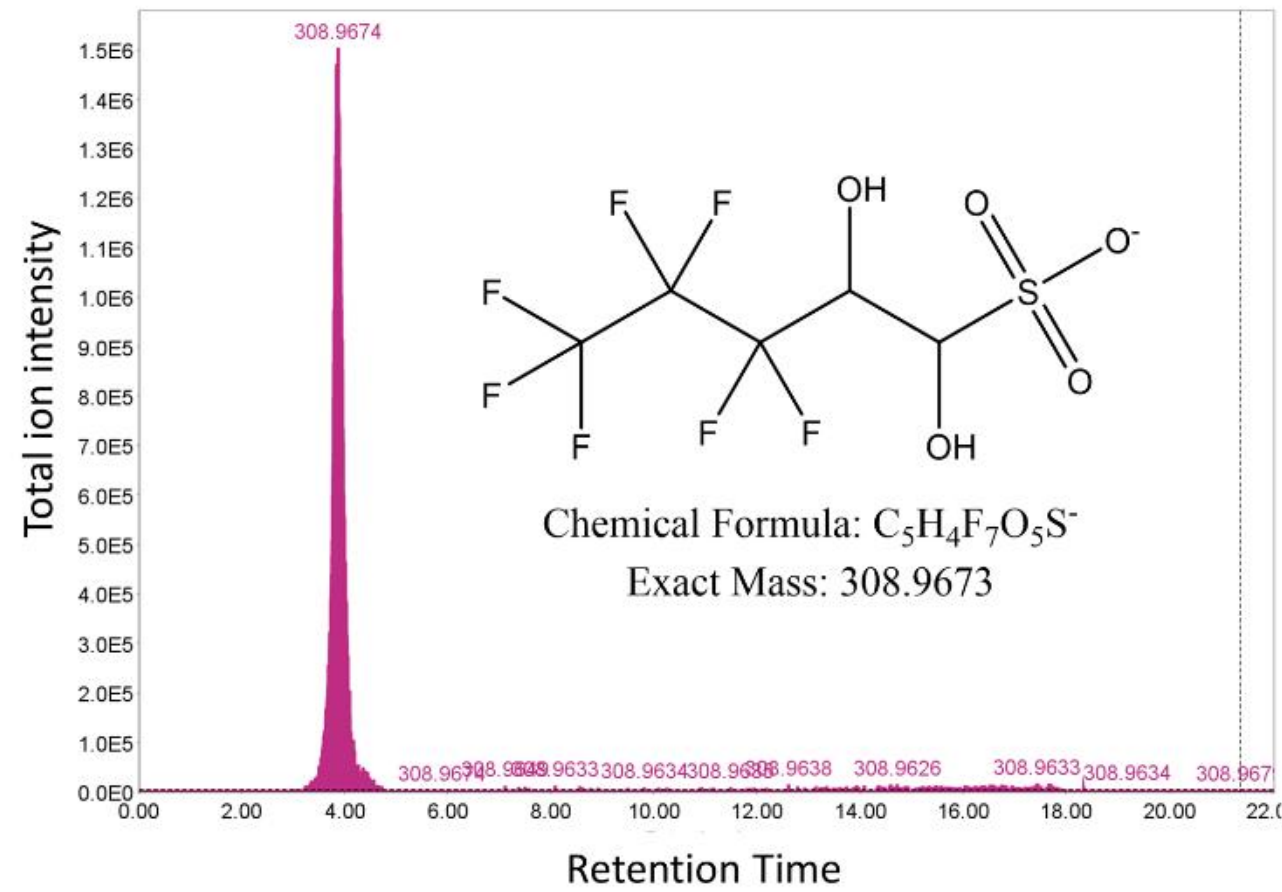

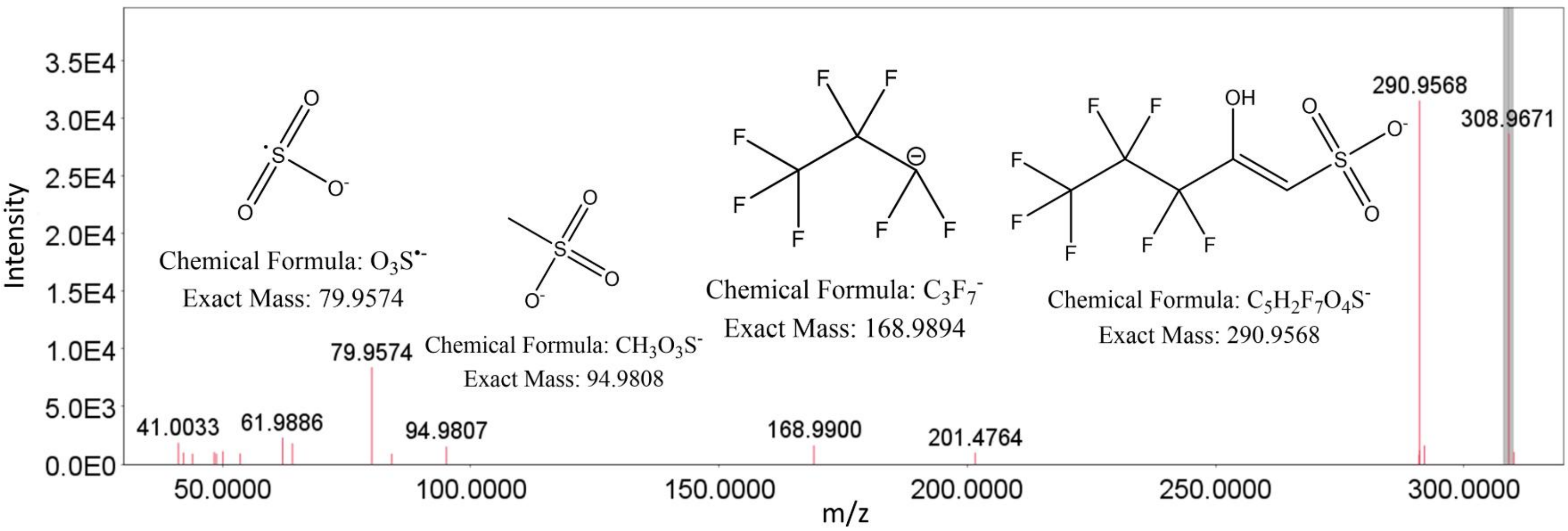

Figure S8: Chromatogram with tentative identified structure and annotated MS^2 spectrum (ESI-, mean collision energy: 77.5 V) of PID2 (m/z = 308.9674, RT = 3.9 min, Confidence Level 2) in MeOH extract of T200 B.

PID3

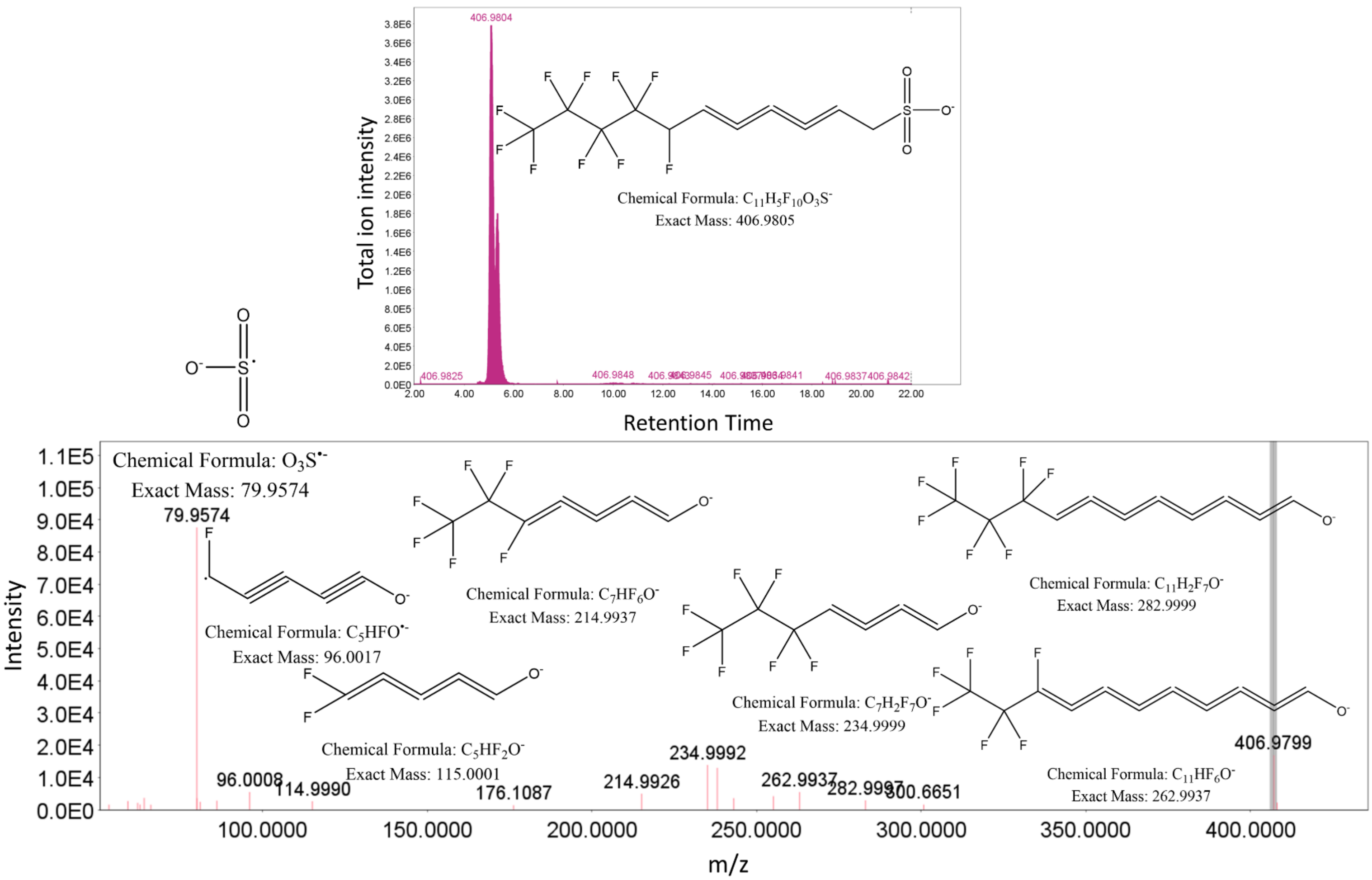

Figure S9: Chromatogram with tentative identified structure and annotated MS^2 spectrum (ESI-, mean collision energy: 77.5 V) of PID3 (m/z = 406.9805, RT = 5.1min, Confidence Level 2) in MeOH extract of T300 B.

PID4

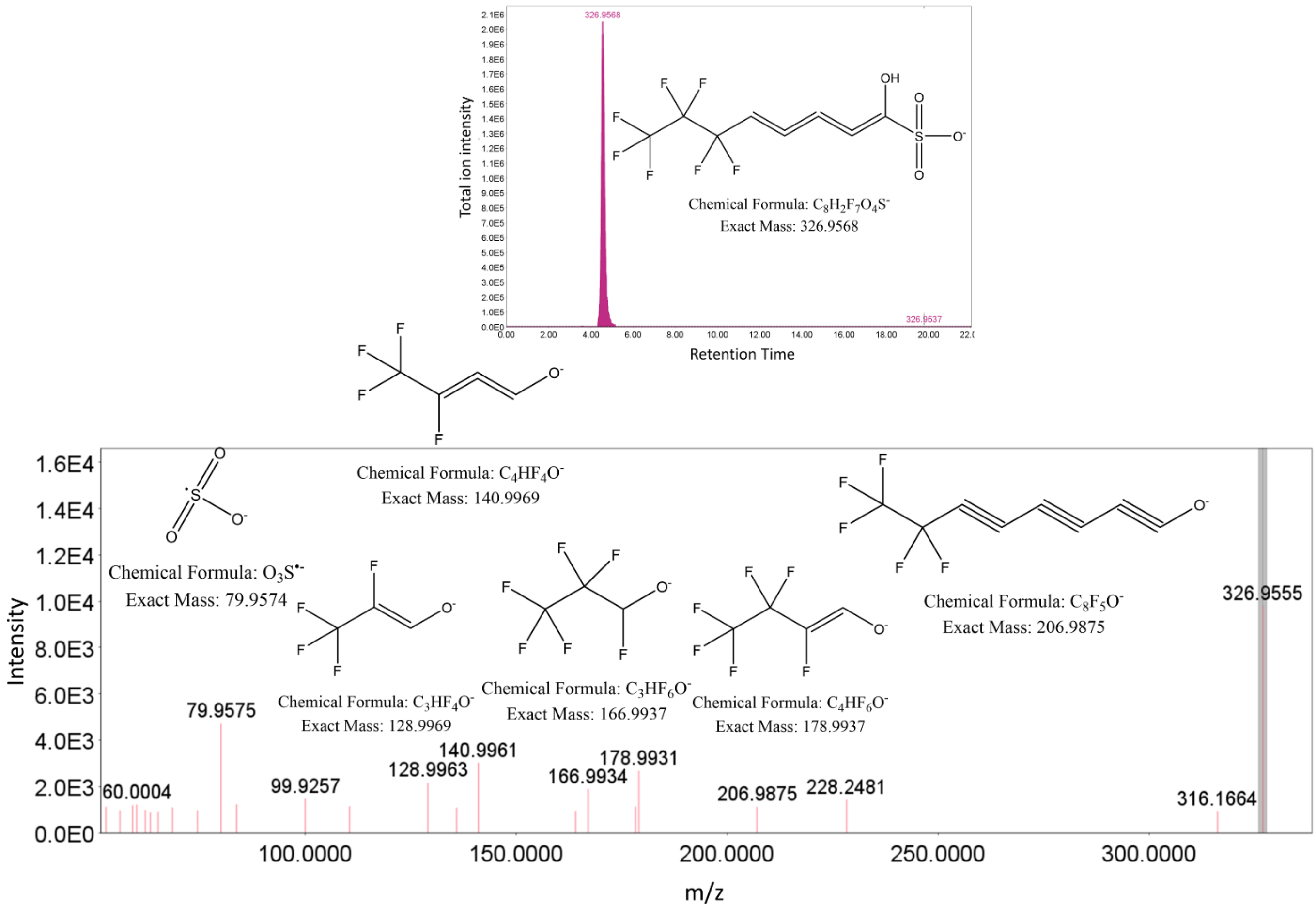

Figure S10: Chromatogram with tentative identified structure (T300 B) and annotated MS^2 spectrum (ESI-, mean collision energy: 77.5 V) of PID4 (m/z = 326.9568, RT = 4.57 min, Confidence Level 3) in MeOH extract of T300 A.

PID5

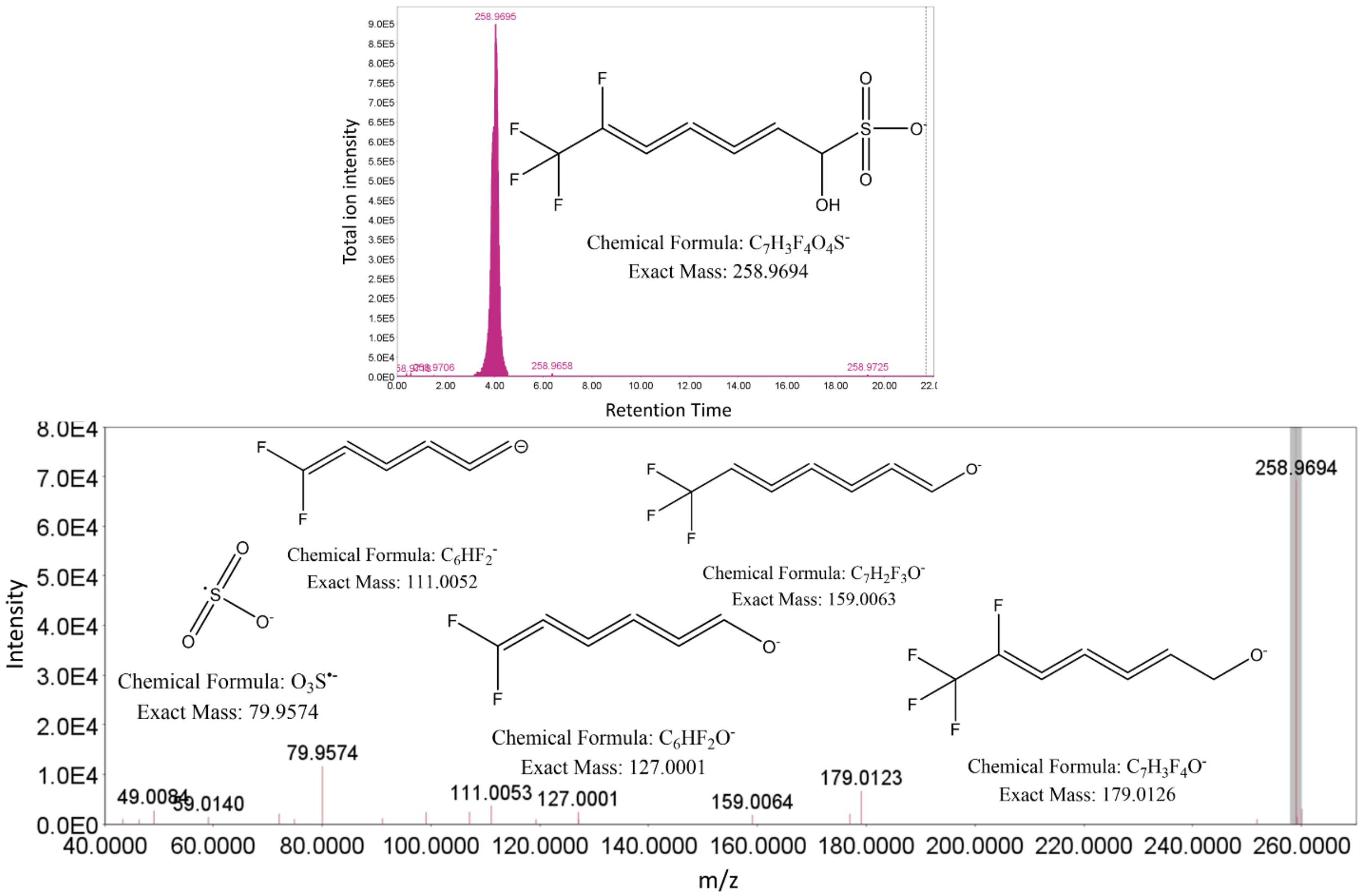

Figure S11: Chromatogram with tentative identified structure and annotated MS^2 spectrum (ESI-, mean collision energy: 77.5 V) of PID5 (m/z = 258.9694, RT = 4.1 min, Confidence Level 3) in MeOH extract of T300 B.

FTSAm derivative PIDs

PID6

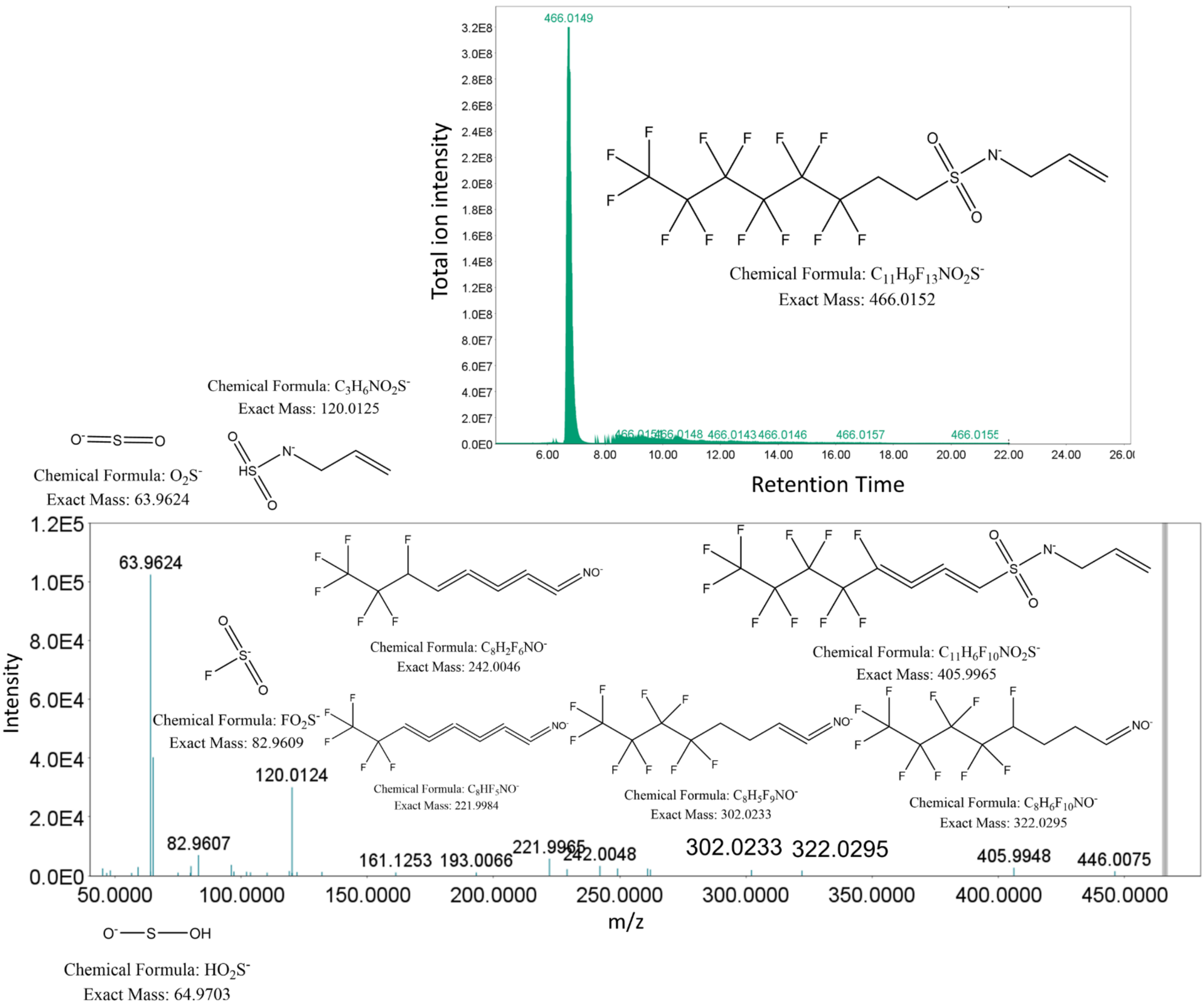

Figure S12: Chromatogram with tentative identified structure (T200B) and annotated MS^2 spectrum (ESI-, mean collision energy: 77.5 V) of PID6 (m/z = 466.0152, RT = 6.7 min, Confidence Level 2) in MeOH extract of T200 A.

PID7

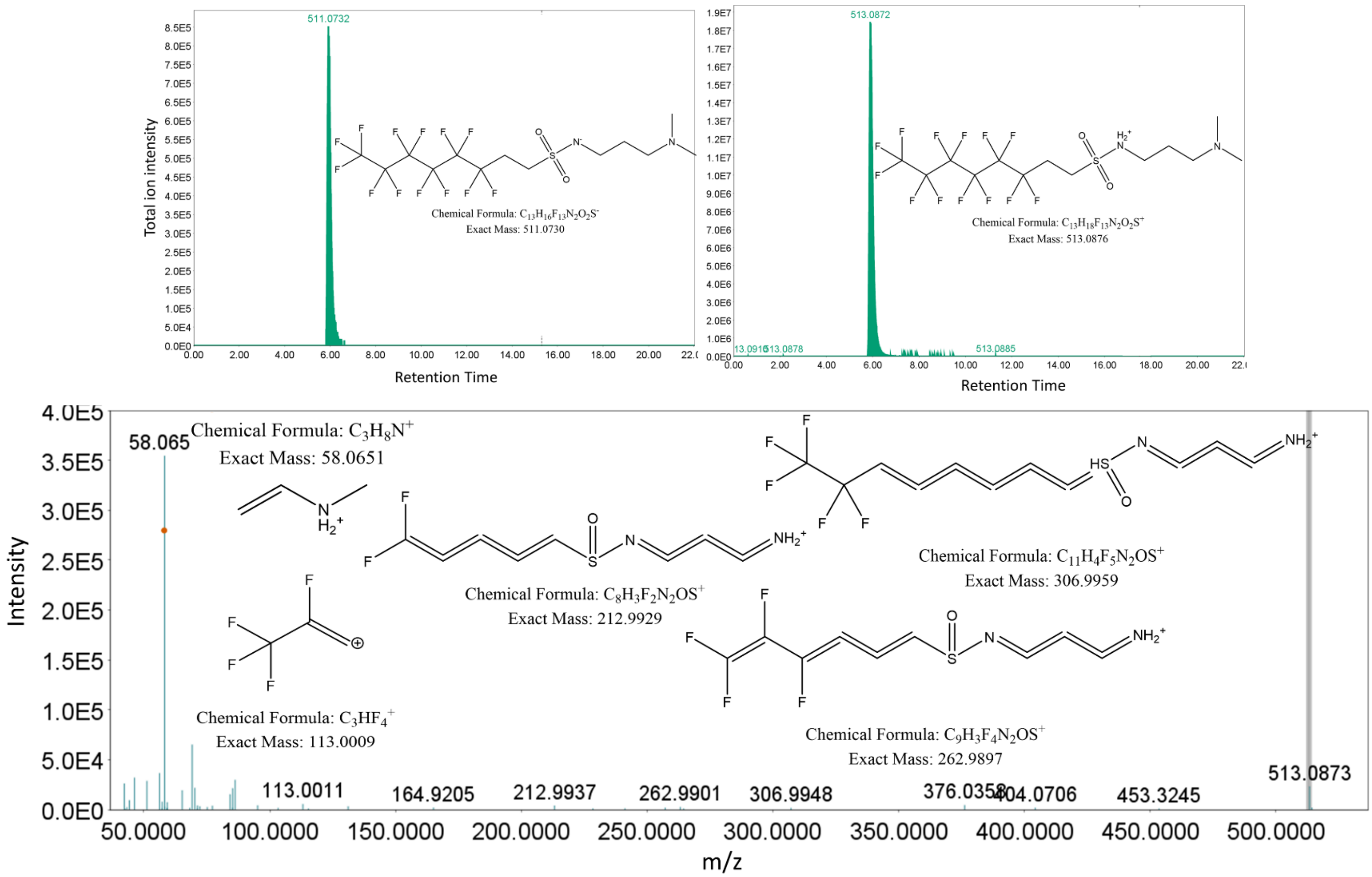

Figure S13: Chromatogram with tentative identified structure (ESI-/+) and annotated MS^2 spectrum (ESI+, mean collision energy: 77.5 V) of PID7 (m/z = 511.0730/513.0873, RT = 6.3 min, Confidence Level 2) in MeOH extract of T200 B (1:100).

PID8

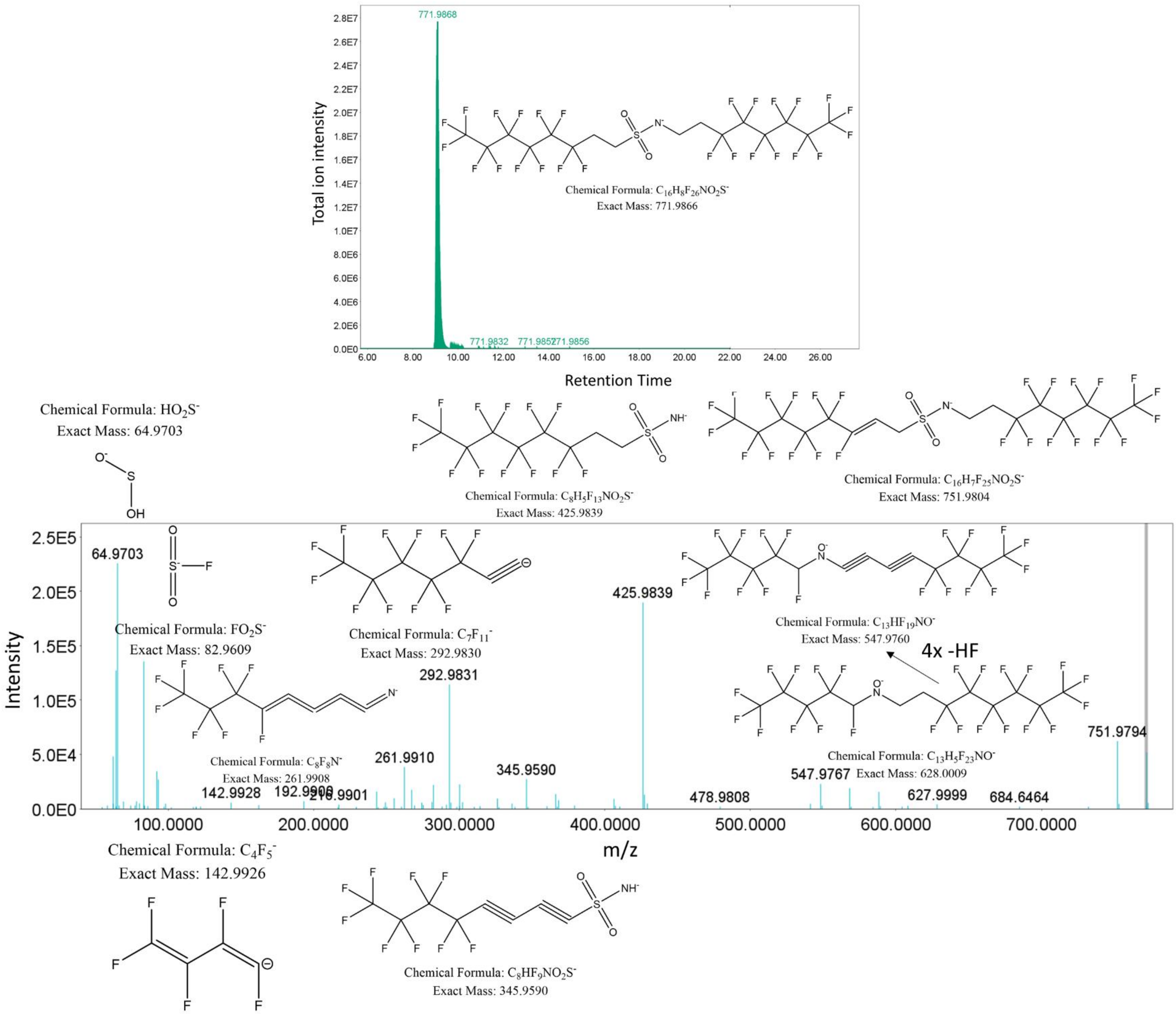

Figure S14: Chromatogram with tentative identified structure and annotated MS^2 spectrum (ESI-, mean collision energy: 77.5 V) of PID8 (m/z = 771.9866, RT = 9.1 min, Confidence Level 2) in MeOH extract of T100 B.

PID9

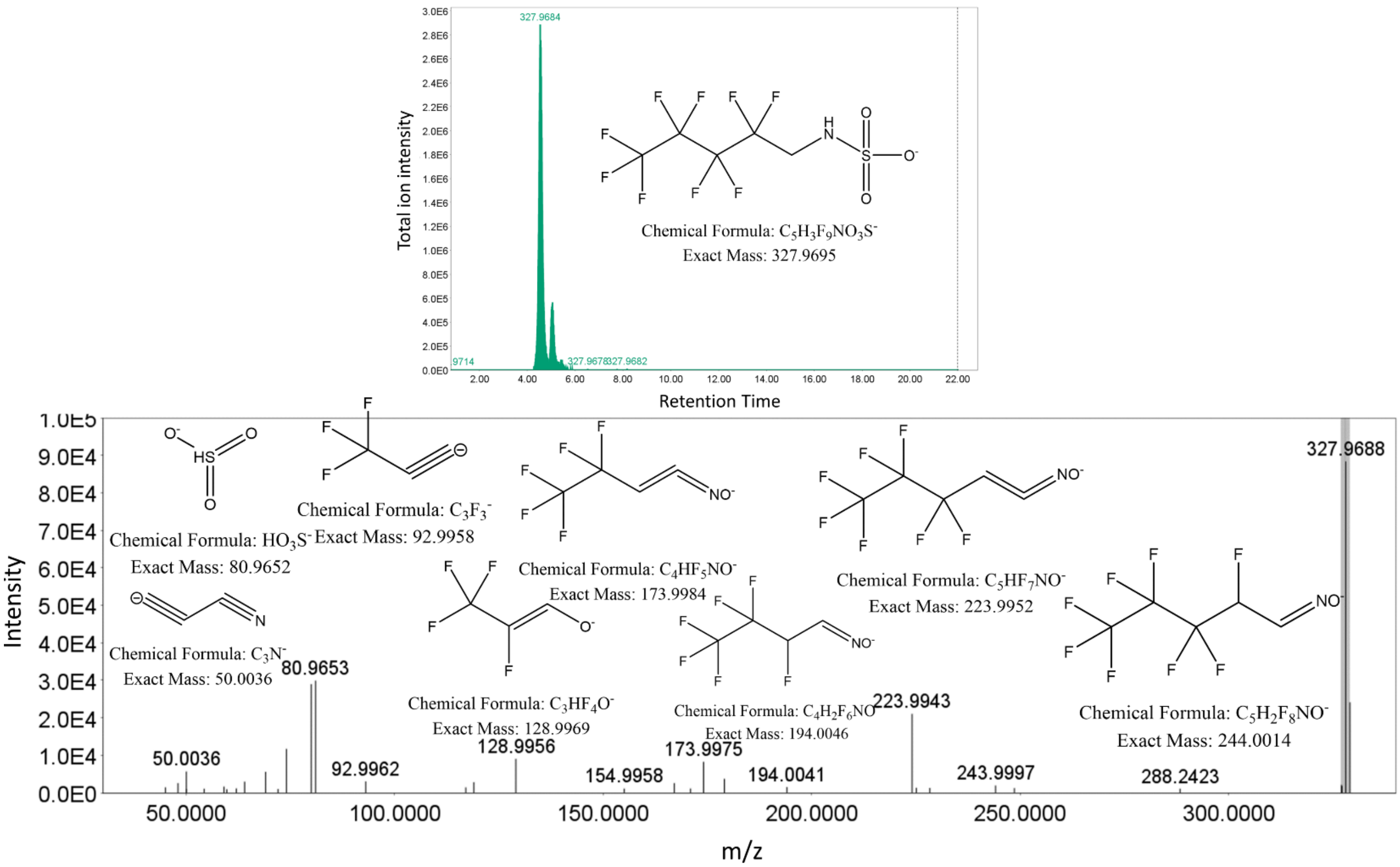

Figure S15: Chromatogram with tentative identified structure (T200 B) and annotated MS^2 spectrum (ESI-, mean collision energy: 77.5 V) of PID9 (m/z = 327.9685, RT = 4.6 min, Confidence Level 2) in MeOH extract of T200 A.

PID10

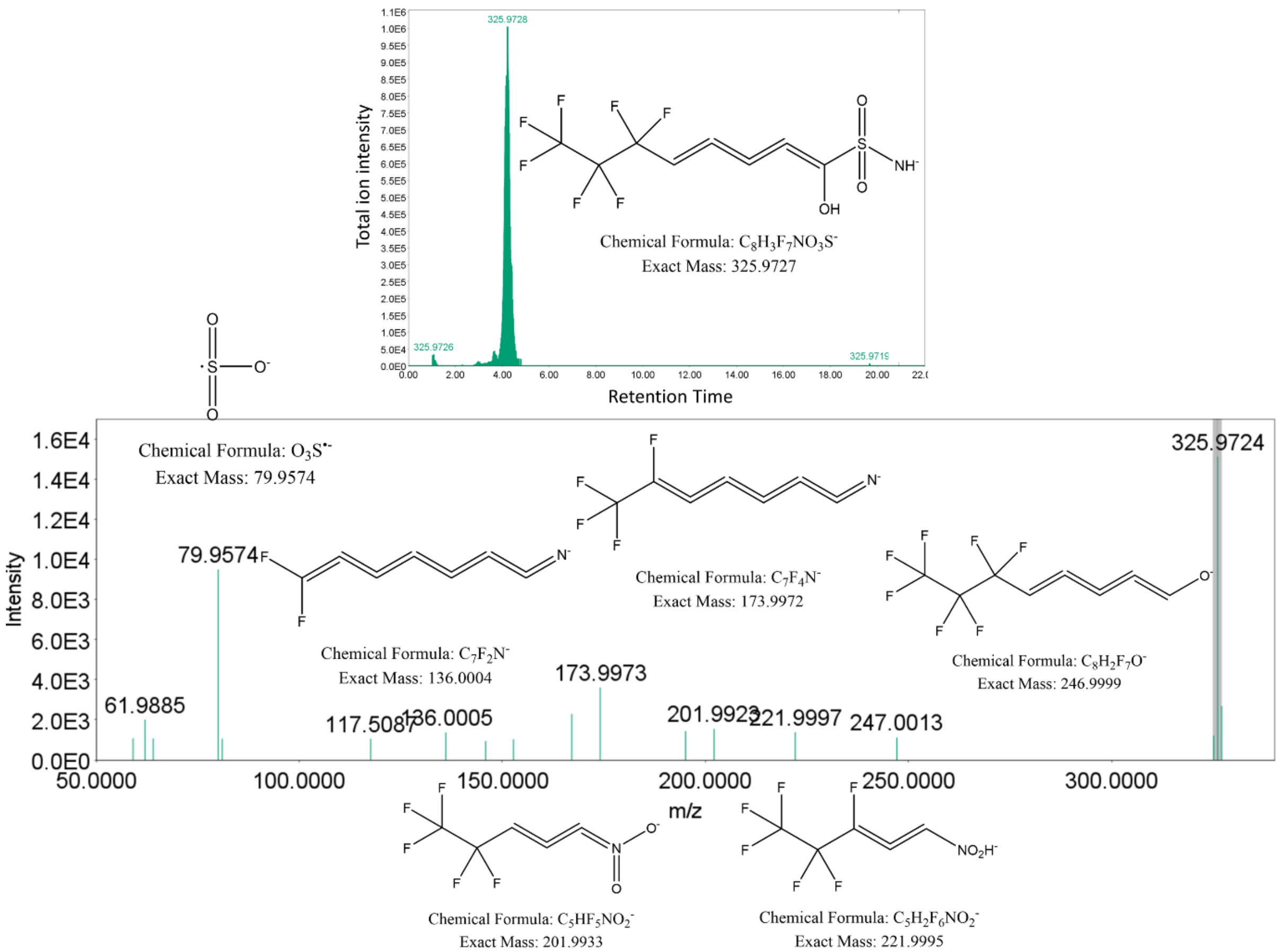

Figure S16: Chromatogram with tentative identified structure (T300 B) and annotated MS^2 spectrum (ESI-, mean collision energy: 77.5 V) of PID10 (m/z = 325.9729, RT = 4.24 min, Confidence Level 3) in MeOH extract of T300 A.

PID11

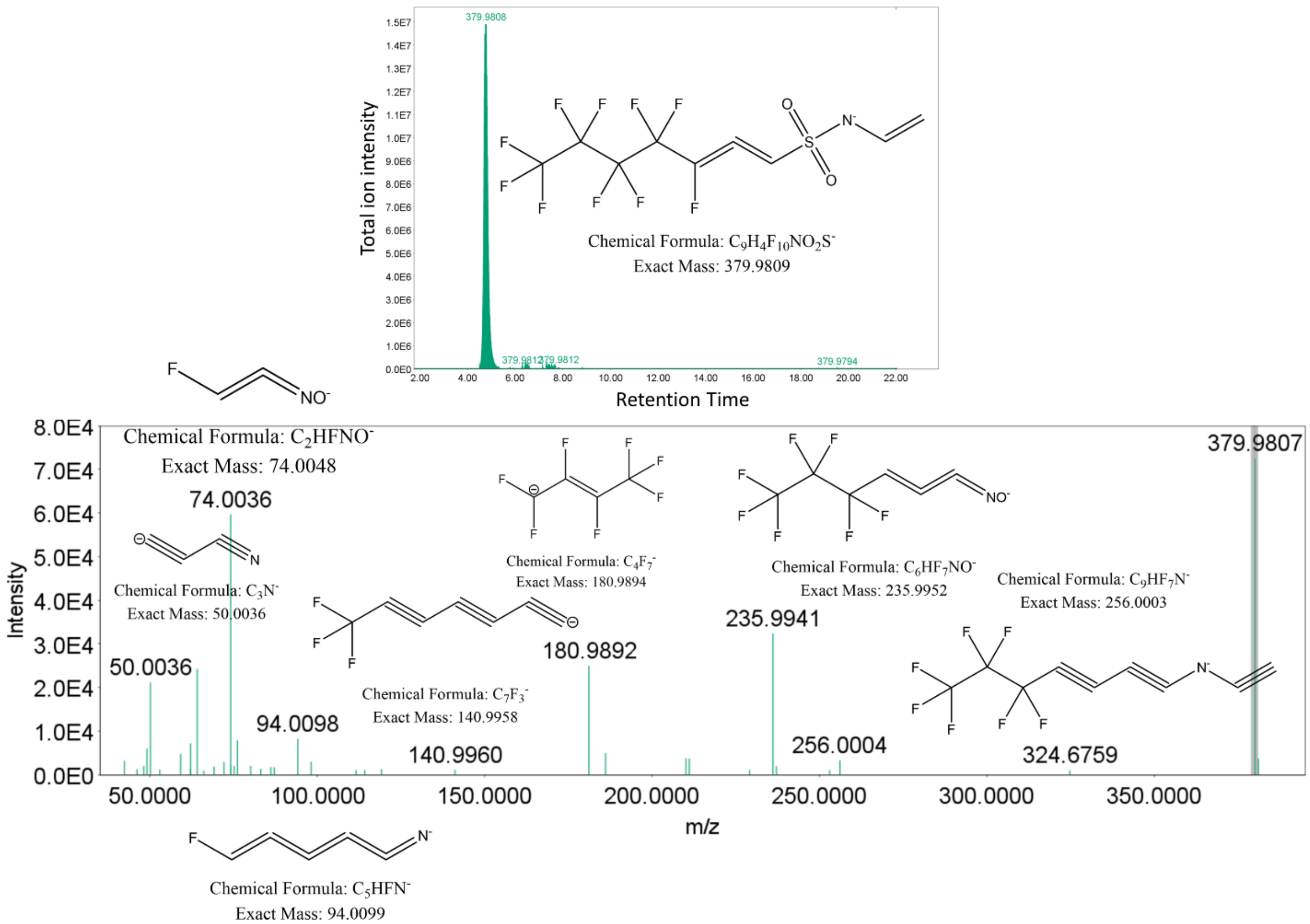

Figure S17: Chromatogram with tentative identified structure and annotated MS^2 spectrum (ESI-, mean collision energy: 77.5 V) of PID11 (m/z = 379.9807, RT = 4.8 min, Confidence Level 3) in MeOH extract of T200 B.

PID12

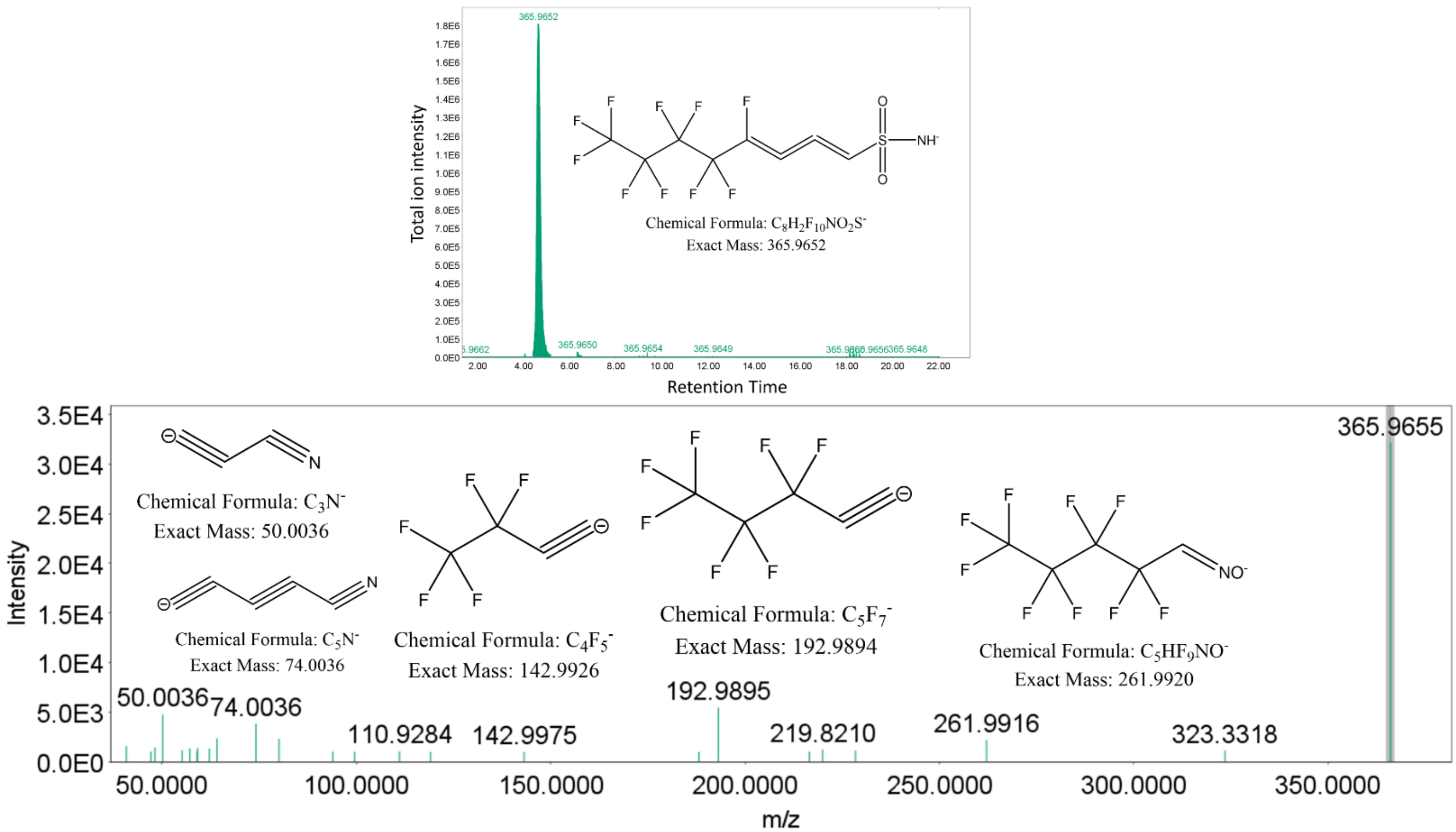

Figure S18: Chromatogram with tentative identified structure (T200 B) and annotated MS^2 spectrum (ESI-, mean collision energy: 77.5 V) of PID17 (m/z = 365.9651, RT = 4.64 min, Confidence Level 2) in MeOH extract of T200 A.

Hydroxy carboxylic acid derivative PIDs

PID13

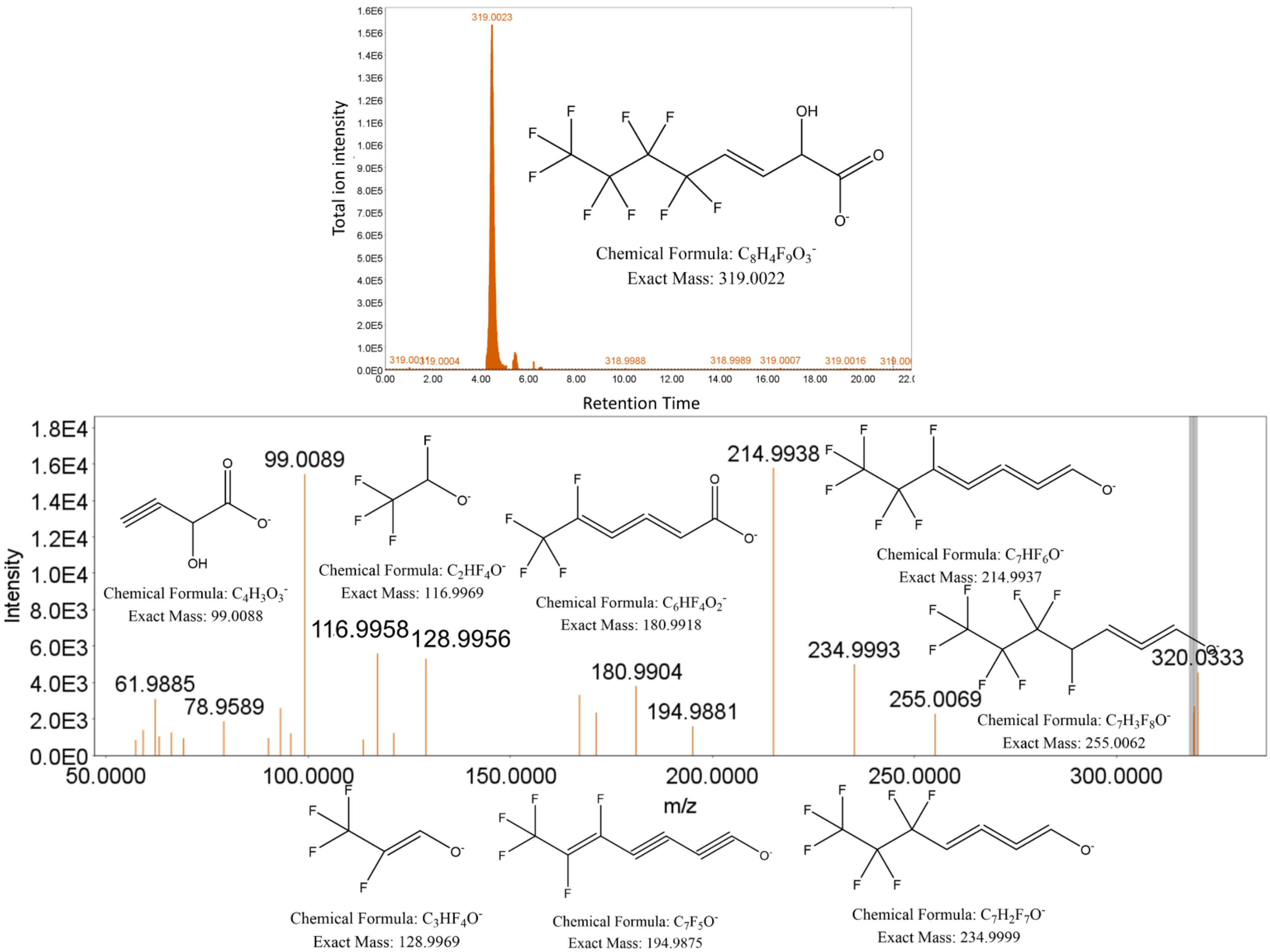

Figure S19: Chromatogram with tentative identified structures and annotated MS^2 spectrum (ESI-, mean collision energy: 77.5 V) of PID13 (m/z = 319.0023, RT = 4.5 min, Confidence Level 3) in MeOH extract of T200 B.

PID14

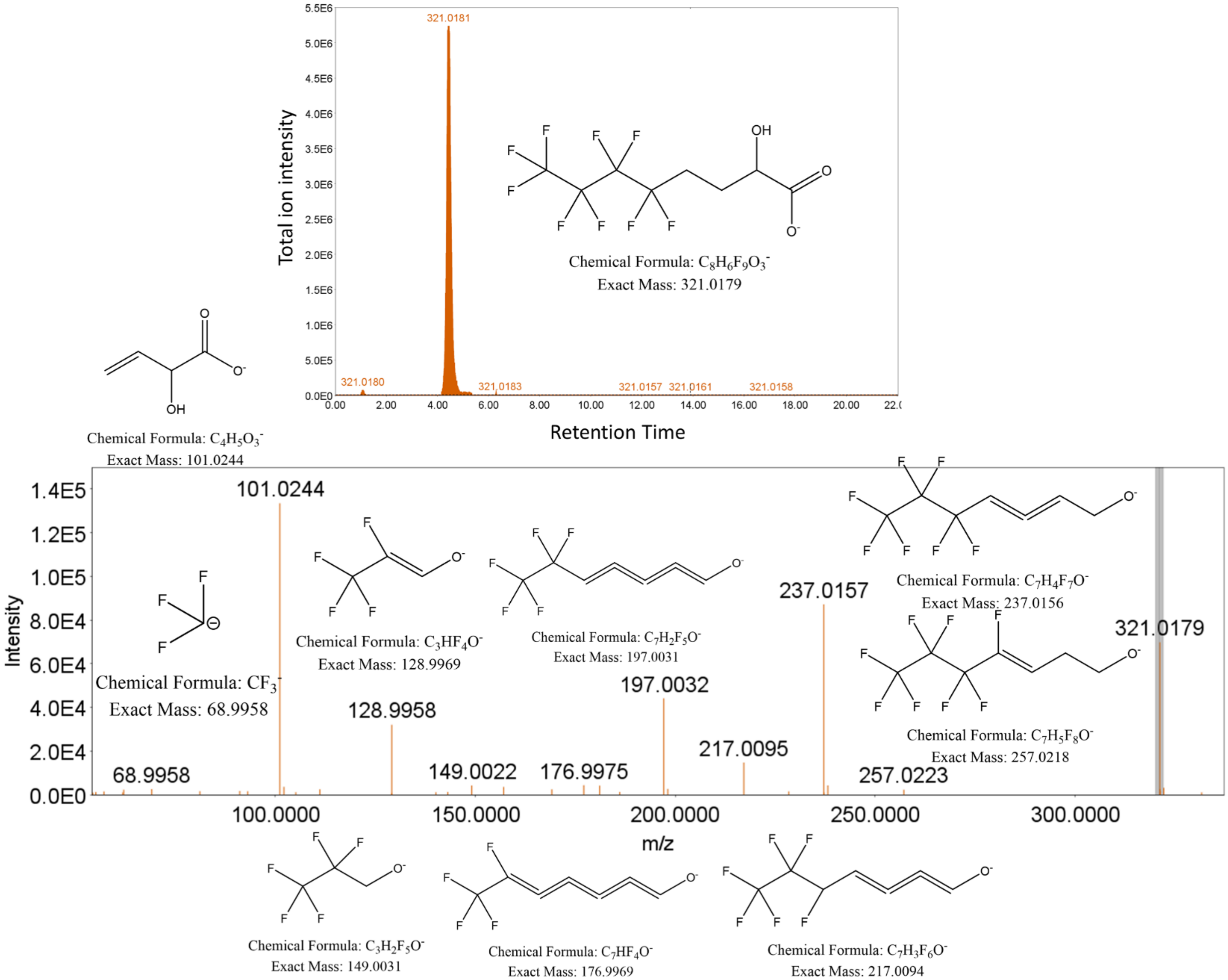

Figure S20: Chromatogram with tentative identified structures and annotated MS^2 spectrum (ESI-, mean collision energy: 77.5 V) of PID14 (m/z = 321.0181, RT = 4.4 min, Confidence Level 3) in MeOH extract of T200 B.

Unknown polyfluoro-hydroxyalkyl derivative PIDs (PFOHs)

HS3a

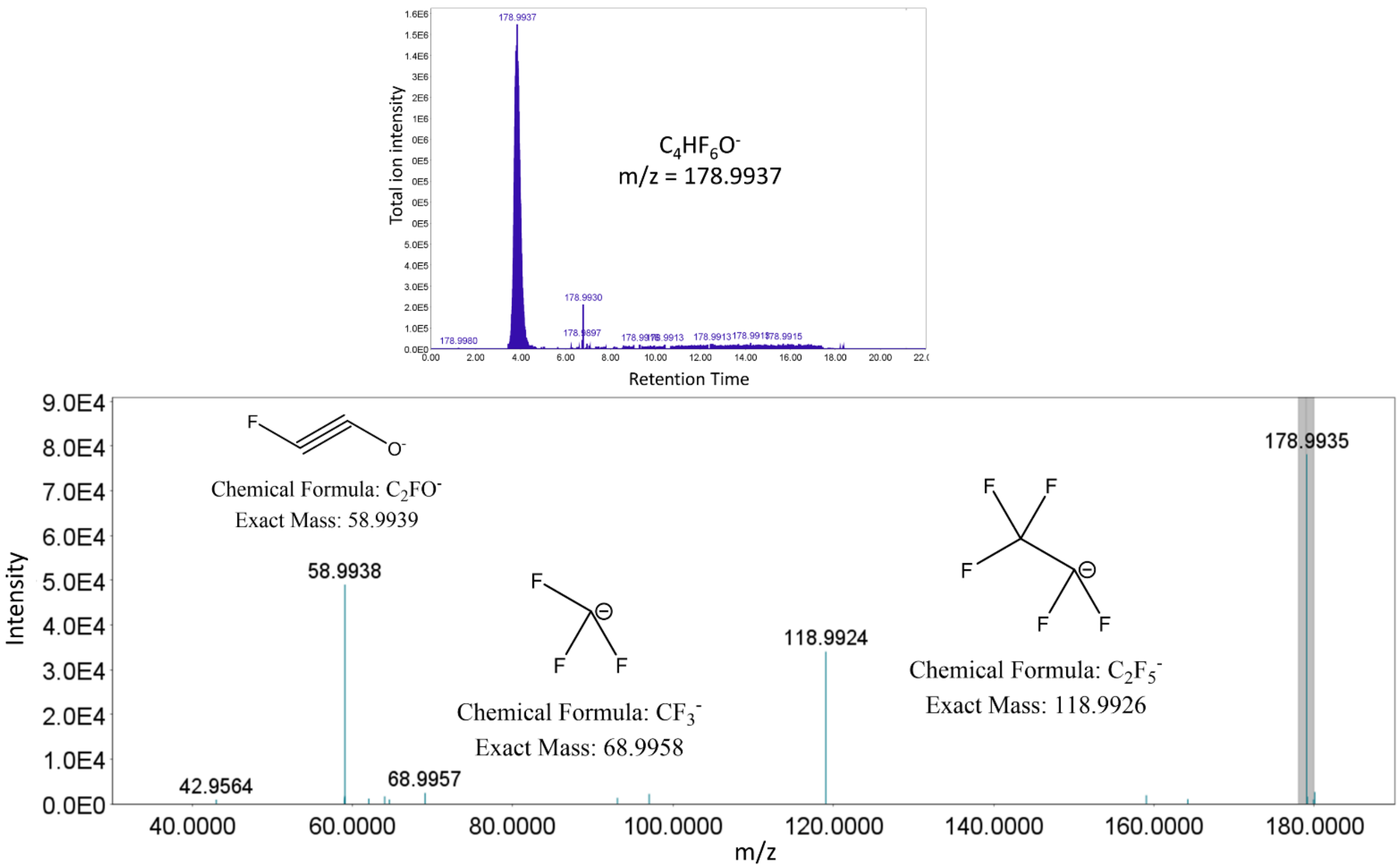

Figure S21: Chromatogram with proposed chemical formula and annotated MS^2 spectrum (ESI-, mean collision energy: 77.5 V) of HS3a (m/z = 178.9937, RT = 3.8 min, Confidence Level 4) in MeOH extract of T200 B.

HS3b

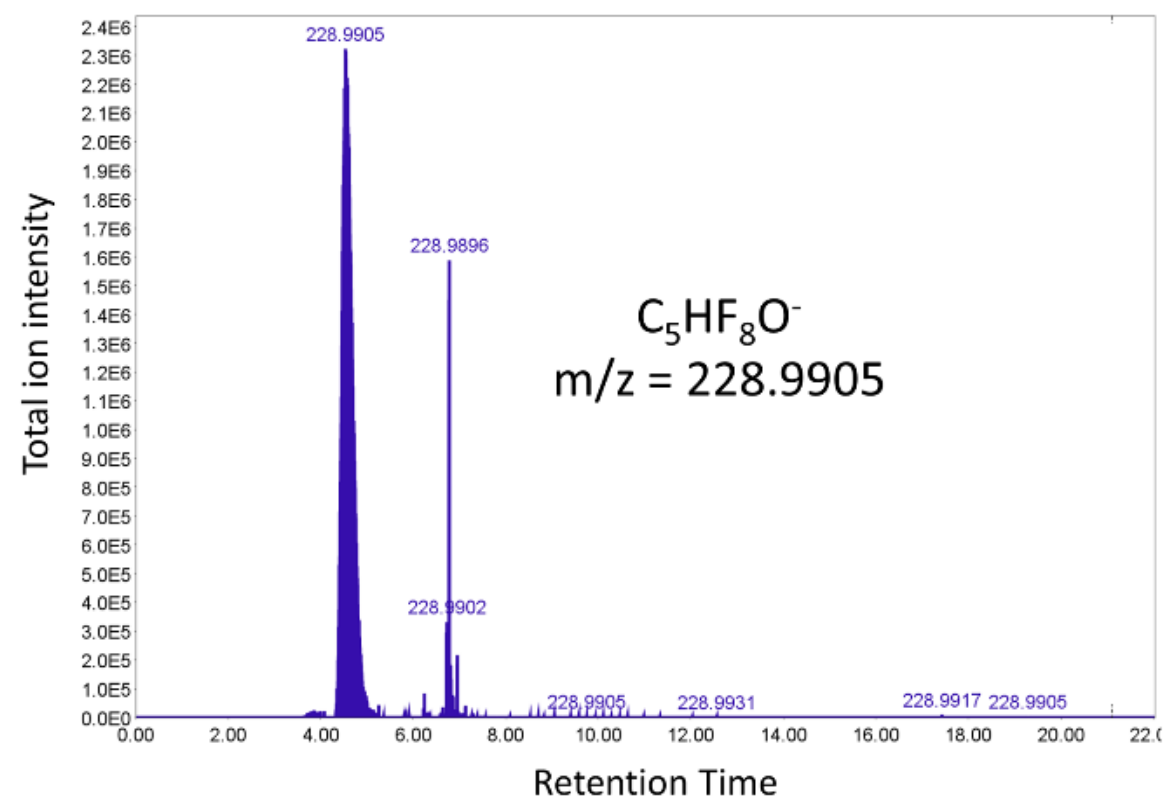

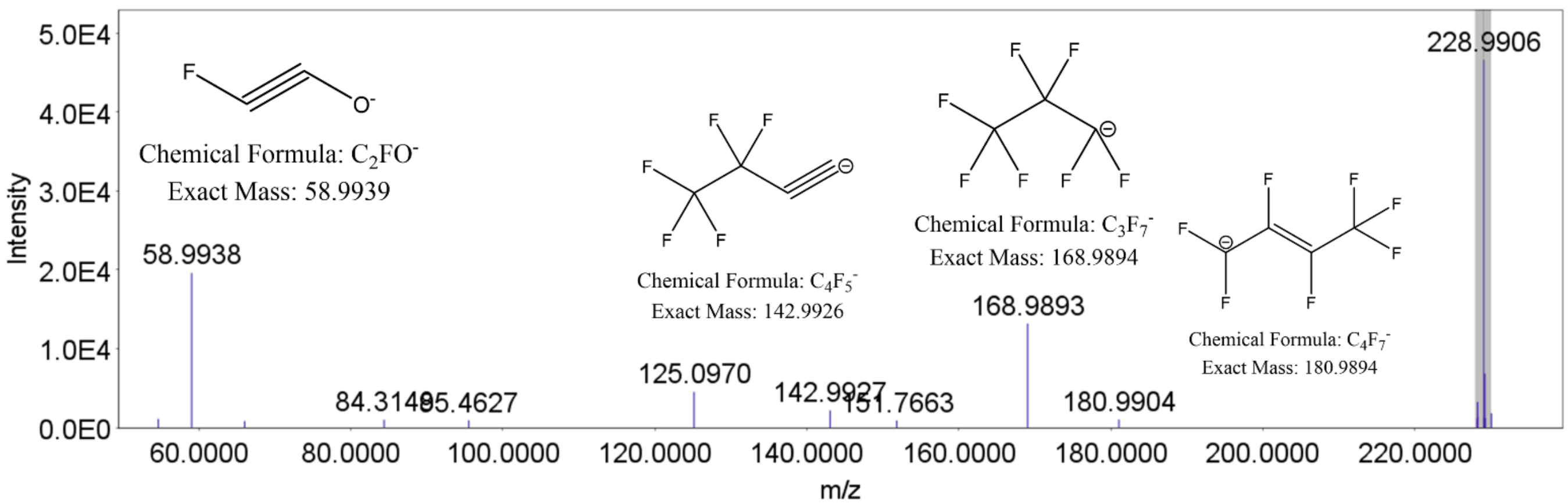

Figure S22: Chromatogram with proposed chemical formula and annotated MS^2 spectrum (ESI-, mean collision energy: 77.5 V) of HS3b (m/z = 228.9905, RT = 4.5 min, Confidence Level 4) in MeOH extract of T200 B.

HS3c

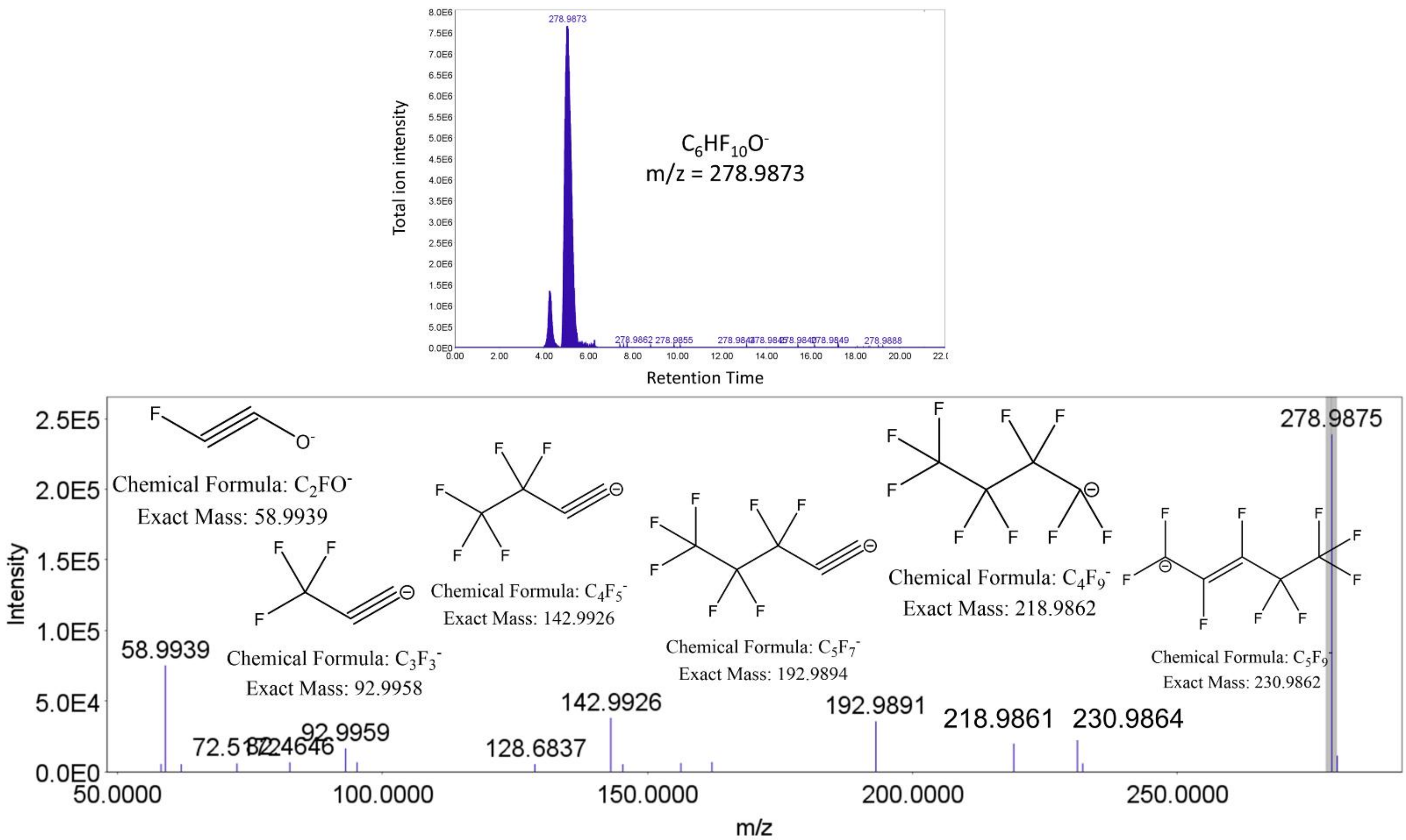

Figure S23: Chromatogram with proposed chemical formula and annotated MS^2 spectrum (ESI-, mean collision energy: 77.5 V) of HS3c (m/z = 278.9874, RT = 5.1 min, Confidence Level 4) in MeOH extract of T200 B.

HS4a

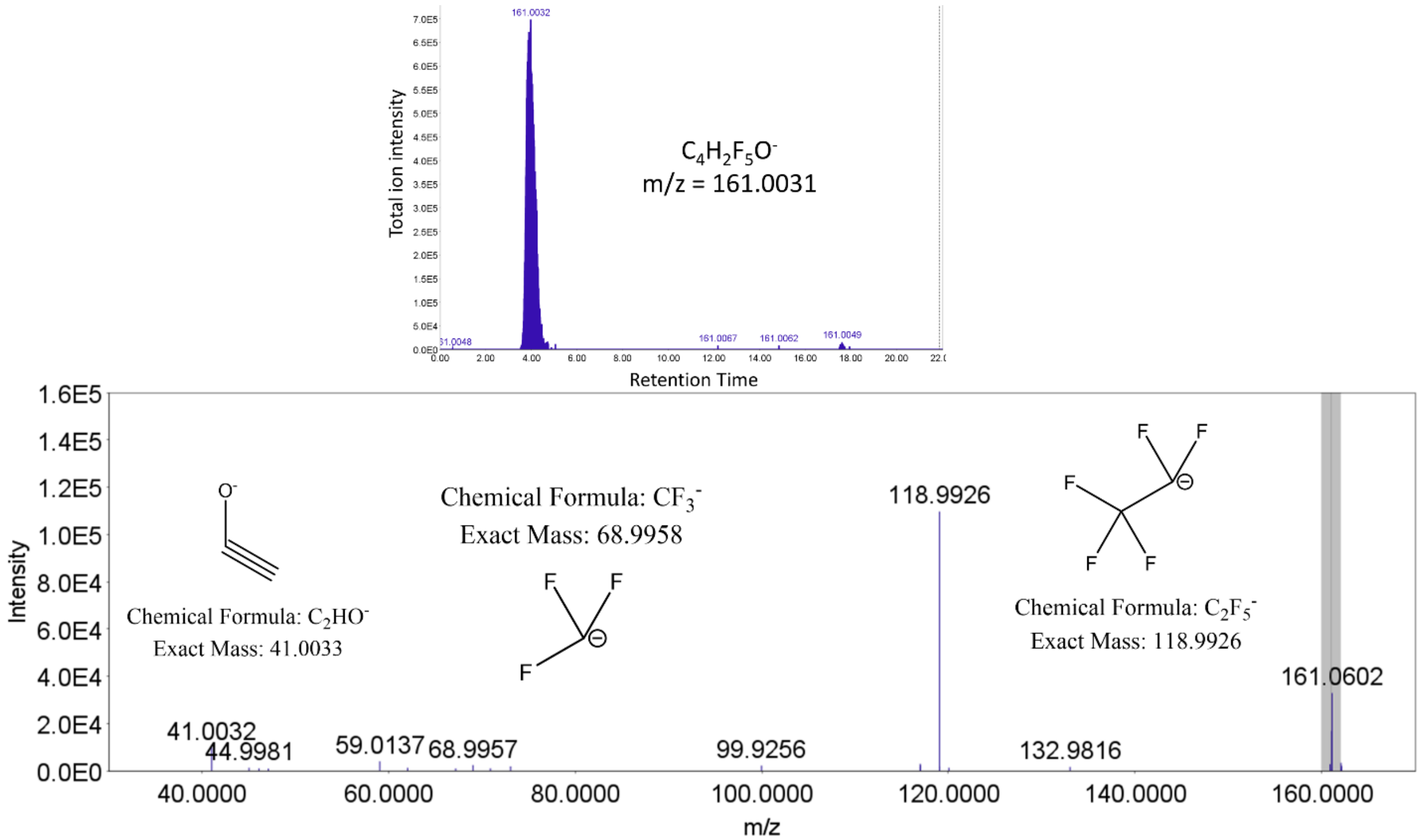

Figure S24: Chromatogram with proposed chemical formula and annotated MS[2] spectrum (ESI-, mean collision energy: 77.5 V) of HS4a (m/z = 161.0032, RT = 3.9 min, Confidence Level 4) in MeOH extract of T300 B.

HS4b

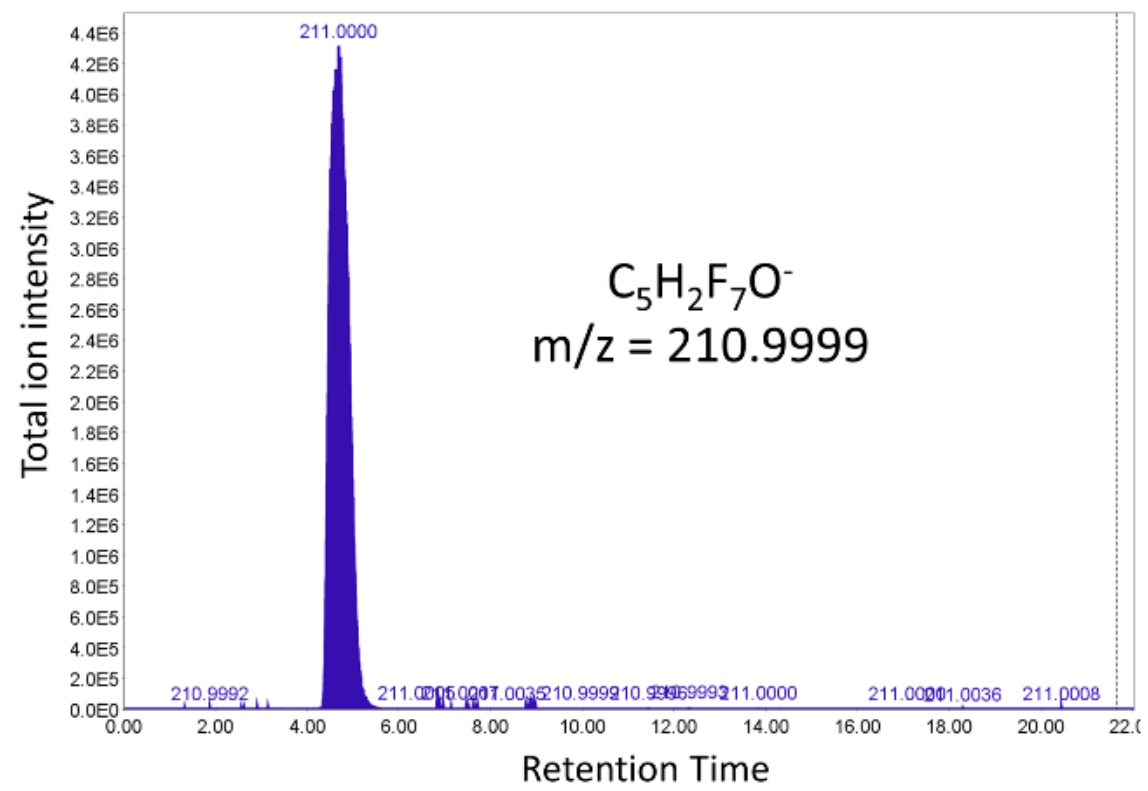

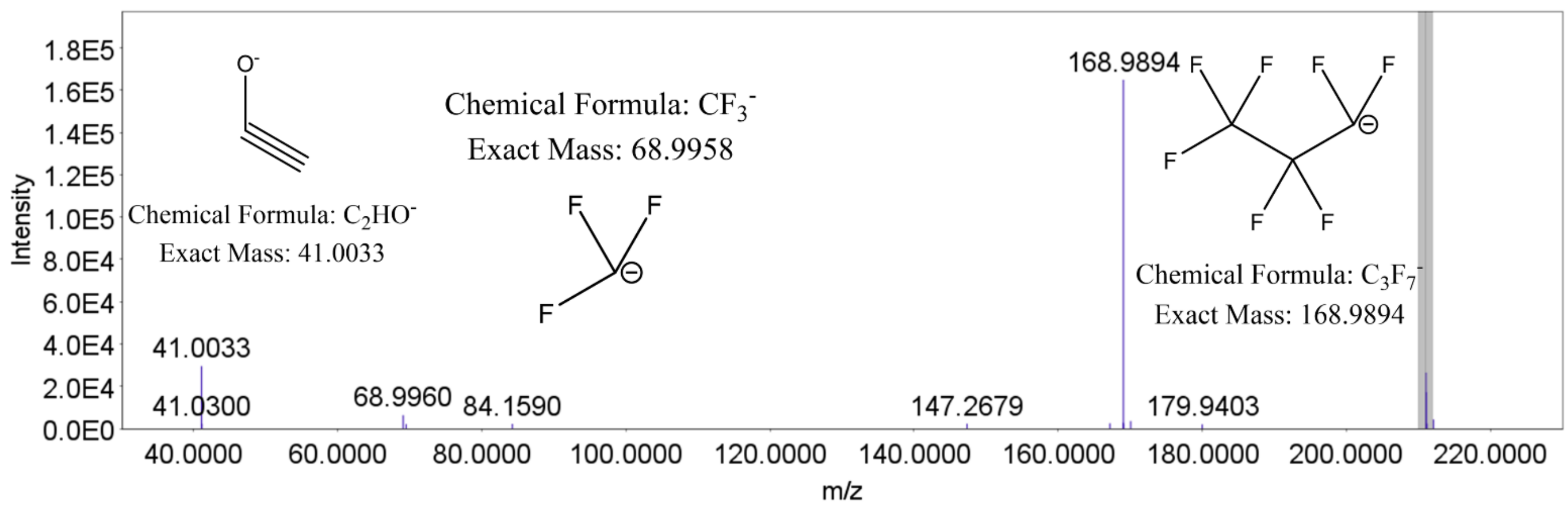

Figure S25: Chromatogram with proposed chemical formula and annotated MS^2 spectrum (ESI-, mean collision energy: 77.5 V) of HS4b (m/z = 210.9999, RT = 4.7 min, Confidence Level 4) in MeOH extract of T300 B.

HS4c

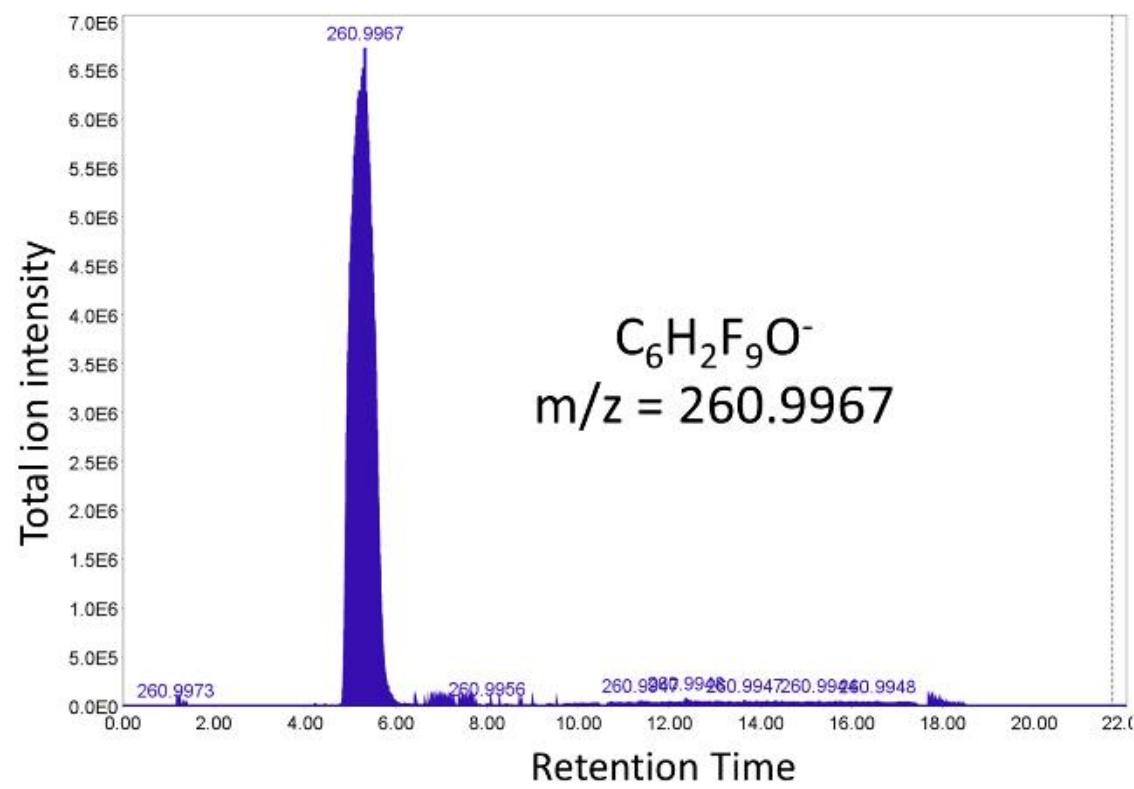

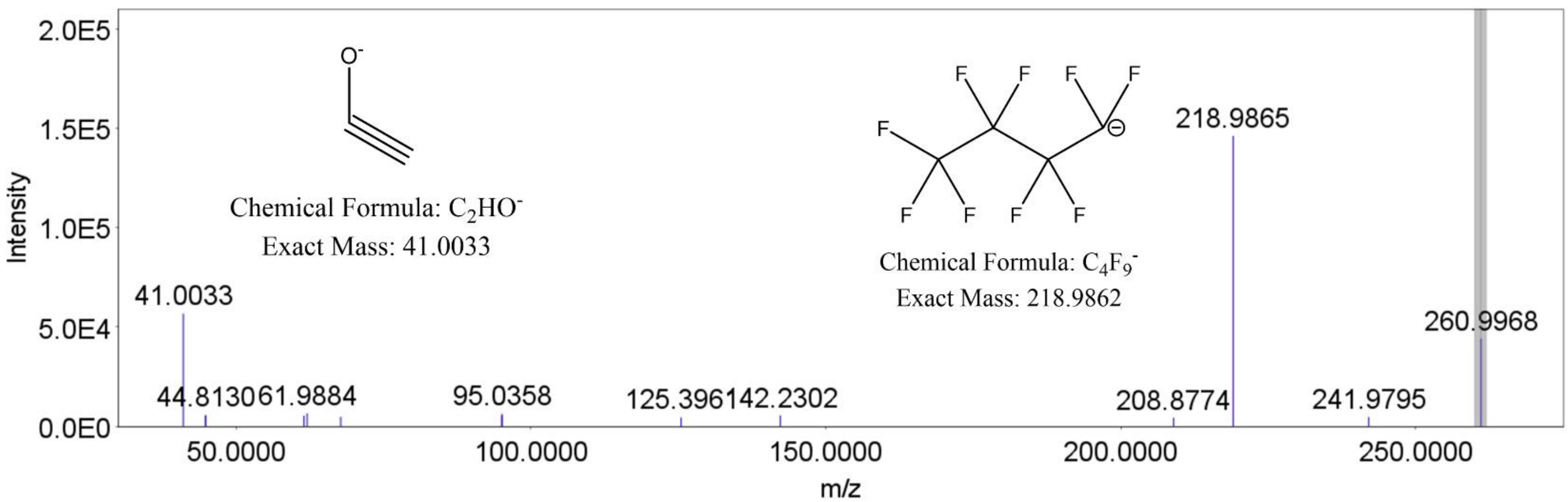

Figure S26: Chromatogram with proposed chemical formula and annotated MS^2 spectrum (ESI-, mean collision energy: 77.5 V) of HS4c (m/z = 260.9968, RT = 5.3 min, Confidence Level 4) in MeOH extract of T300 B.

HS4d

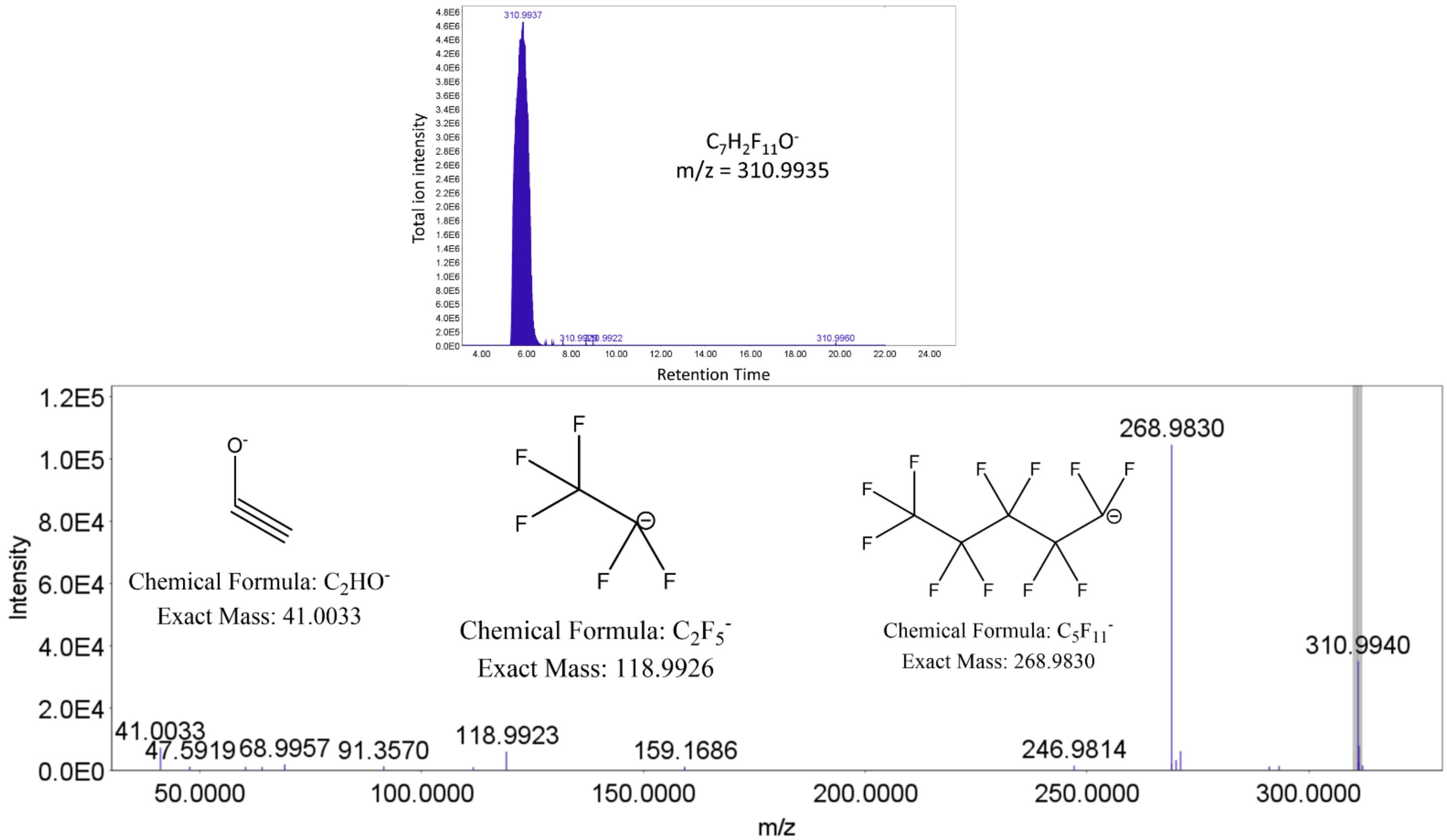

Figure S27: Chromatogram with proposed chemical formula and annotated MS^2 spectrum (ESI-, mean collision energy: 77.5 V) of HS4d (m/z = 310.9936, RT = 5.8 min, Confidence Level 4) in MeOH extract of T300 B.

HS5a

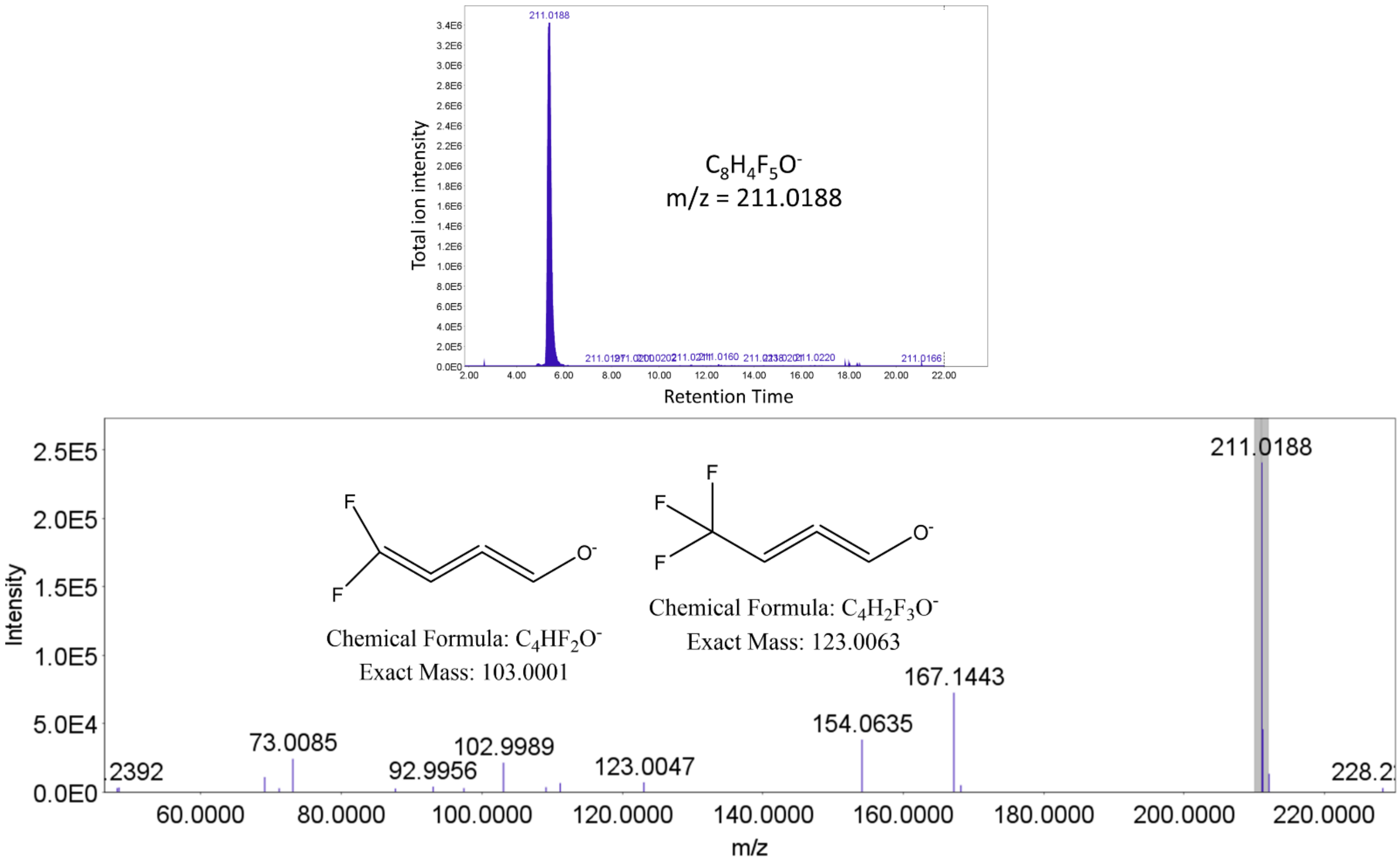

Figure S28: Chromatogram with proposed chemical formula and annotated MS^2 spectrum (ESI-, mean collision energy: 77.5 V) of HS5a (m/z = 211.0188, RT = 5.4 min, Confidence Level 4) in MeOH extract of T400 B.

HS5b

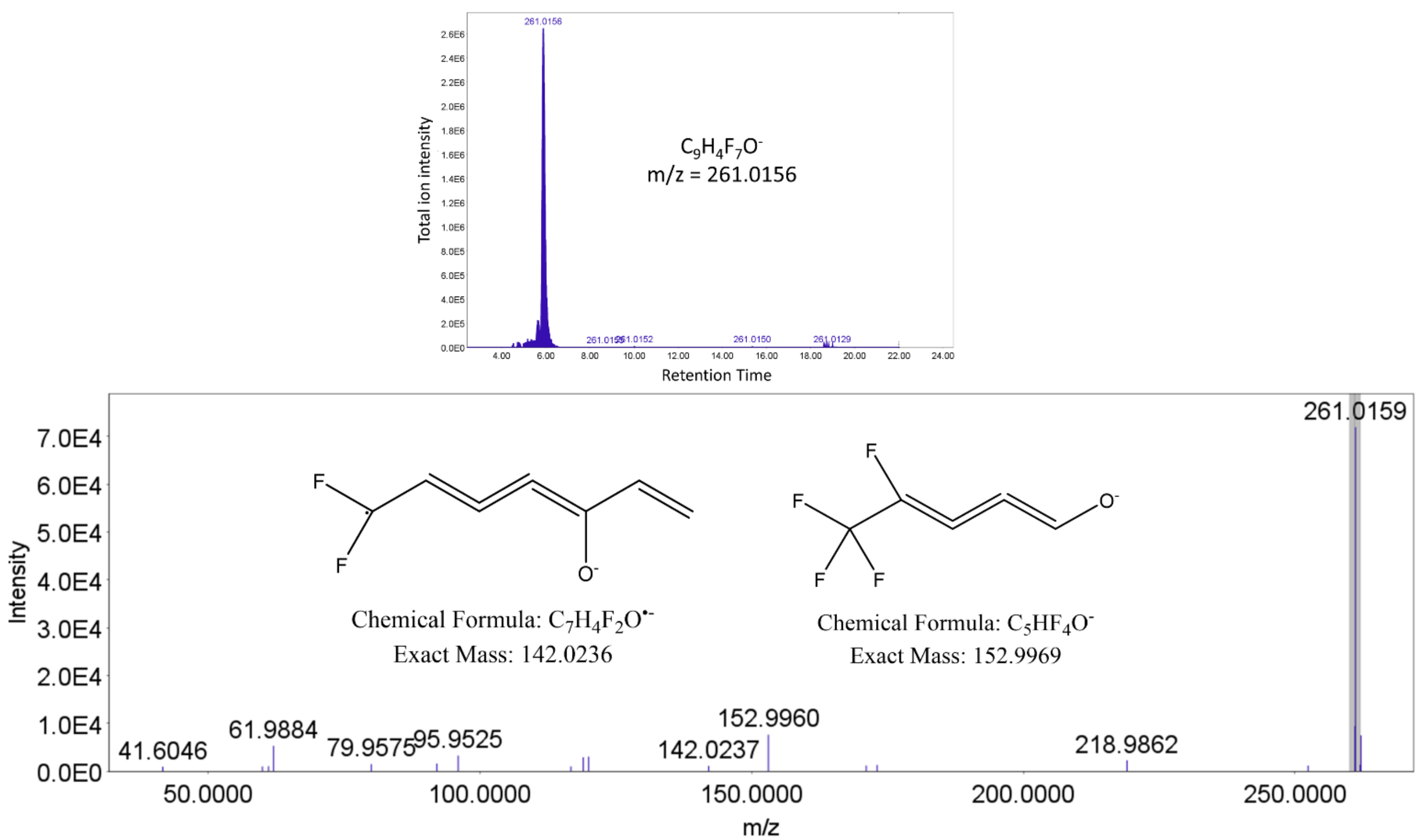

Figure S29: Chromatogram with proposed chemical formula and annotated MS^2 spectrum (ESI-, mean collision energy: 77.5 V) of HS5b (m/z = 261.0156, RT = 5.9 min, Confidence Level 4) in MeOH extract of T300 B.

HS5c

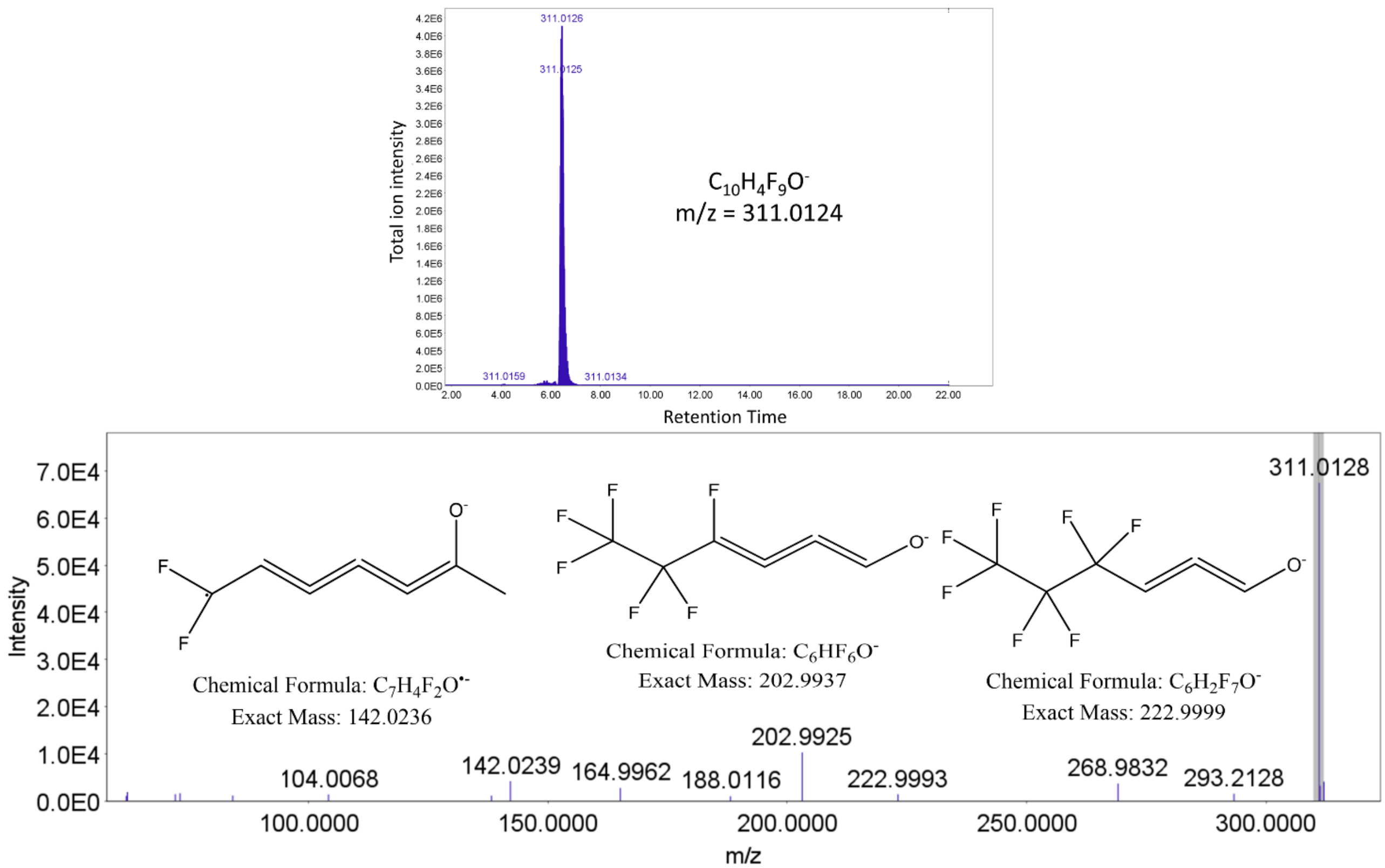

Figure S30: Chromatogram with proposed chemical formula and annotated MS^2 spectrum (ESI-, mean collision energy: 77.5 V) of HS5c (m/z = 311.0124, RT = 6.4 min, Confidence Level 4) in MeOH extract of T300 B.

HS5d

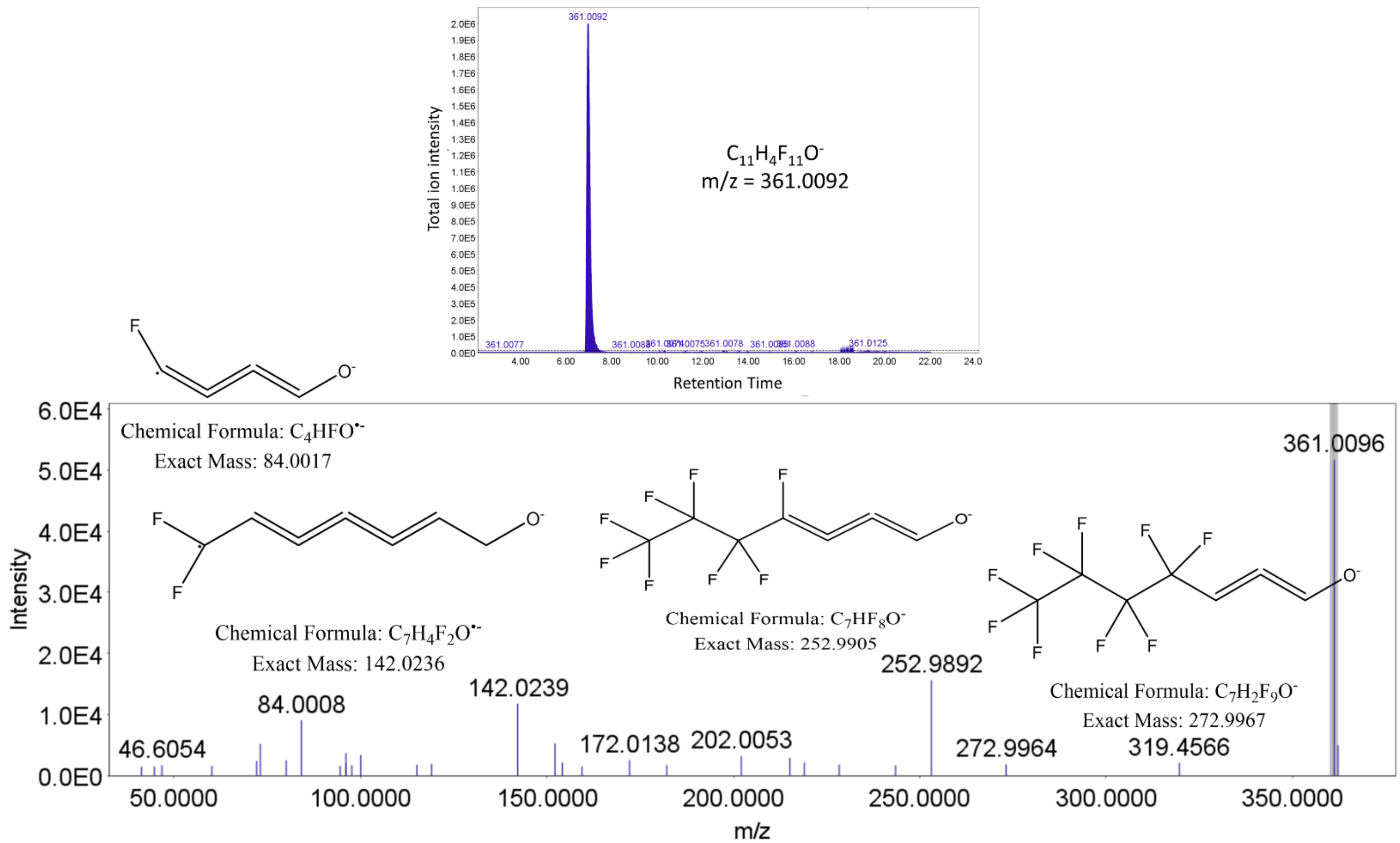

Figure S31: Chromatogram with proposed chemical formula and annotated MS^2 spectrum (ESI-, mean collision energy: 77.5 V) of HS5d (m/z = 361.0092, RT = 7.0 min, Confidence Level 4) in MeOH extract of T300 B.

HS6a

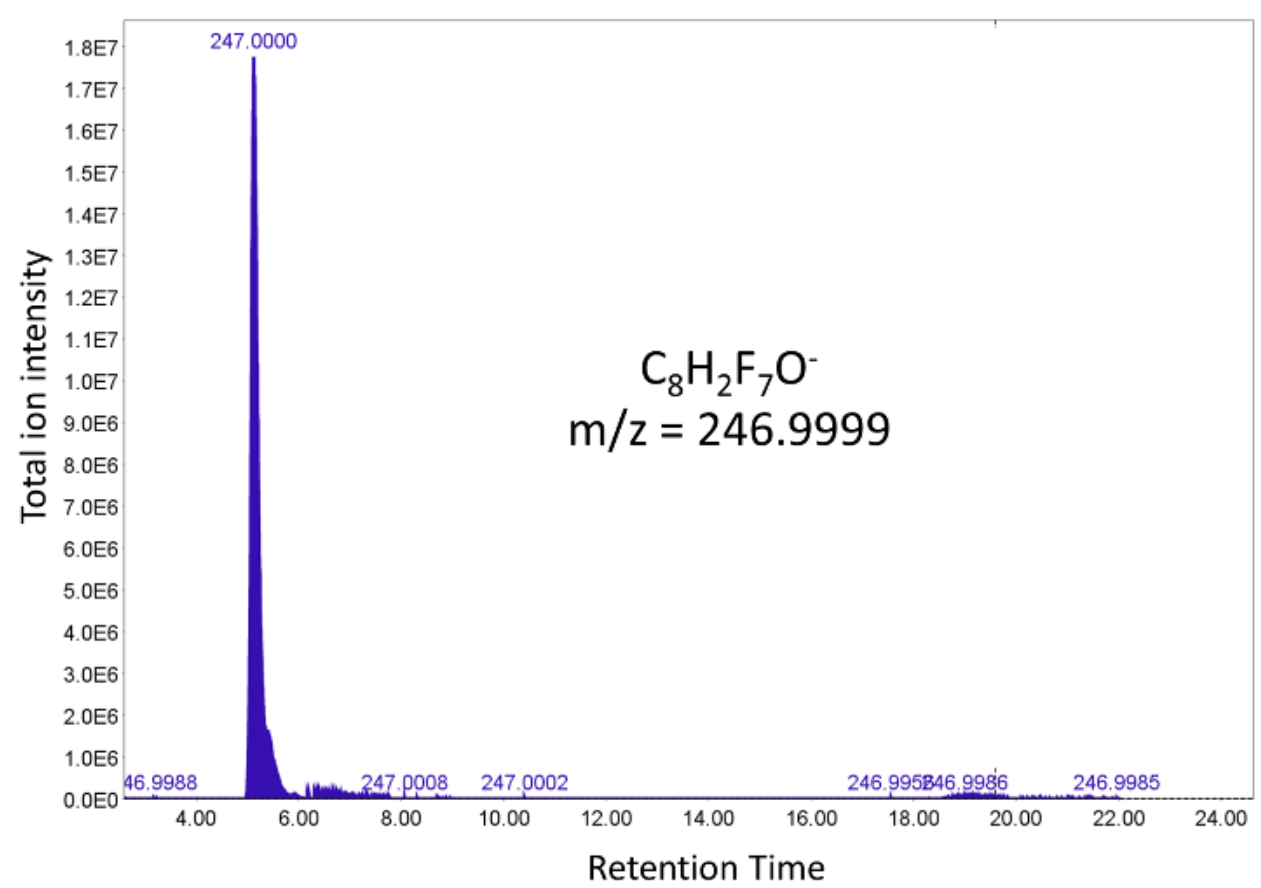

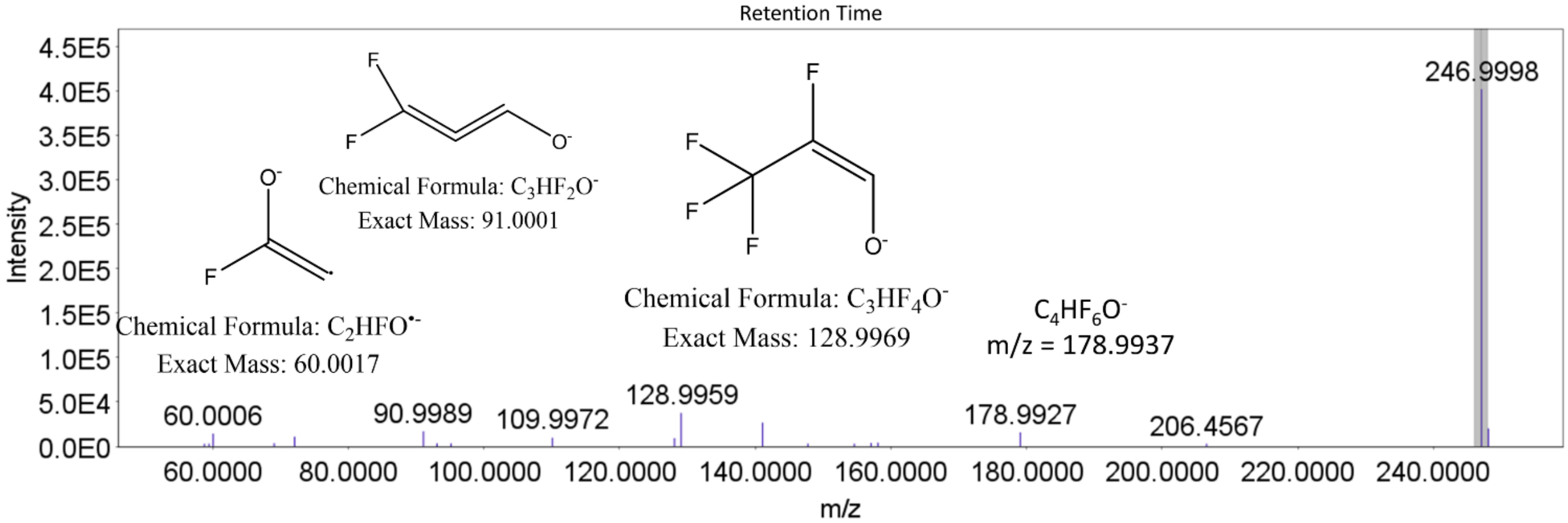

Figure S32: Chromatogram with proposed chemical formula and annotated MS^2 spectrum (ESI-, mean collision energy: 77.5 V) of HS6a (m/z = 247.0000, RT = 5.1 min, Confidence Level 4) in MeOH extract of T300 B.

HS6b

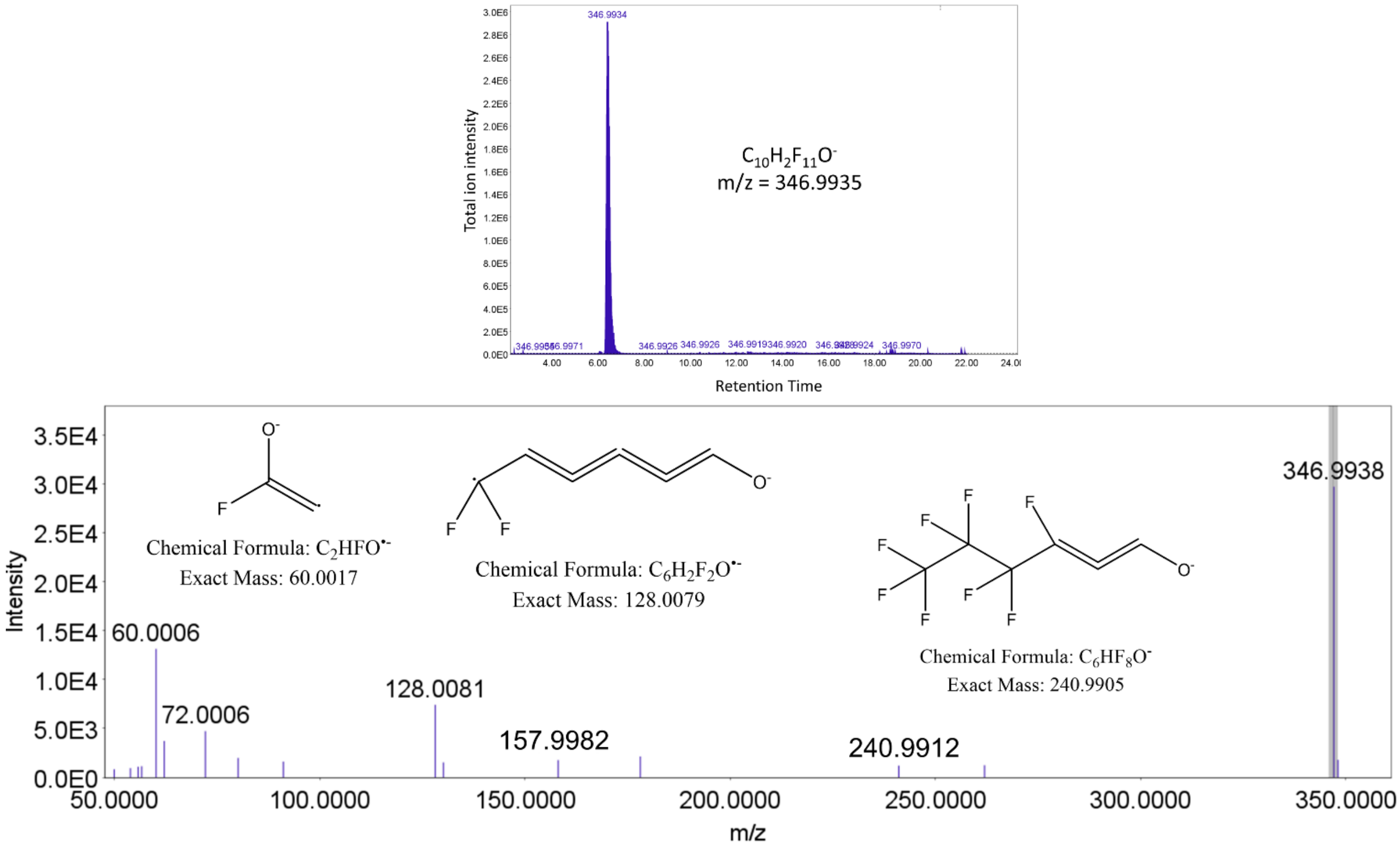

Figure S33: Chromatogram with proposed chemical formula and annotated MS^2 spectrum (ESI-, mean collision energy: 77.5 V) of HS6b (m/z = 346.9935, RT = 6.4 min, Confidence Level 4) in MeOH extract of T300 B.

Other PIDs

HS7a

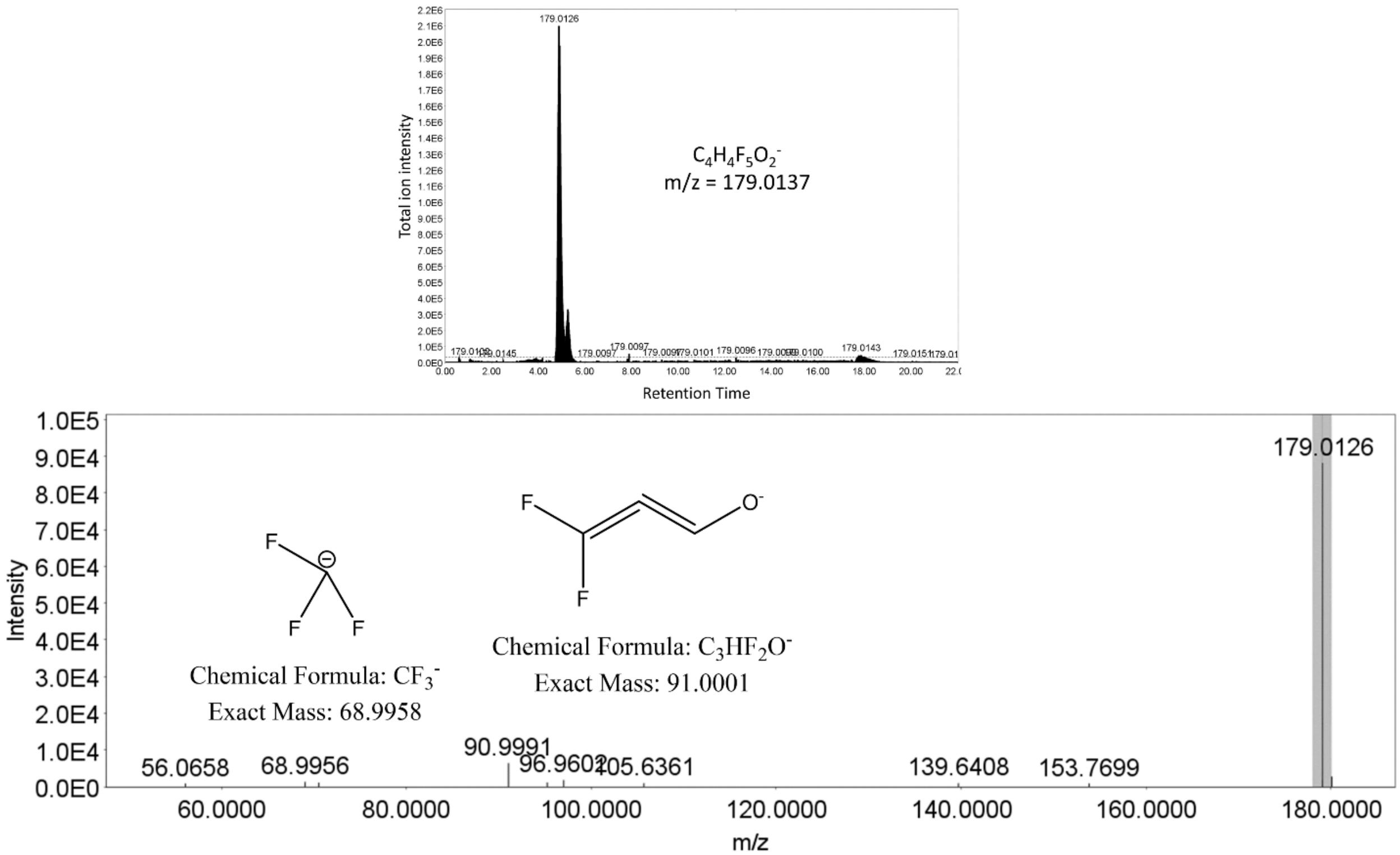

Figure S34: Chromatogram with proposed chemical formula and annotated MS[2] spectrum (ESI-, mean collision energy: 77.5 V) of HS7a (m/z = 179.0126, RT = 4.9 min, Confidence Level 4) in MeOH extract of T300 B.

HS7b

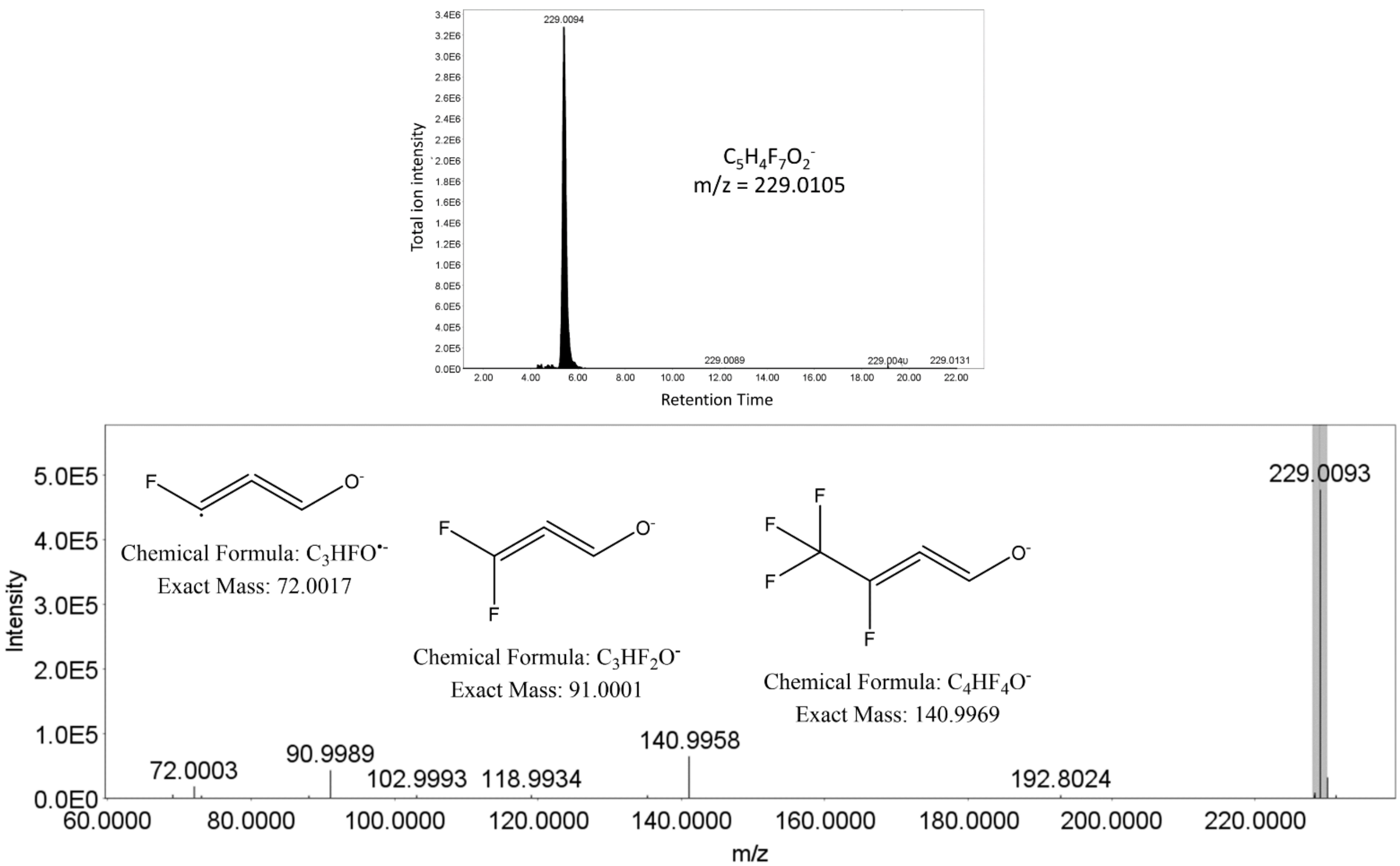

Figure S35: Chromatogram with proposed chemical formula and annotated MS^2 spectrum (ESI-, mean collision energy: 77.5 V) of HS7b (m/z = 229.0094, RT = 5.4 min, Confidence Level 4) in MeOH extract of T300 B.

HS7c

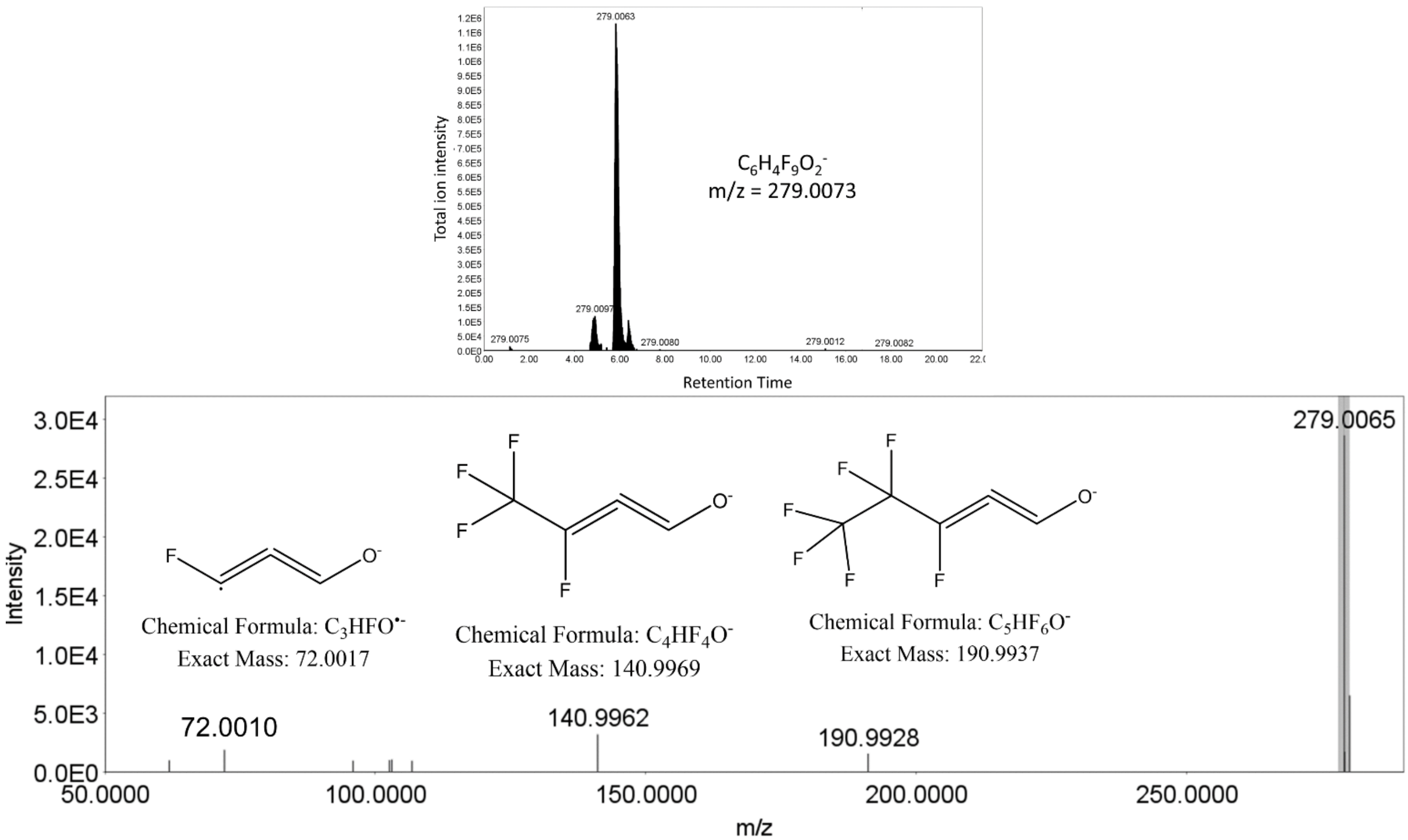

Figure S36: Chromatogram with proposed chemical formula and annotated MS^2 spectrum (ESI-, mean collision energy: 77.5 V) of HS7c (m/z = 279.0062, RT = 5.9 min, Confidence Level 4) in MeOH extract of T300 B.

HS7d

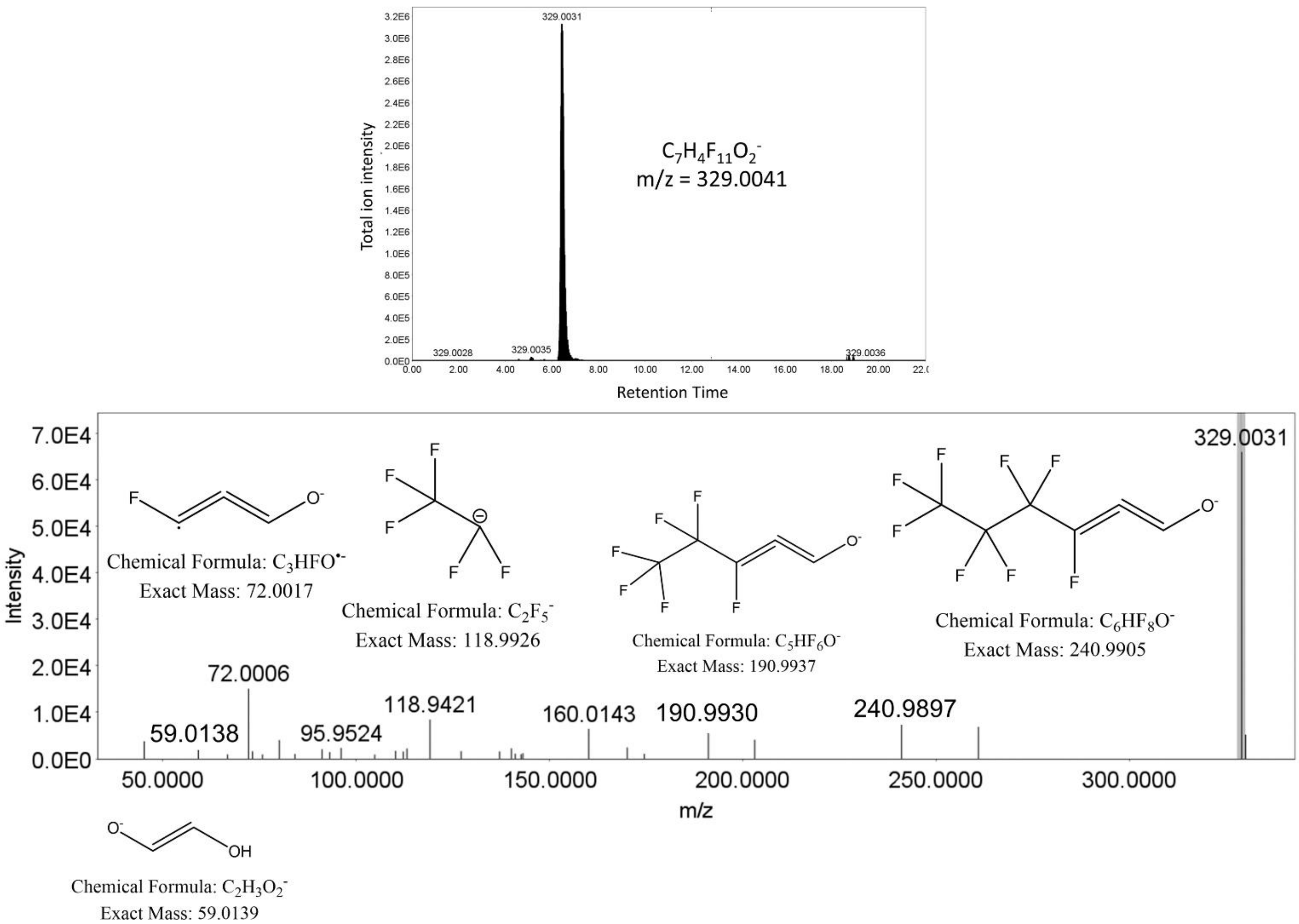

Figure S37: Chromatogram with proposed chemical formula and annotated MS^2 spectrum (ESI-, mean collision energy: 77.5 V) of HS7d (m/z = 329.0031, RT = 6.4 min, Confidence Level 4) in MeOH extract of T300 B.

HS8a

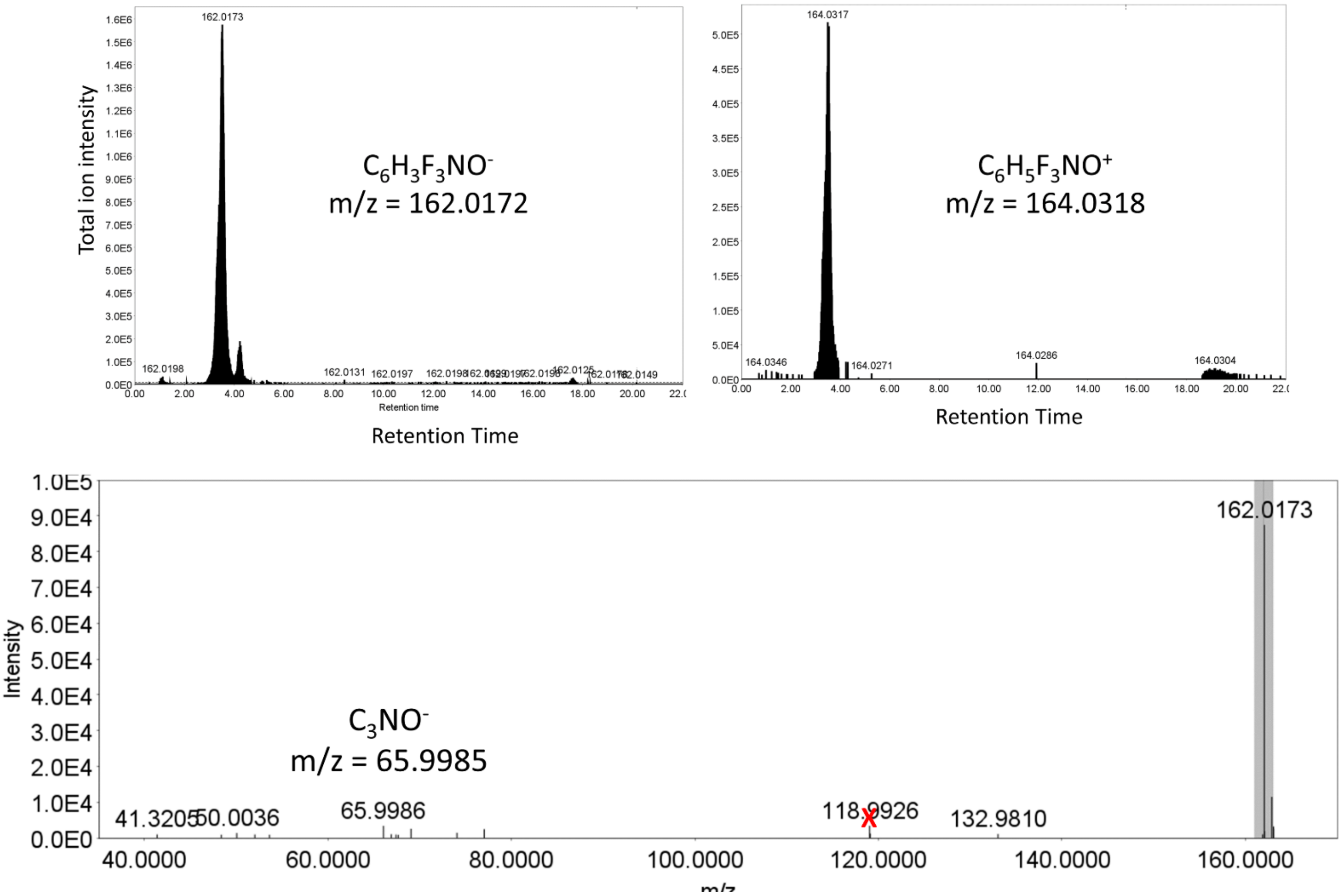

Figure S38: Chromatogram with proposed chemical formula (ESI+/-) and annotated MS^2 spectrum (ESI-, mean collision energy: 77.5 V) of HS8a (m/z = 162.0172/164.0318, RT = 3.5 min, Confidence Level 4) in MeOH extract of T300 B.

HS8b

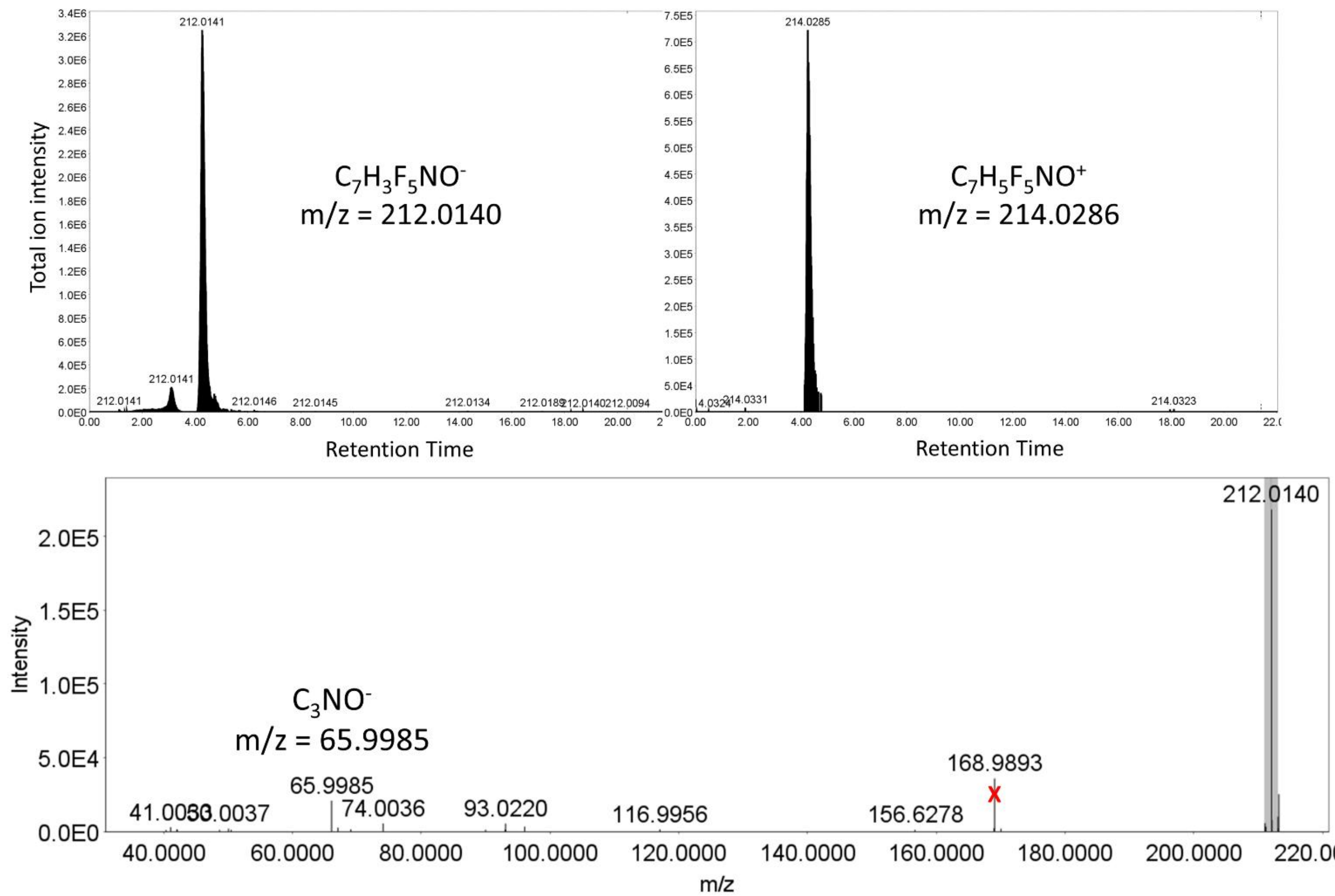

Figure S39: Chromatogram with proposed chemical formula (ESI+/-) and annotated MS^2 spectrum (ESI-, mean collision energy: 77.5 V) of HS8b (m/z = 212.0141/214.0286, RT = 4.3 min, Confidence Level 4) in MeOH extract of T300 B.

HS8c

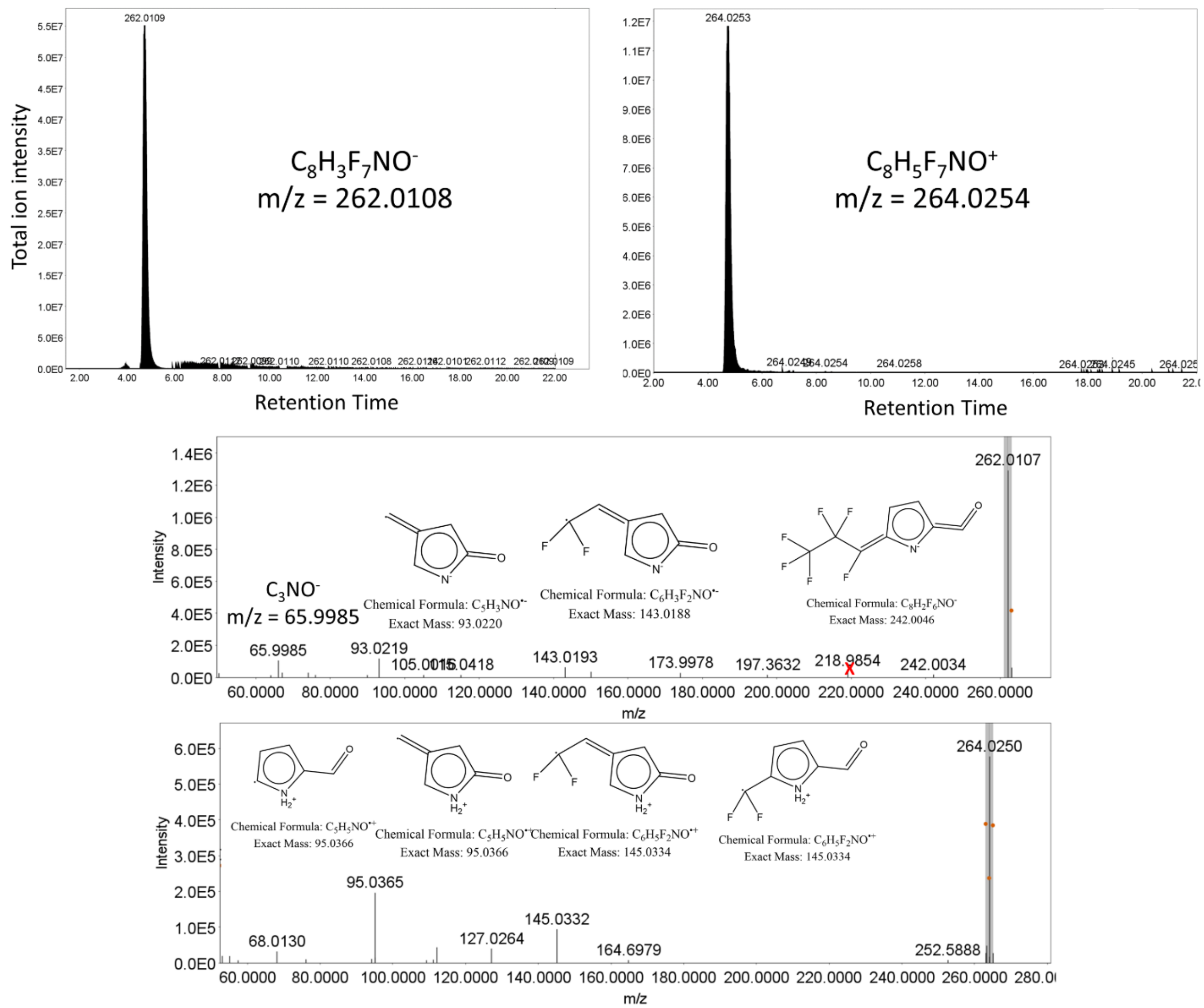

Figure S40: Chromatogram with proposed chemical formula (ESI+/-) and annotated MS^2 spectrum (ESI+/-, mean collision energy: 77.5 V) of HS8c (m/z = 262.0108/264.0254, RT = 4.7 min, Confidence Level 4) in MeOH extract of T300 B.

PID15

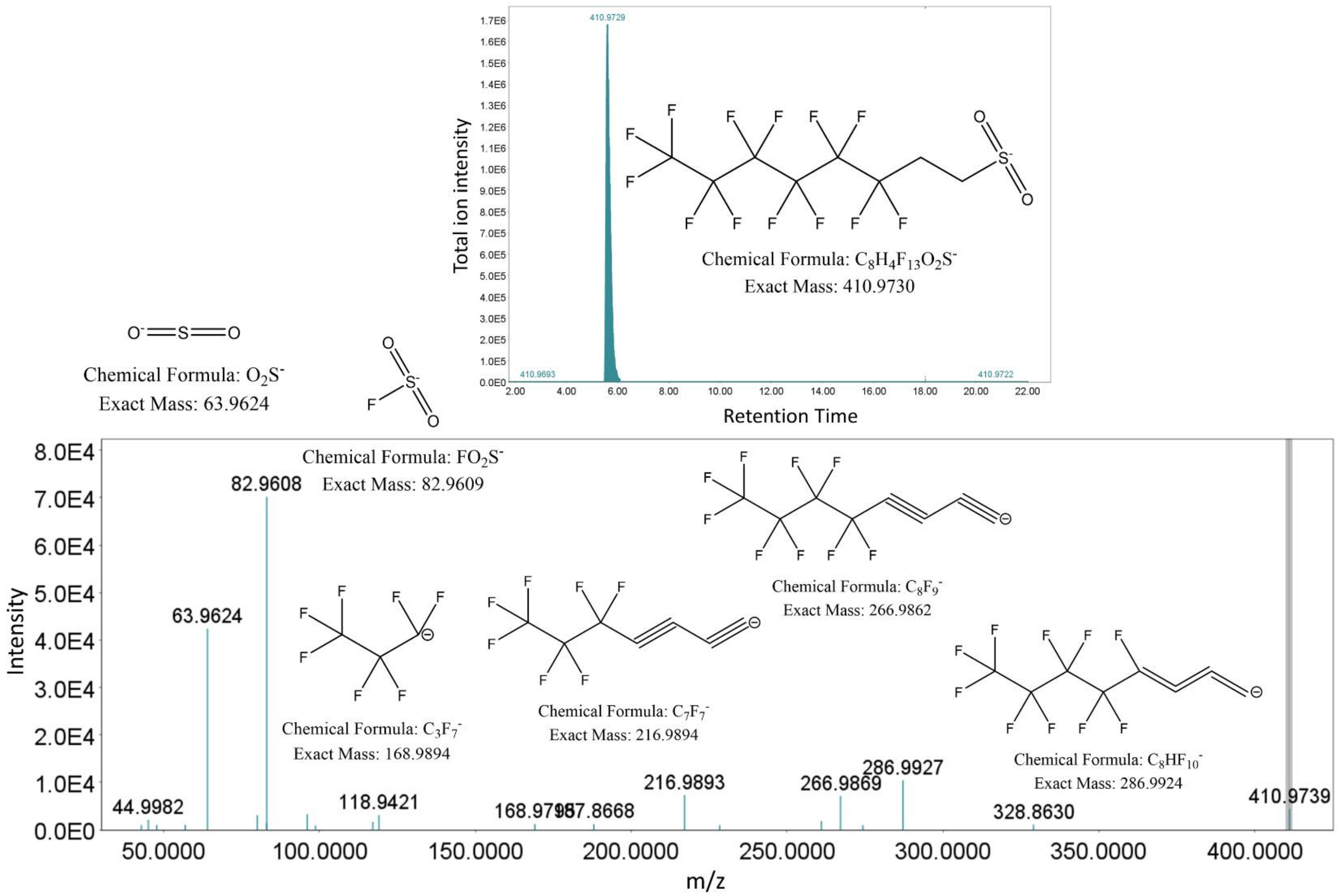

Figure S41: Chromatogram with tentative identified structure and annotated MS^2 spectrum (ESI-, mean collision energy: 77.5 V) of PID15 (m/z = 410.973, RT = 4.7 min, Confidence Level 2) in MeOH extract of T200 B (1:100 dilution).

PID16

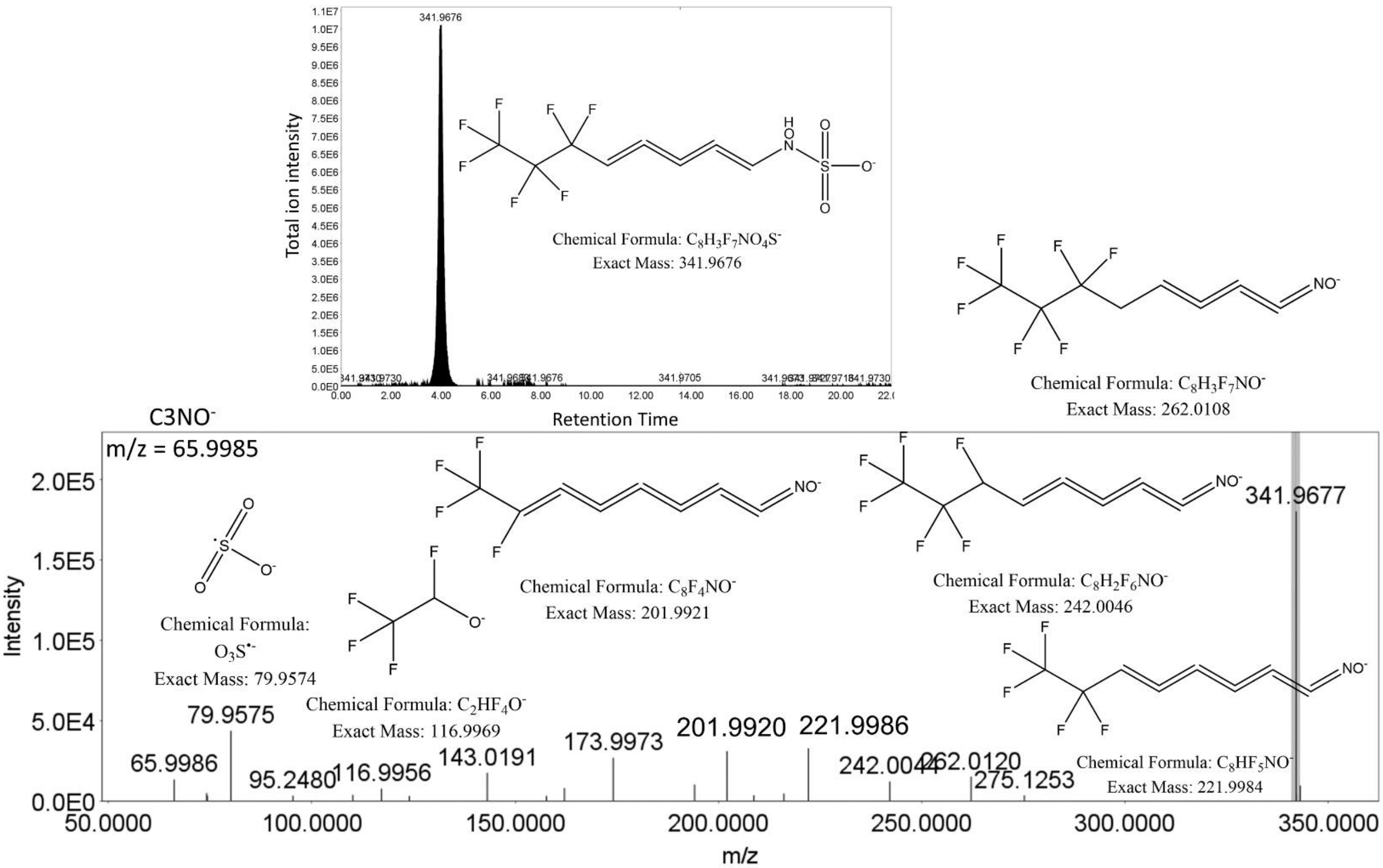

Figure S42: Chromatogram with tentative identified structures and annotated MS^2 spectrum (ESI-, mean collision energy: 77.5 V) of PID16 (m/z = 341.9676, RT = 4.0 min, confidence Level 2) in MeOH extract of T300 B

PID17

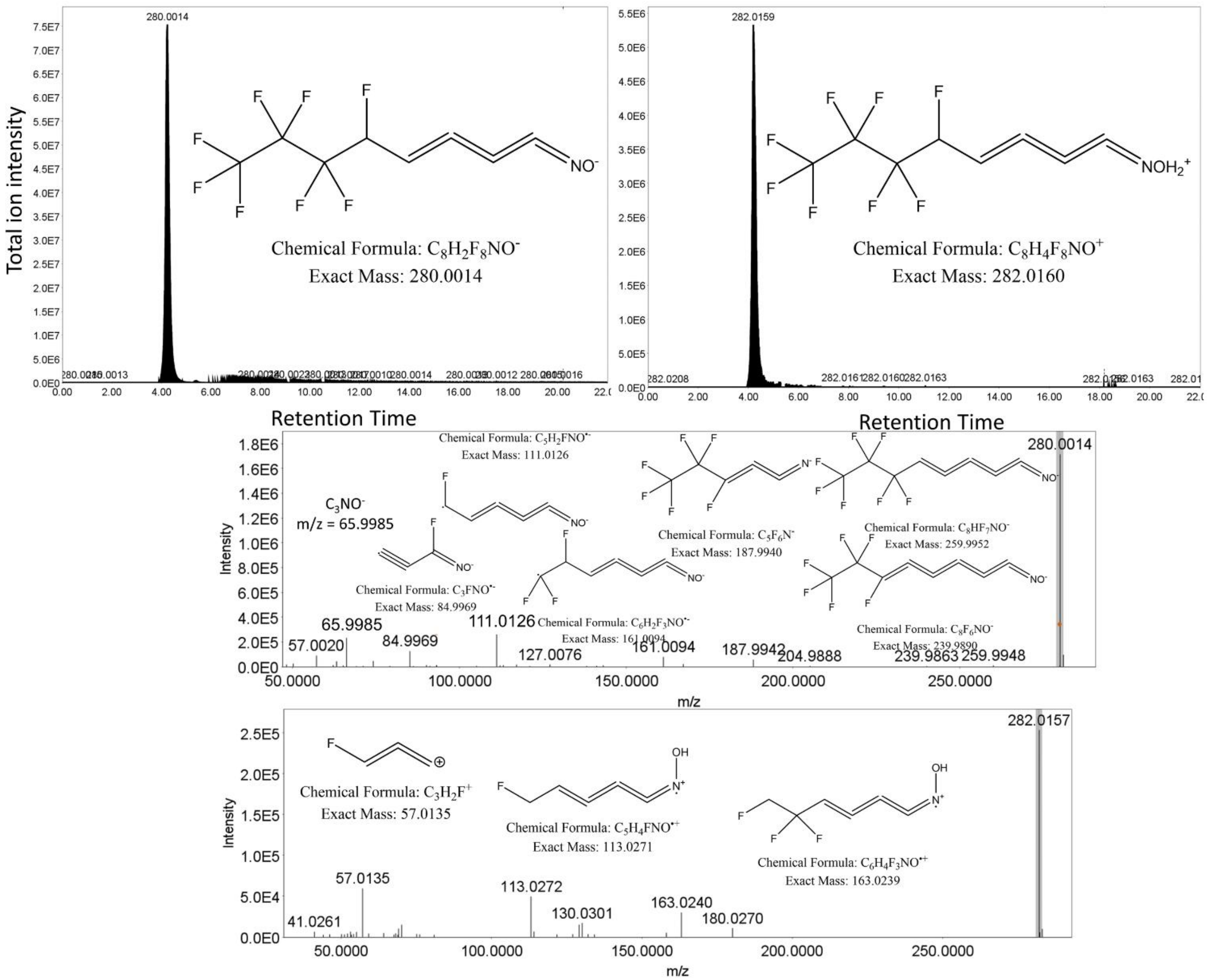

Figure S43: Chromatogram with tentative identified structures (ESI+/-) and annotated MS^2 spectrum (ESI+/-, mean collision energy: 77.5 V) of PID17 (m/z = 280.0014/282.0160, RT = 4.2 min, Confidence Level 2) in MeOH extract of T300 B.

PID18

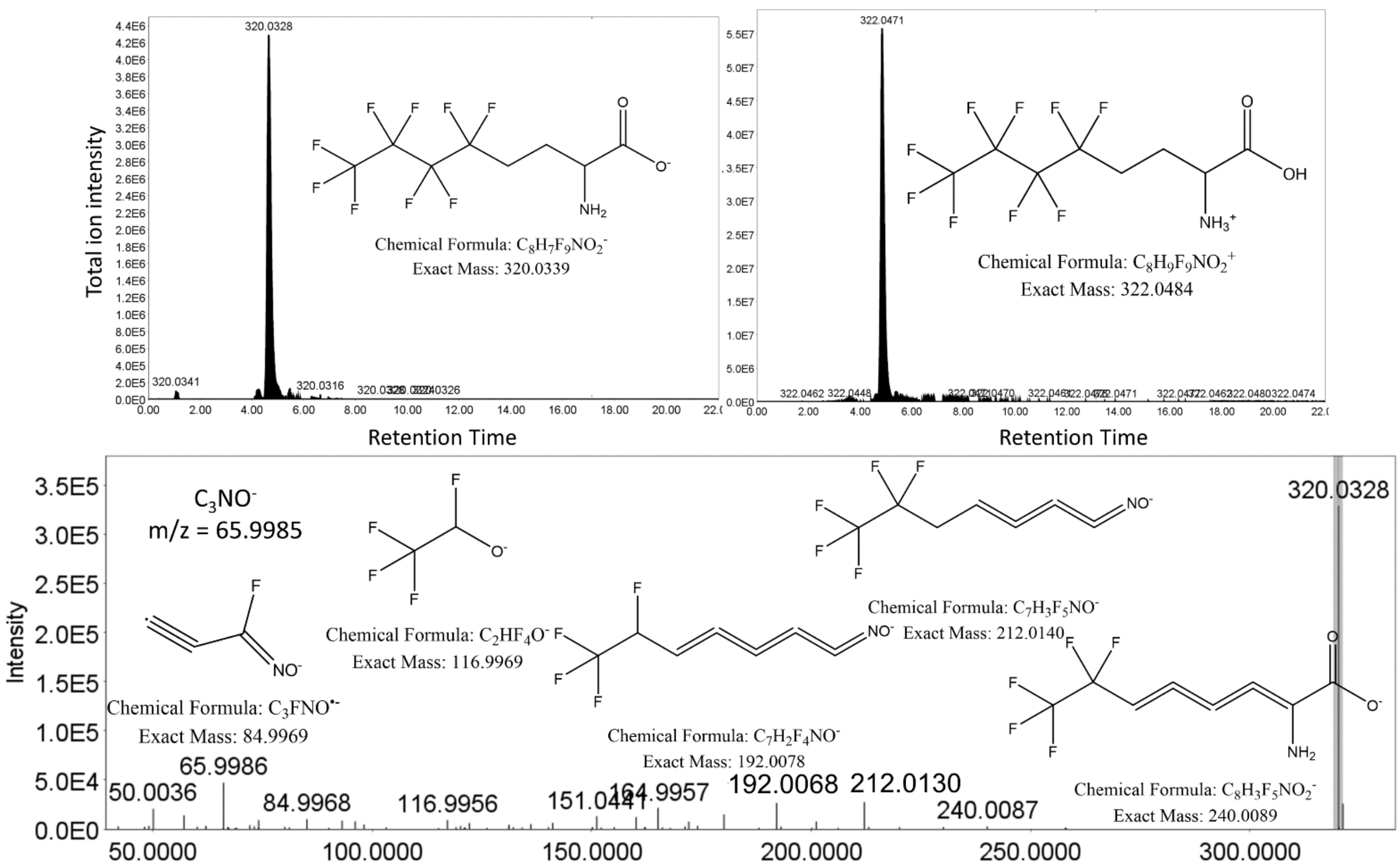

Figure S44: Chromatogram with tentative identified structures (ESI+/-) and annotated MS^2 spectrum (ESI-, mean collision energy: 77.5 V) of PID18 (m/z = 320.0328/322.0484, RT = 4.7 min, confidence Level 3) in MeOH extract of T300 B.

PID19

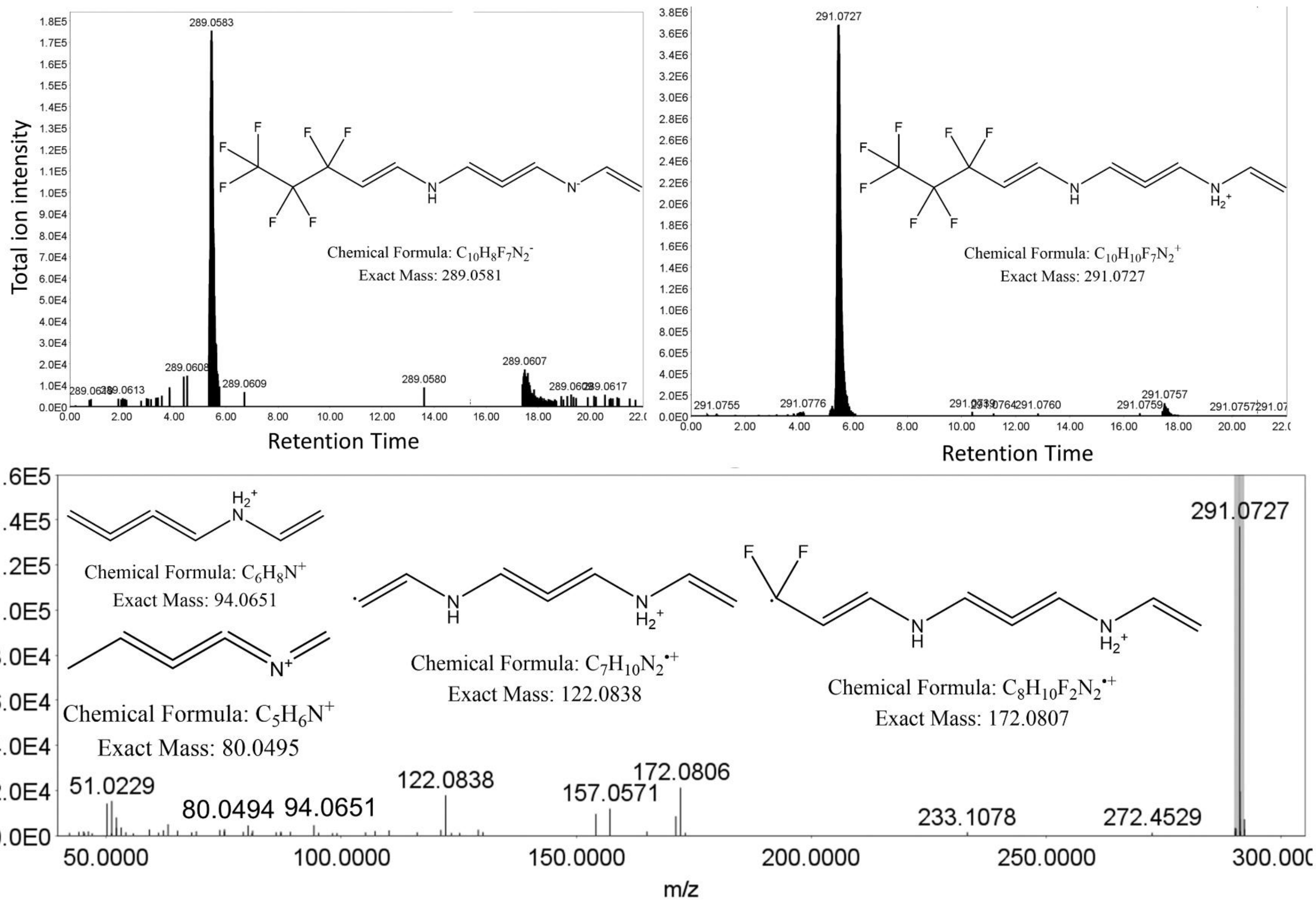

Figure S45: Chromatogram with tentative identified structures (ESI+/-) and annotated MS^2 spectrum (ESI+, mean collision energy: 77.5 V) of PID19 (m/z = 289.0582/291.0726, RT = 5.5 min, Confidence Level 3) in MeOH extract of T300 B.

PID20

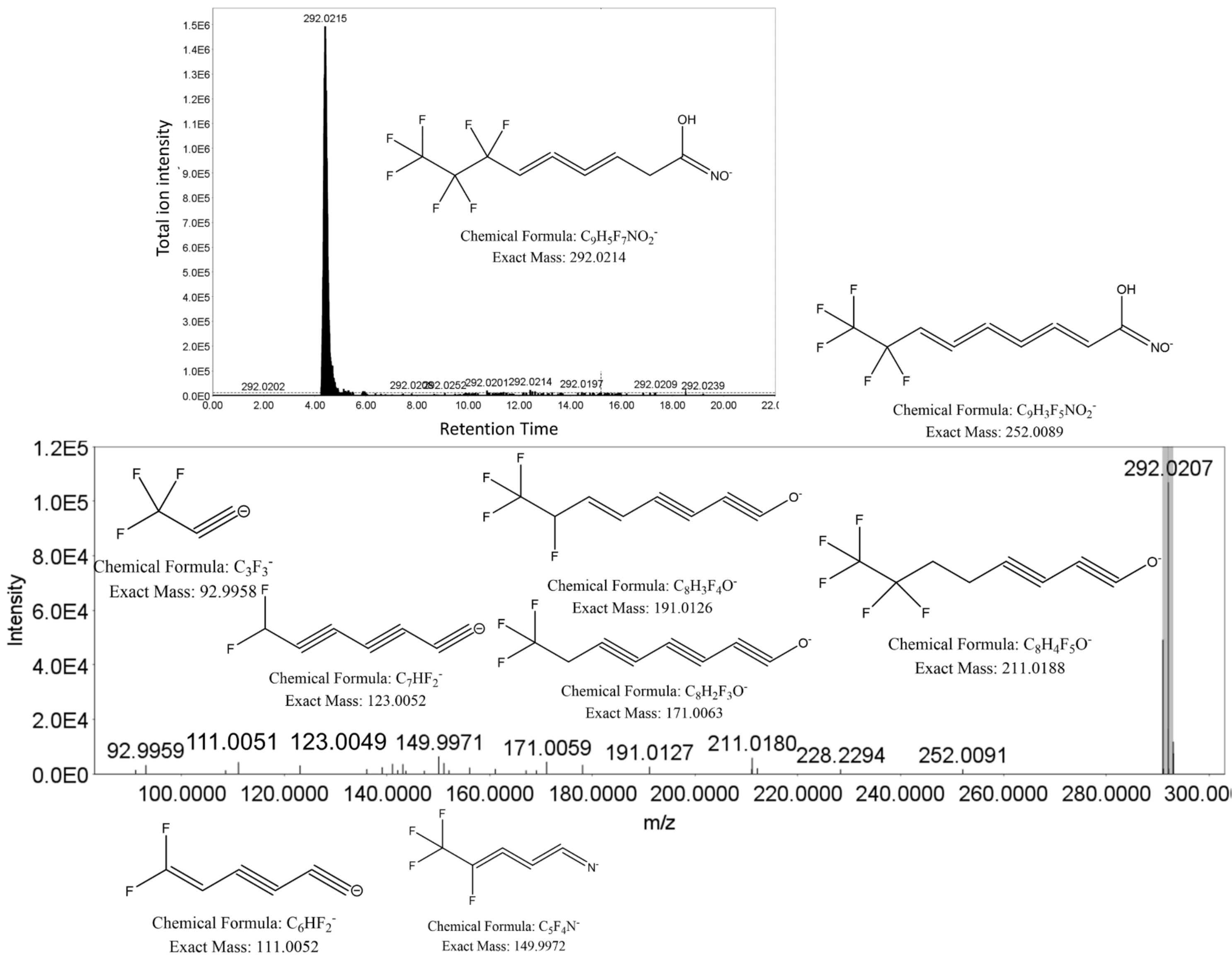

Figure S46: Chromatogram with tentative identified structures and annotated MS^2 spectrum (ESI-, mean collision energy: 77.5 V) of PID20 (m/z = 292.0211, RT = 4.4 min, Confidence Level 3) in MeOH extract of T300 B.

PID21

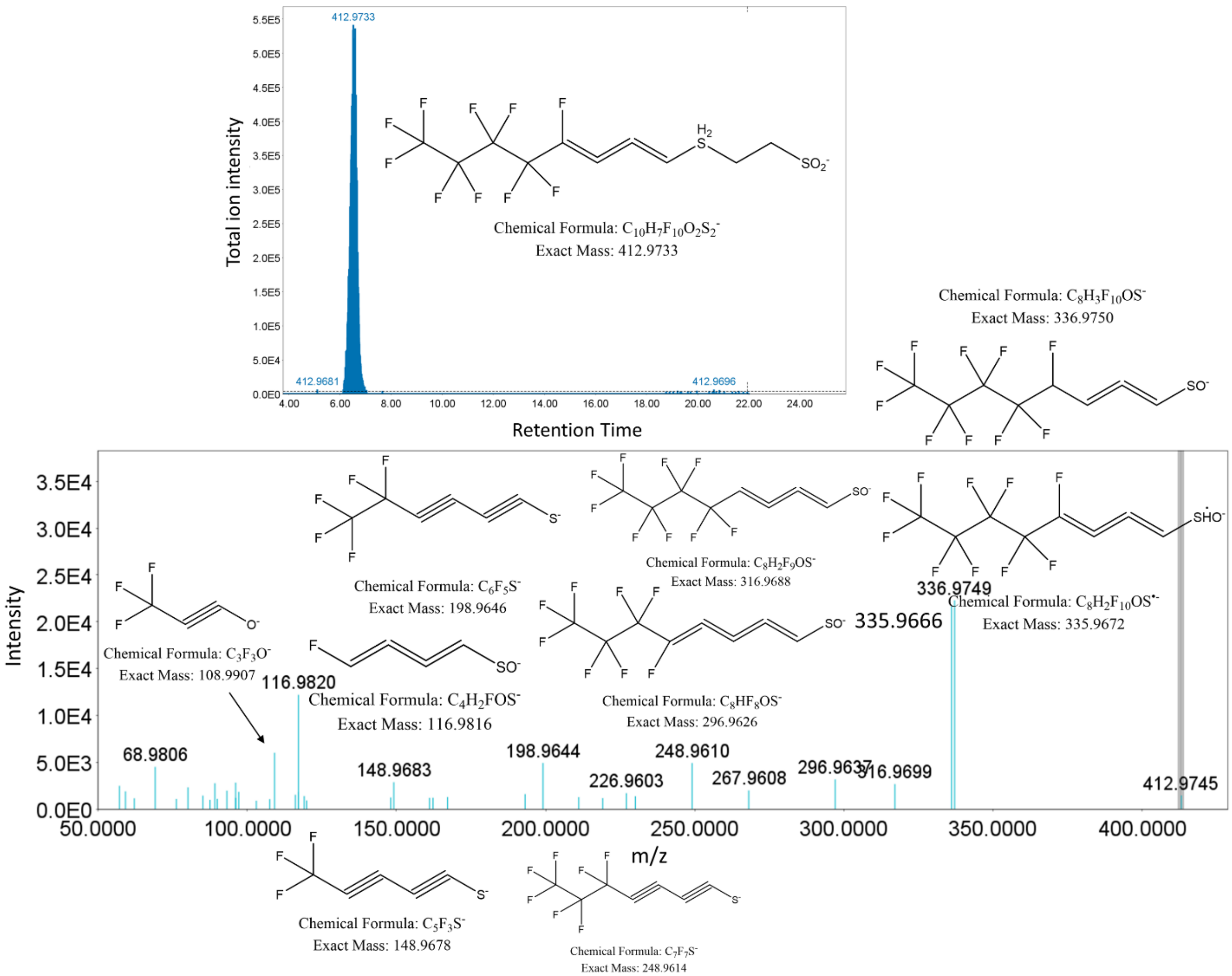

Figure S47: Chromatogram with tentative identified structure (T400 B) and annotated MS^2 spectrum (ESI-, mean collision energy: 77.5 V) of PID21 (m/z = 412.9733, RT = 6.5 min, Confidence Level 3) in MeOH extract of T400 A.

PID22

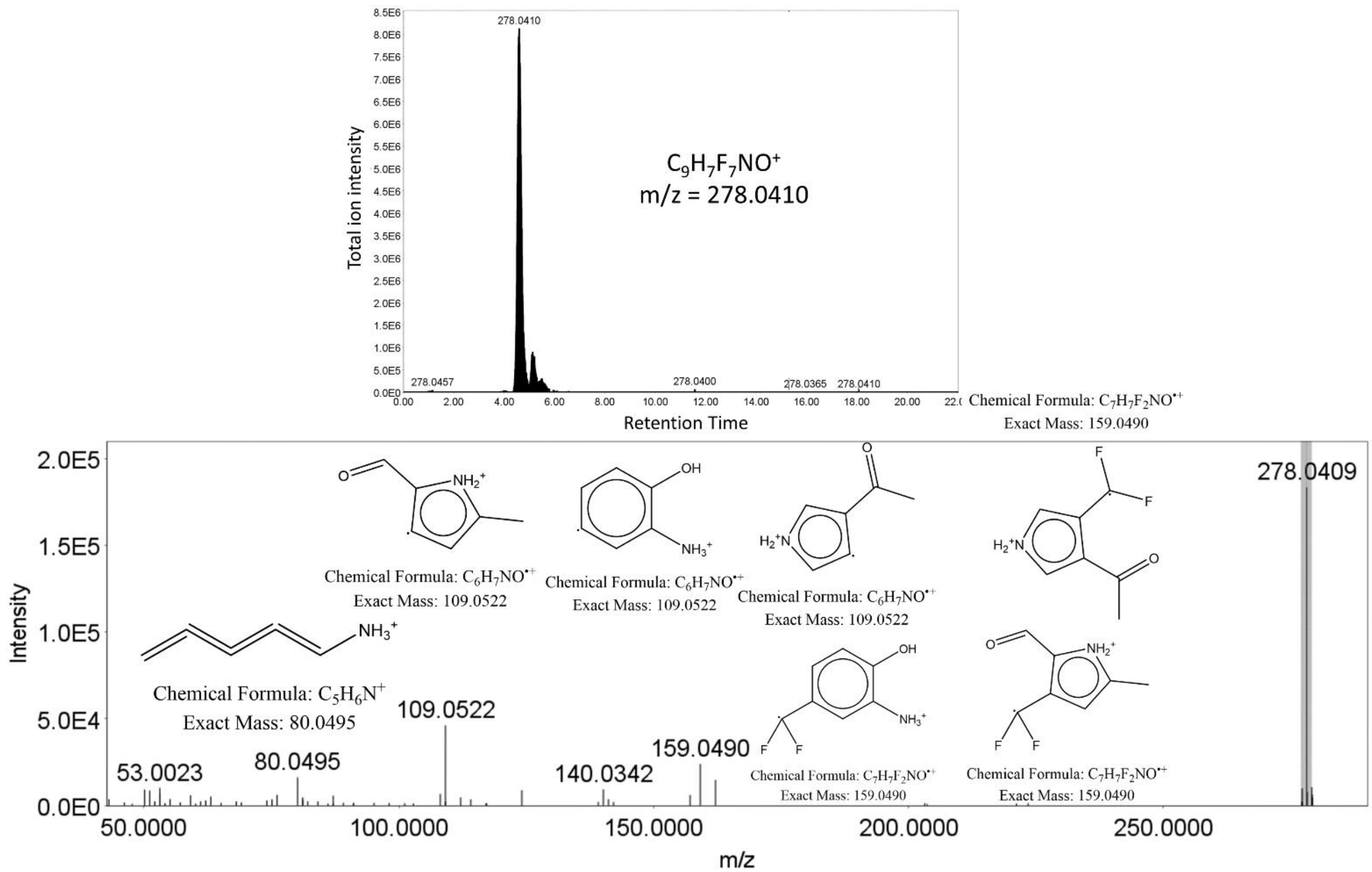

Figure S48: Chromatogram with proposed chemical formula and annotated MS^2 spectrum (ESI+, mean collision energy: 77.5 V) of PID22 (m/z = 278.041, RT = 4.3 min, Confidence Level 4) in MeOH extract of T300 B.

References

1. Haußecker M, Bertels S, Oró-Nolla B, Zwiener C (2026) Implementation of a mixed-mode liquid chromatography-based analysis to assess fluorinated inorganic anions and (ultra)-short chain PFAS con-tamination in surface waters, *in preparation*.
2. Capitain C, Schüßler M, Bugsel B, Zweigle J, Vogel C, Leube P, Zwiener C (2025) Implementation of Matrix-Matched Semiquantification of PFAS in AFFF-Contaminated Soil. Environ Sci Technol. doi:10.1021/acs.est.4c14255
